# Tailoring Polymer Properties Through Lignin Addition: A Recent Perspective on Lignin-Derived Polymer Modifications

**DOI:** 10.3390/molecules30112455

**Published:** 2025-06-03

**Authors:** Nawoda L. Kapuge Dona, Rhett C. Smith

**Affiliations:** Department of Chemistry, Clemson University, Clemson, SC 29634, USA; dkapuge@g.clemson.edu

**Keywords:** lignin, polyurethane, epoxy resins, phenol-formaldehyde resin, PLA, films, additives

## Abstract

Lignin, an abundant and renewable biopolymer, has gained significant attention as a sustainable modifier and building block in polymeric materials. Recent advancements highlight its potential to tailor mechanical, thermal, and barrier properties of polymers while offering a greener alternative to petroleum-based additives. This review provides an updated perspective on the incorporation of lignin into various polymer matrices, focusing on lignin modification techniques, structure–property relationships, and emerging applications. Special emphasis is given to recent innovations in lignin functionalization and its role in developing high-performance, biodegradable, and recyclable materials such as polyurethanes, epoxy resins, phenol-formaldehyde resins, lignin-modified composites, and lignin-based films, coatings, elastomers, and adhesives. These lignin-based materials are gaining attention for potential applications in construction, automated industries, packaging, textiles, wastewater treatment, footwear, supporting goods, automobiles, printing rollers, sealants, and binders.

## 1. Introduction

**Figure 1:**
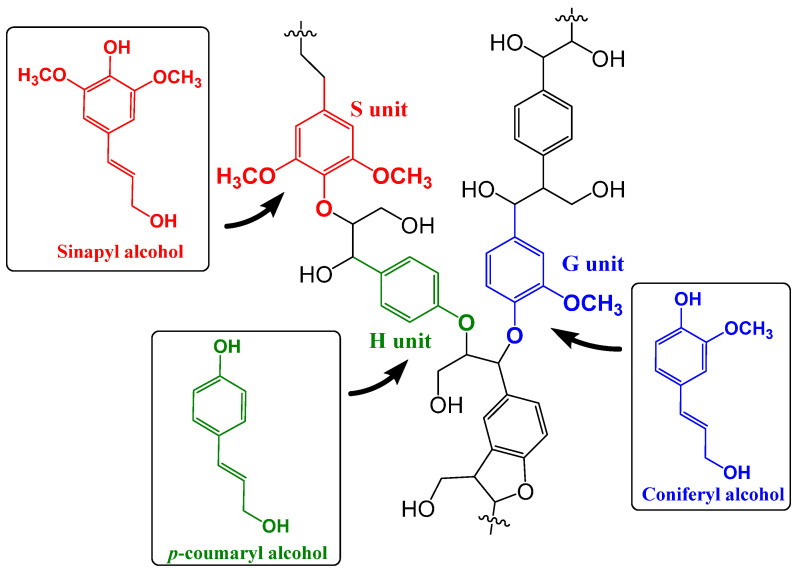
Schematic representation of the structure of lignin, composed of monolignol units.

Lignin is a complex organic biopolymer found in the plant cell walls, providing rigidity and resistance to decay. It is an abundant byproduct of the pulp and paper industry (~50 million tons of lignin), ethanol and textile industry (~30 million tons of lignin), and the agricultural sector [1,2]. However, less than 2% of lignin from these sources is currently utilized, with the remaining predominantly burned as low-cost fuel, raising significant environmental concerns [3,4,5]. Therefore, converting lignin into value-added products is crucial for addressing future economic and ecological challenges. Lignin is mainly composed of monolignol units [*p*-hydroxyphenyl (H), guaiacyl (G), and syringyl (S)]. interconnected through aryl ether or carbon–carbon bonds to form their three-dimensional (3D) network (Figure 1) [6]. The various functionalized aromatic groups present in lignin make it a promising candidate to replace petrochemicals. Additionally, lignin is naturally hydrophilic, and its rigid structure imparts unique properties to polymer matrices.

Due to its aromatic hydroxyl functionalities and abundant π–π conjugation, lignin offers biocompatibility, flame retardancy, UV resistance, antibacterial and antioxidant activities, thermal stability, and the ability to modify mechanical properties [7,8,9]. These characteristics have made lignin the focus of numerous studies and reviews, highlighting its versatility as a polymer additive. It has been successfully incorporated into lignin-derived polyurethanes (PUs), epoxy resins, phenol-formaldehyde resins, polyesters, polylactic acid (PLA), bio-based fillers, additives, films, coatings, stimuli-responsive, and biomedical materials [10,11,12,13,14,15,16,17,18,19,20,21,22]. The incorporation of lignin in these materials offers a more sustainable and often higher-performing alternative to their petrochemical-based counterparts [23,24].

Despite lignin’s abundance, its heterogeneity, complexity, and structural variability challenge its comprehensive study and utilization. To address these issues, lignin can be extracted, depolymerized, or chemically and physically modified to produce smaller derivatives with enhanced functionalities [25,26,27]. Also, depending upon the extraction method, lignin can be categorized into different types such as kraft lignin, alkali lignin, soda lignin, hydrolyzed lignin, etc., and the lignin composition is different in each lignin type [28,29,30]. Lignin can be discussed at several levels of structural complexity, which must be carefully distinguished. “Whole lignin” refers to the unmodified, extracted material, typically containing a wide molecular weight distribution and a mixture of structural subunits (e.g., kraft, soda, organosolv lignin). “Lignin fractions” are obtained through physical or chemical separation processes that reduce polydispersity or isolate particular molecular weight windows. “Lignin-derived monomers and oligomers”, on the other hand, are breakdown products obtained by depolymerization techniques such as oxidative cleavage, hydrogenolysis, or solvolysis. These distinct forms vary not only in reactivity and solubility but also in their suitability for incorporation into polymer matrices. As such, clarity in terminology is critical when interpreting or comparing the literature on lignin-based polymers.

This review aims to classify and analyze current strategies for lignin incorporation into polymer matrices across diverse synthetic routes and applications, including polyurethanes (PUs), resins, PLA composites, and lignin-based films, coatings, and adhesives. Emphasis is placed on chemical modifications, structure–property relationships, and sustainability metrics. Moreover, lignin can be utilized in more applications, ranging from hydrogels or aerogels [31,32,33,34,35,36], capacitors, or lithium–sulfur batteries, [37,38,39,40,41], light-driven shape memory polymers [42,43], pH-sensitive lignin-based polymers, [44]. several nano lignin applications [45,46,47,48,49], and as an application in high-sulfur content materials [50,51,52,53,54], which will not be discussed in this review.

## 2. Lignin as a Precursor in Polyurethanes (PUs)

**Scheme 1:**
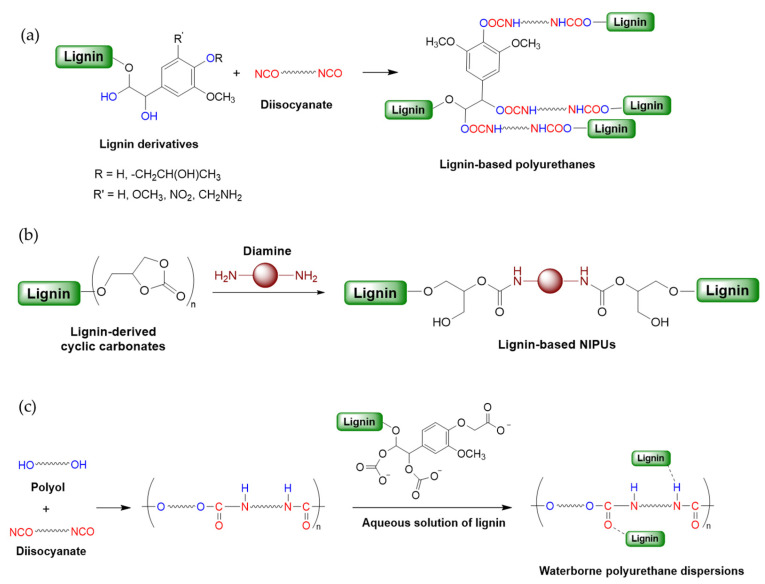
General reaction scheme for lignin-based polyurethane formation. (**a**) Traditional isocyanate pathway from unmodified or modified lignin (**b**) Non-isocyanate pathway from cyclic carbonate functionalized lignin and amines (**c**) Waterborne polyurethane synthesis pathway from auqeous solution of lignin.

Polyurethane (PU) is one of the most versatile polymers, and it is used in different industries, including coating, adhesive, textile, foams, sealants, automotive, and aerospace [55,56,57]. PUs are synthesized through a step-growth addition polymerization process by reacting polyols (compounds containing multiple hydroxyl groups) with diisocyanates (compounds containing two isocyanate groups). The reaction typically involves the formation of urethane linkages. Traditional PU production relies on petroleum-based polyols, but the hydroxyl groups in lignin provide an opportunity for its utilization as a renewable polyol alternative, providing biodegradability, cost-effectiveness, and reduced environmental impact (Scheme 1a). Despite its potential, lignin’s complex, irregular structure and low reactivity due to limited available hydroxyl groups hinder its direct use. To enhance its compatibility and reactivity with isocyanates, lignin is often chemically modified through hydroxy alkylation, phenolation, methylation, esterification, and amination methods to increase the number of accessible hydroxyl groups and improve solubility and dispersion within polymer matrices [58,59,60]. Such modifications significantly improve the performance and processability of lignin-based PUs (Table 1). This section will explore the most recent approaches to integrating unmodified and modified lignin into PU formulations, including synthesis pathways of lignin-based non-isocyanate and waterborne polyurethanes (Scheme 1).

### 2.1. Unmodified Lignin-Based Polyurethanes

**Figure 2:**
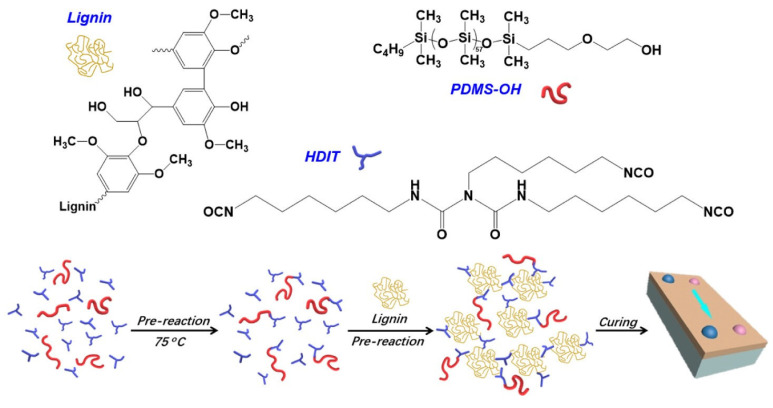
Preparation method for lignin-based PU coating (lig-PU-PDMS) [61]. Used with permission, copyright 2023 American Chemical Society.

Chen et al. developed a lignin-based PU coating with anti-smudge and anti-UV properties in their study [61]. Lignin-based coatings (lig-PU-PDMS) were prepared using lignin as polyols, hexamethylene diisocyanate trimer (HDIT) as the crosslinking agent, and a small amount of monocarbinol-terminated poly-dimethylsiloxane (PDMS-OH) as the dewetting agent (Figure 2). The hardness (HIT) and elastic modulus (EIT) of lig-PU-PDMS coatings were measured by nanoindentation. When the PDMS-OH content increased from 0 to 5.0 wt.%, HIT decreased from 260.1 to 226.8 MPa, and EIT decreased from 4.7 to 4.1 GPa. After 400 h of UV aging, the HIT and EIT of lig-PU-PDMS (0 wt.%) increased by 16.9% and 16.1%, respectively, while those of lig-PU-PDMS (5.0 wt.%) increased by only 11.9% and 10.9%. Moreover, the coatings maintained good mechanical properties even after UV aging, with lignin protecting the PU from UV damage. The aromatic skeleton and conjugated structure of lignin provide the coatings with extraordinary UV-blocking capability and increase the strength of the PU coating. However, the study focused on a specific lignin type, indicating that further research may be necessary to adjust the process for other lignin sources.

**Figure 3:**
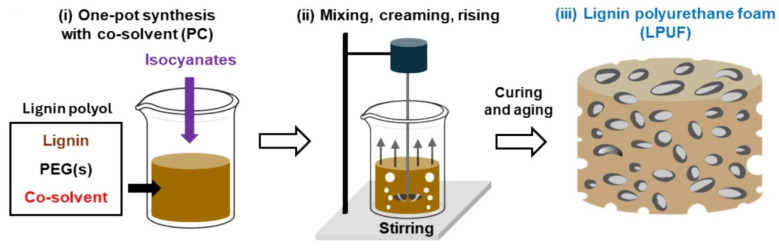
Illustration of the one-pot synthesis of lignin polyurethane foam using a co-solvent [62]. Used with permission, copyright 2024 Elsevier.

In another study, Zhang and coworkers introduced a method to address the high viscosity of lignin, which limits its processability and application in lignin-based polyurethane foams (PUFs) [62]. Organosolv lignin was dissolved in green co-solvent (propylene carbonate) to reduce the viscosity and reacted with diphenylmethane-4,4′-diisocyanate (MDI) to form the lignin-based PUFs using a one-pot synthesis method (Figure 3). The lignin content varied from 20 to 80 wt.% and equal weights of propylene carbonate and lignin were used. Compared to mixtures without the co-solvent, those with the co-solvent exhibited lower viscosity, even at higher lignin content. Thermogravimetric analysis (TGA) showed increased thermal stability and higher char yield formation with lignin addition, attributed to its aromatic structure. Additionally, the lignin-based PUFs demonstrated enhanced hydrophobicity, with the water contact angle rising from 100 to 140 degrees as lignin content increased. Mechanical analysis indicated that, without the co-solvent, PUFs with low lignin content had slightly higher strength than the control PUF due to mechanical interlocking and hydrogen bonding between lignin and the matrix. However, higher lignin content reduced strength, as the brittle PU cells and non-uniform foam structure acted as rigid foams. In contrast, higher lignin content with the co-solvent resulted in greater strength than the control PUF. The study successfully demonstrated the substitution of (up to 80 wt.%) petroleum polyol with unmodified lignin, preventing viscosity issues and maintaining favorable properties.

**Figure 4:**
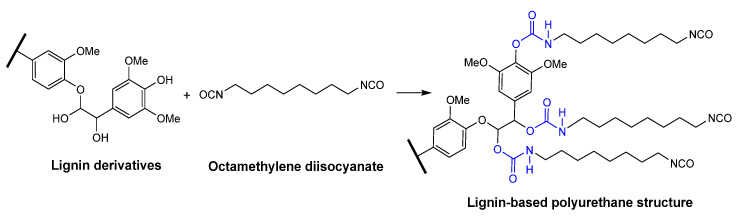
The reaction mechanism between lignin derivatives and octamethylene diisocyanate. Used with permission, copyright 2024 Elsevier.

The inherent complexity and heterogeneity of lignin can lead to inconsistent performance in polyurethane applications when it is directly incorporated into the formulation without prior modification. To address this issue, Li et al. fractionated industrial alkali lignin using green solvents to study the effect of different molecular weight fractions on polyurethane foam (PUF) properties [63]. The dispersity and hydroxy content gradually decreased during fractionation, yielding lower molecular weight lignin in the initial steps. Fractionated lignin was used as a polyol source, with octamethylene diisocyanate as the isocyanate to synthesize PUFs (Figure 4). The impact of different lignin fractions was studied using 2 wt.% of lignin, and the substitution degree of lignin was evaluated using the lowest molecular weight fraction, which contained higher hydroxy group content. Lignin derivatives from low-molecular-weight fractions formed flexible PU, while the high molecular weight of lignin fractions formed rigid PU due to their high aromatic density. Lower molecular weight lignin improved mechanical strength, while higher molecular weight lignin enhanced thermal stability. When the substitution degree of lignin increased from 2% to 30%, elongation at break peaked at 834% with 5% substitution, then declined. Tensile strength increased with a 15% lignin substitution before decreasing with further additions.

**Figure 5:**
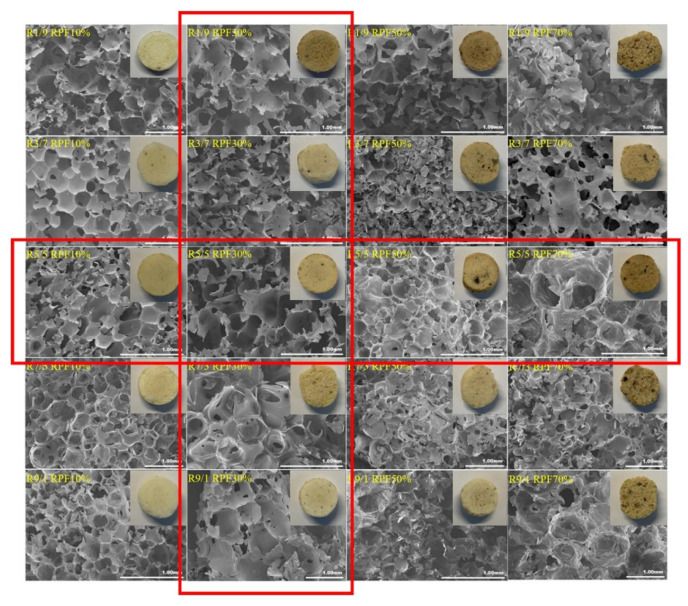
SEM images of RPFs synthesized using catalytic upstream biorefining (CUB) lignin oligomers as polyol substitutes [64]. Used with permission, copyright 2023 Elsevier.

In another study, lignin oligomers derived from poplar sawdust through catalytic upstream biorefining (CUB) were used as a partial polyol substitute for synthesizing rigid polyurethane foams (RPFs) [64]. The lignin oligomers were extracted from crude CUB oils produced with varying 2-propanol/water ratios (1/9, 3/7, 5/5, 7/3, and 9/1 *v*/*v*) in the pulping liquors. These oligomers, rich in hydroxyl groups, reacted with isocyanates to create the polyurethane networks. The chemical composition of the pulping liquor significantly influenced the properties of the lignin oligomers and, consequently, the physical characteristics of the RPFs. Increased substitution of lignin oligomers led to an increase in apparent density but reduced compressive strength. Among all formulations, RPFs with 30% polyol substitution using lignin oligomers extracted with a 7/3 2-propanol/water ratio (R7/3) demonstrated the optimal balance, achieving the highest compressive strength and the lowest apparent density. Morphological analysis (Figure 5) revealed that these lignin oligomers, with their higher hydroxyl content, facilitated the formation of highly crosslinked nano-sized complexes, creating a stable polyurethane network that effectively supported the cellular foam structure.

### 2.2. Chemically Modified Lignin-Based Polyurethanes

**Figure 6:**
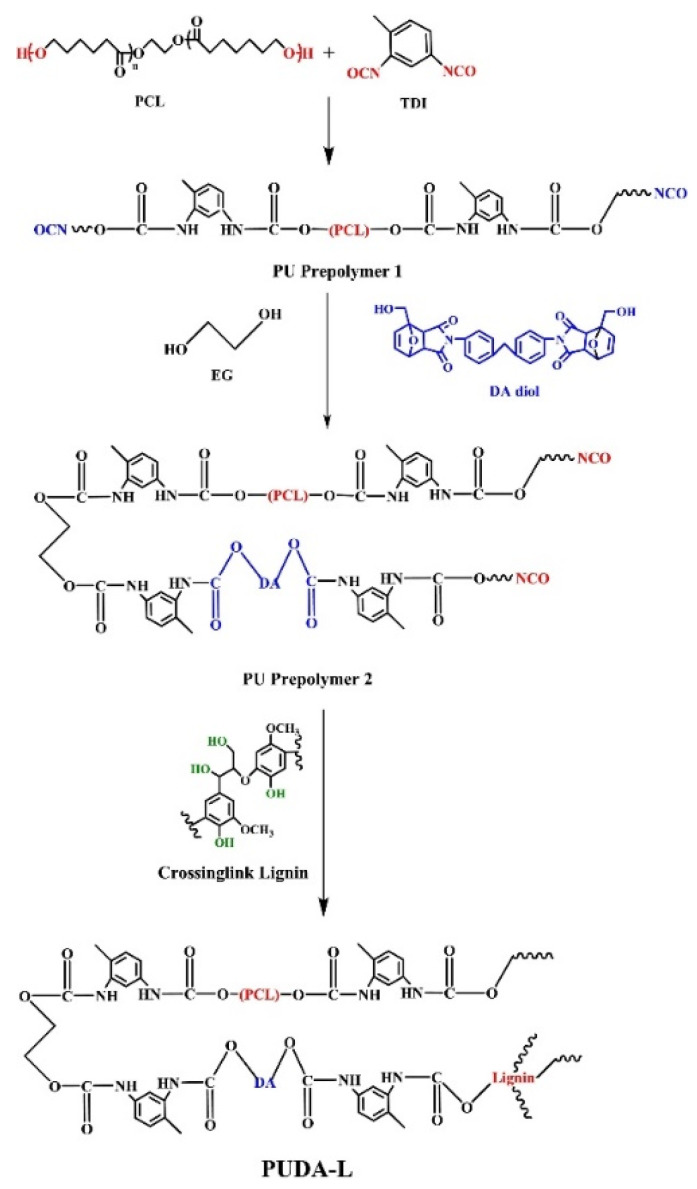
Preparation method of lignin self-healing polyurethane elastomer (PUDA-L) [65]. Used with permission, copyright 2023 Elsevier.

**Figure 7:**
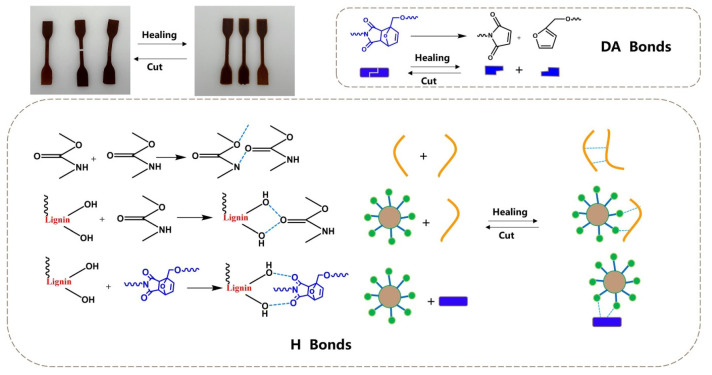
Self-healing mechanism of lignin-based self-healing polyurethane elastomer [65]. Used with permission, copyright 2023 Elsevier.

Du et al. developed a lignin-based self-healing polyurethane elastomer (PUDA-L) by integrating hydroxylated-modified lignin, Diels–Alder (DA) bonds, and hydrogen bonds into the polymer matrix [65]. Hydroxylation modification of lignin increases its hydroxyl content, enhancing crosslinking density and improving mechanical properties. At the molecular level, hydroxylation increases the number of reactive –OH groups on lignin, enhancing its ability to form urethane bonds with isocyanates. This leads to a denser crosslinked network, improving tensile strength and elasticity. The addition of DA diol as a chain extender promotes self-healing properties, while polycaprolactone (PCL) was selected as the soft segment due to its ability to enhance melting crystallization and self-healing efficiency (Figure 6). Figure 7 illustrates the self-healing mechanism of elastomers, which is driven by multiple molecular interactions. Lignin incorporation improves the thermal stability and rigidity of polyurethane, leading to higher glass transition (*T*_g_) and energy storage moduli (E’). However, in DA-modified elastomers, the overall impact of lignin on thermal stability is less pronounced due to the strong crosslinking network. Mechanical testing showed that an optimal PCL–DA diol–TDI (toluene diisocyanate) ratio of 1:1:2.5 maximized tensile strength (25.2 MPa), while excessive or insufficient DA diol and higher lignin addition negatively affected mechanical performance. The optimized PUDA-L formulation achieved an elongation at a break of 500% and a self-healing efficiency of up to 100%, offering excellent durability, flexibility, and self-healing capabilities. The study provides valuable insights into the development of bio-based, high-performance polyurethane elastomers for applications in innovative coatings, sensors, and flexible electronics.

**Figure 8:**
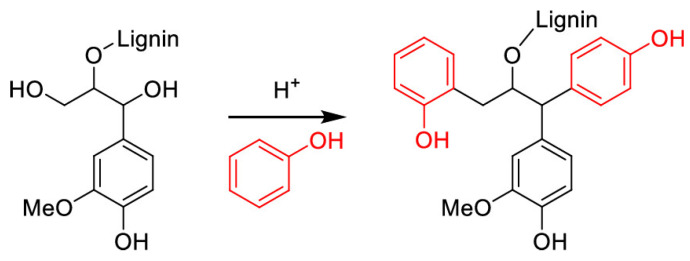
The replacement of aliphatic hydroxyl groups by ortho-/para-bound phenol through acid-catalyzed phenolation of lignin [66]. Used with permission, copyright 2024 American Chemical Society.

In another study, Falireas and coworkers employed an acid-catalyzed phenolation method (Figure 8) to modify lignin chemically, enhancing its reactive sites for polyurethane (PU) synthesis [66]. The acid-catalyzed phenolation increases the number of phenolic hydroxyl groups in lignin, enhancing its nucleophilicity and reactivity with isocyanate groups. This leads to more urethane bonds and higher crosslinking density in the polymer network. The enhanced mechanical and adhesive properties observed in pMDI-based systems can be attributed to strong interactions between the aromatic rings of phenolated lignin and pMDI, including π–π stacking and hydrogen bonding. This molecular interaction causes the superior performance of the phenolated lignin-based PU framework. Hydrolysis lignin (HL) was used as the lignin source for polymer preparation. A series of phenolated lignin-based polyurethane (PhLPU) films were formulated using phenolated lignin (0–60 wt.% of PhL) and polytetrahydrofuran as a polyol mixture, with either an aromatic poly[(phenyl isocyanate)-*co*-formaldehyde]. (pMDI) or an aliphatic hexamethylene diisocyanate trimer (HDI) as the isocyanate source. The study examined the significant impact of PhL concentration and the chemical structure of the isocyanate on the thermal stability and mechanical performance of PU films. Thermal analysis results showed that increasing the phenolated lignin content enhanced the thermal stability of the films, with HDI-based films exhibiting a higher maximum decomposition temperature compared to those made with pMDI. Mechanical performance testing revealed that PhLPU films with pMDI demonstrated superior strength characteristics compared to films with HDI trimer. The adhesive strength was evaluated using single-lap shear tests, with PhLPU films exhibiting comparable shear strengths to commercially available PU adhesives. Overall, the study highlights the effectiveness of the phenolation process in increasing the chemical reactivity of lignin, facilitating the development of high-performance PU films and adhesives, and contributing to the advancement of sustainable material solutions.

**Figure 9:**
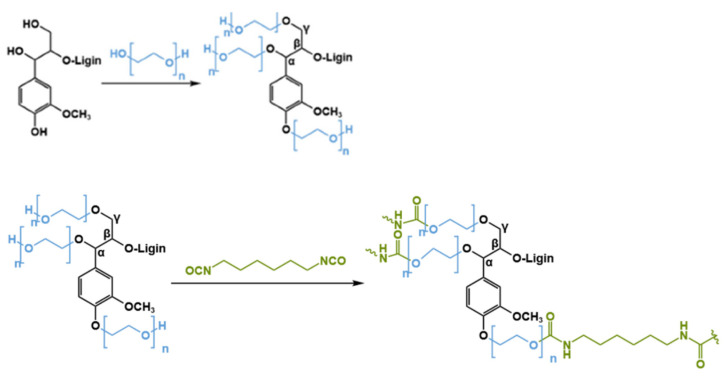
Lignin modification and preparation procedure of lignin-based polyurethane [67]. Used with permission, copyright 2024 American Chemical Society.

Liang et al. developed lignin-based polyols (LPs) by introducing hydroxyl groups into kraft lignin through liquefaction with polyethylene glycol (PEG). The PEG-mediated liquefaction involves acid-catalyzed transetherification between the ether linkages in PEG and the phenolic hydroxyl groups on lignin. This reaction yields lignin-polyether oligomers with enhanced hydroxyl group availability, improving solubility, molecular flexibility, and reactivity toward isocyanates during subsequent polyurethane formation. Increased hydroxyl content also promotes better crosslink density and phase dispersion within the resulting PU network. These LPs were then used to synthesize lignin-based polyurethane foams (LPUFs) by reacting them with hexamethylene diisocyanate (HDI), replacing 20–100 wt.% of conventional polyols (Figure 9) [67]. The inherent rigidity of lignin significantly enhanced the thermal and mechanical properties of the resulting foams. At 60% LP substitution, the foam exhibited a peak compressive stress of 86.80 kPa at 70% strain, which was approximately four times greater than the control polyurethane foam. This improvement was attributed to the reinforcing effect of lignin’s benzene ring structure. However, increasing LP content to 100% led to a decline in compressive stress (50.96 kPa), likely due to phase separation hindering optimal crosslinking. Additionally, the incorporation of lignin reduced the water contact angle and water absorption due to its hydrophobic nature and the reduced porosity of the foams. Biodegradation studies further demonstrated that LPUFs with 100% LP substitution degraded by 17.36% over 180 days, significantly improving compared to the 4.36% degradation observed in pure PUFs.

**Figure 10:**
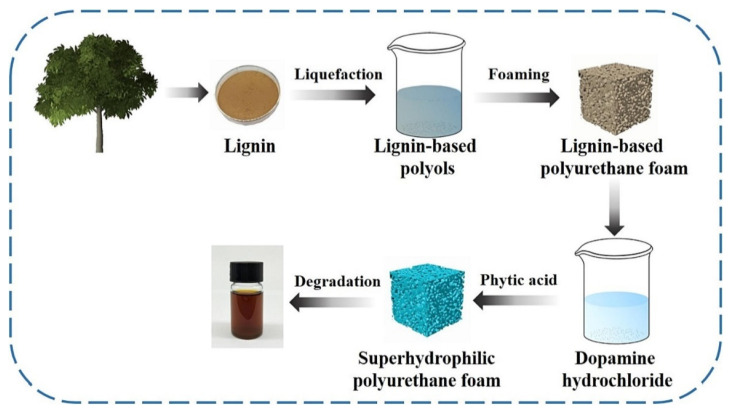
The preparation pathway of LPUF/PDA/PA and its degradation after usage in an alkali medium (after 5 h) [68]. Used with permission, copyright 2023 Elsevier.

Oily wastewater poses significant environmental challenges and is produced across various sectors, including oil refining, food processing, automotive maintenance, shipping, construction, metalworking, and oil extraction [75,76]. Foams are one of the ideal solutions to separate oil from water due to their variable porosity and lightweight properties. Chen and coworkers enzymatically hydrolyzed lignin and liquefied it into a lignin-based polyol. Then, polyols were reacted with diisocyanate to produce synthesized lignin-based polyurethane foam (LPUF) [68]. The LPUF was further modified by in situ polymerization of dopamine on the foam surface under weakly alkaline conditions to deposit polydopamine (PDA) particles, followed by surface modification using phytic acid (PA) to prepare LPUF/PDA/PA foams (Figure 10). LUPF was degraded entirely in an alkali medium within 5 h, which was attributed to the presence of lignin. The LPUF/PDA/PA exhibited excellent superhydrophilicity with a water contact angle of 0° and underwater superoleophobicity with oil contact angles exceeding 150°, achieving over 99% oil–water separation efficiency for various mixtures while maintaining high performance after ten cycles. Additionally, it demonstrated good adsorption capacities for methylene blue (67.1 mg/g), rhodamine B (96.1 mg/g), and copper sulfate (98.2 mg/g) and could completely degrade under weak alkaline conditions after use. However, some limitations of LPUFs include challenges in scalability, durability, complex fabrication processes, limited versatility, potential environmental degradation, and chemical leaching, demanding further research to enhance their performance and sustainability.

### 2.3. Non-Isocyanate Polyurethanes (NIPUs)

**Figure 11:**
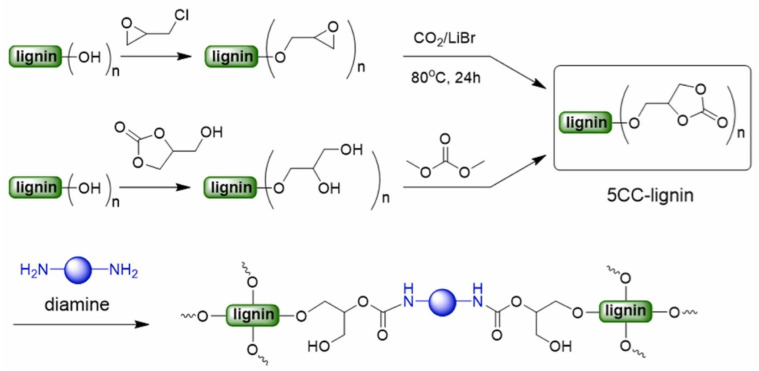
Non-isocyanate pathway to form polyurethanes from 5-membered cyclic carbonate functionalized lignin (5CC-lignin and amines.) [71]. Used with permission, copyright 2023 Elsevier.

Due to the high toxicity associated with isocyanates, there is a growing demand for developing and producing non-isocyanate polyurethanes (NIPUs) as a safer and more sustainable alternative to traditional PUs [77,78]. There are several methods to prepare NIPUs. Among them, aminolysis of cyclic carbonates (CCs) are more interesting because this approach involves the nucleophilic ring-opening reaction of CCs with amines, forming urethane linkages through a straightforward and non-toxic process. This process can be further enhanced by introducing five-membered cyclic carbonate groups (5CCs) into lignin or by depolymerizing lignin derivatives through glycidylation, followed by cycloaddition with CO_2_ or oxyalkylation with glycerol carbonate to incorporate adjacent hydroxyl groups (Figure 11) [69]. Subsequently, transesterification with dimethyl carbonate allows the production of a series of NIPUs via the polyaddition of cyclic carbonate-functionalized lignin and diamines.

**Figure 12:**
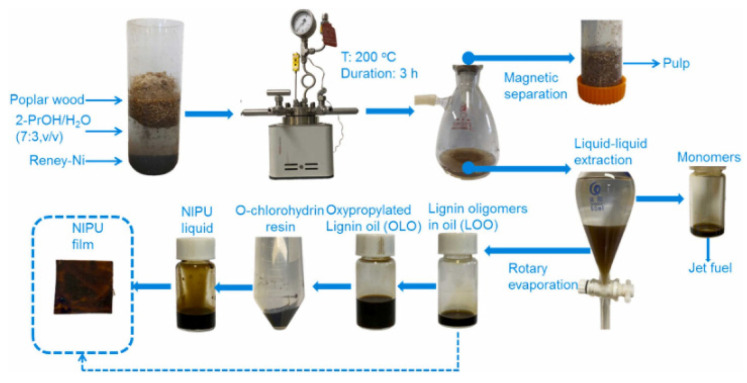
Preparation pathway of NIPU films from the lignin oligomers in the RCF oil [69]. Used with permission, copyright 2023 Elsevier.

**Figure 13:**
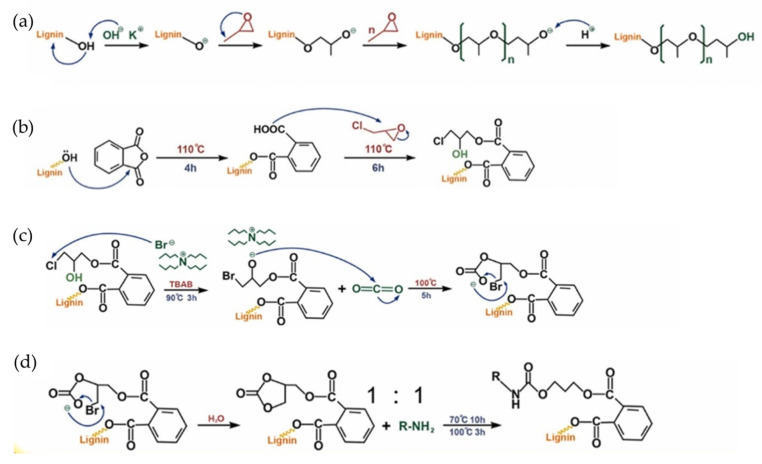
(**a**) The mechanism of oxypropylation reaction, (**b**) *o*-chlorohydrin resin formation (**c**) cyclic carbonation, and (**d**) NIPUs synthesis [69]. Used with permission, copyright 2023 Elsevier.

Yang and coworkers proposed using lignin oligomers derived from reductive catalytic fractionation (RCF oil) as a substitute for NIPU production (Figure 12) [69]. Sawdust was used as a renewable polyol source to produce lignin oligomers. These oligomers were oxypropylated and modified with phthalic anhydride and epichlorohydrin to form a cyclic carbonate (5CC) intermediate (Figure 13). This intermediate was reacted with various amines to synthesize NIPUs. The amino group content and molecular size of amines significantly impacted mechanical properties, though they had a minimal effect on thermostability. Additionally, at the molecular level, oxypropylation of lignin introduces flexible polyether side chains via grafting propylene oxide onto the hydroxyl groups. This significantly increases the number of aliphatic –OH groups, improving the reactivity of lignin with phthalic anhydride and epichlorohydrin for cyclic carbonate formation, and ultimately enhancing crosslinking density in the NIPU matrix. The flexible polyether chains contribute to improved toughness and tensile strength, while the rigid aromatic structure of lignin enhances thermal and UV resistance. The highest tensile strength (12.5 MPa) was achieved with polyether amine, while the highest Young’s modulus (88 MPa) was obtained with triethyltetraamine. The isophorone diamine-crosslinked NIPU exhibited the lowest mechanical properties. Moreover, the tensile properties, heat resistance, and UV resistance of the NIPUs were enhanced by the rigid benzene ring and phenolic groups in lignin. Although the mechanical properties of NIPUs were comparable to traditional PU, further research is needed to explore their durability and performance under real-world conditions. Additionally, scaling up this synthesis method may present challenges in terms of cost and efficiency.

**Figure 14:**
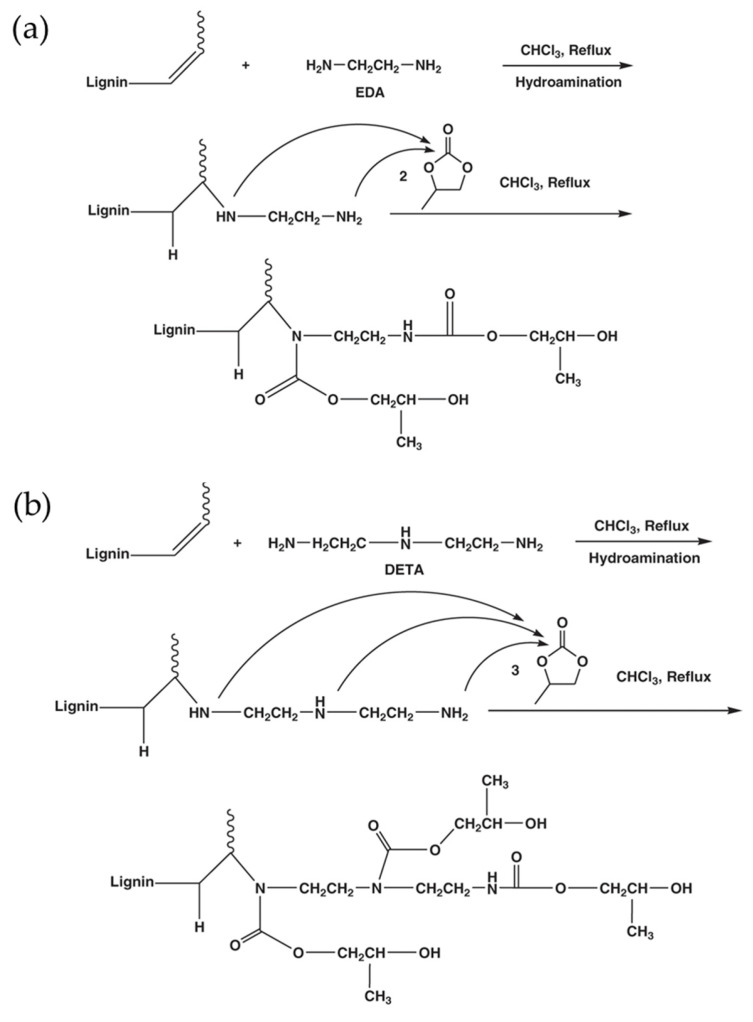
The reaction of (**a**) ethylene diamine (EDA) and (**b**) diethylenetriamine (DETA) with unsaturated regions of lignin, followed by the reaction of aminated lignin with propylene carbonate to produce lignin-based polyols [70]. Under a Creative Commons Attribution (CC BY) license (https://creativecommons.org/licenses/by/4.0/ accessed on 27 April 2025).

The previous method modified lignin with propylene carbonate to improve its solubility and reactivity. In the study by Najafi et al., lignin was initially modified with the amine, and the aminated product was next reacted with propylene carbonate (5CC) to enhance the hydroxy functionalities [70]. During the reaction, amination introduces nucleophilic amine groups onto the lignin backbone, which are then converted into hydroxyl groups through a ring-opening reaction with propylene carbonate. This two-step process significantly increases the hydroxyl number, thereby enhancing the lignin’s reactivity toward cyclic carbonate–amine systems in NIPU synthesis. The increased hydroxyl functionality also improves solubility and compatibility with other polyols. Furthermore, the reduced molecular weight of modified lignin may contribute to better dispersion and processability in polymer formulations. These structural changes explain the enhanced potential reactivity, although further studies are needed to correlate these modifications with final mechanical properties. In this work, lignin was extracted from raw liquor and then modified with diethylenetriamine (DETA) and ethylenediamine (EDA) to increase the number of functional groups and reactivity. The aminated lignin was further reacted with propylene carbonate using the Mannich reaction to prepare lignin-based polyols as an alternative to petroleum-based polyols to synthesize NIPU (Figure 14). This study did not address PU formation or the mechanical properties of the resulting polymers. However, evidence was provided through FT-IR analysis that the C-O bonds observed were due to urethane bonds in the modified lignin. GPC results revealed that lignin had been reduced to a lower molecular weight after modification, likely due to mechanical degradation during preparation. Additionally, the modified lignin-based polyol exhibited a much higher hydroxyl number (848–2220 mgKOH/g) than petroleum-based polyols, suggesting its strong reactivity potential. Therefore, further research is needed to apply this polyol in actual polyurethane production.

Although five-membered cyclic carbonates (5CCs) can be used to synthesize lignin-containing NIPUs, the process is complex and requires expensive metal-based catalysts due to the slow reaction between 5CCs and amines. Additionally, 5CCs have lower reactivity, leading to slower curing times, incomplete reactions, and reduced mechanical and thermal performance, such as weaker tensile strength and lower thermal stability. To improve these limitations, potential solutions include using more reactive six-membered cyclic carbonates (6CCs), modifying 5CCs to enhance their reactivity and mechanical properties, or blending 5CCs with 6CCs to create hybrid systems that optimize material performance by leveraging the strengths of both structures.

**Figure 15:**
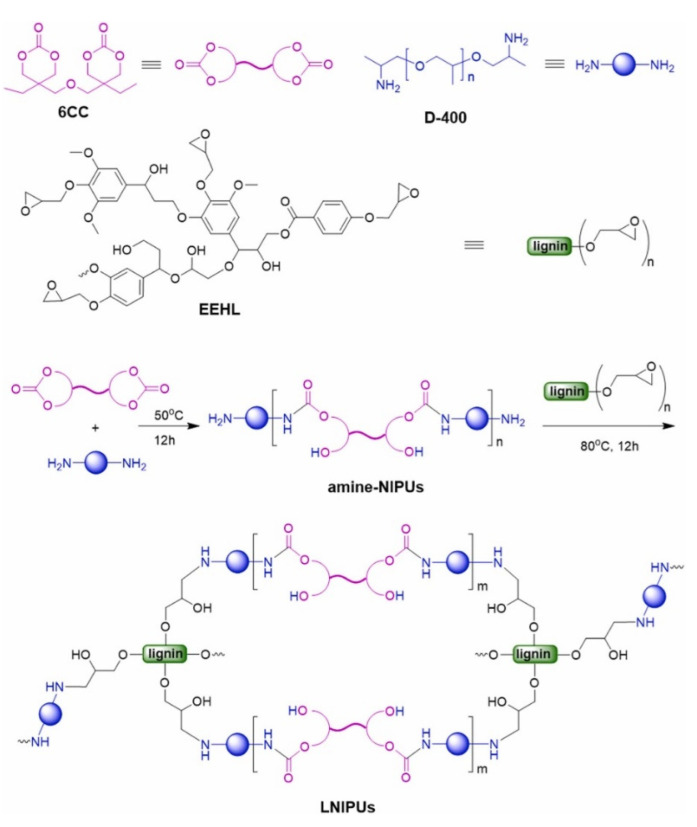
Illustration of the synthesis pathways for preparing LNIPUs [71]. Used with permission, copyright 2023 Elsevier.

Zhang and colleagues introduced a novel lignin/NIPU hybrid polymer (LNIPU) using bis(6-membered cyclic carbonate) (6CC) to address the limitations associated with 5CC. This example demonstrates how the structural rigidity of lignin influences toughness but limits self-healing, highlighting a critical trade-off in designing dynamic crosslinked networks with high-performance and reprocessability. Initially, amine-terminated non-isocyanate polyurethanes (amine-NIPUs) were synthesized from bis(6-membered cyclic carbonate) (6CC) and polyether amine (D-400) and then crosslinked with epoxidized enzymatic hydrolysis lignin (EEHL) at 0–50% epoxy group molar ratios to form LNIPU networks (Figure 15) [71]. Increasing lignin content in LNIPUs led to significant improvements in properties like storage modulus, glass transition temperature (*T*_g_), and crosslink density, and notable increases in tensile strength, Young’s modulus, and toughness. For instance, a material with 50% epoxy groups exhibited values of 20.25 MPa for tensile strength, 280.09 MPa for modulus, and 5.51 MJ/m^3^ for toughness, outperforming lignin-free versions. Adhesive strength also reached 24.5 MPa for bonding aluminum plates. Despite these gains, higher lignin content reduced elongation at break and self-healing efficiency, highlighting the need for optimized crosslinking strategies to improve overall material performance.

### 2.4. Waterborne Polyurethanes (WPUs)

Waterborne polyurethane (WPU) is an environmentally friendly polyurethane dispersed in water rather than organic solvents, thus reducing volatile organic compounds. Despite their significant advantages, WPUs encounter challenges related to mechanical performance, water resistance, drying times, and formulation complexity [72,73,74,79]. In response, Ng et al. developed a 3-aminopropyltriethoxy silane (APTES) functionalized kraft lignin-filled film to improve the reinforcing ability of WPUs [72]. Kraft lignin was functionalized by stirring it in a 2 wt.% APTES solution and subsequently mixed with WPU dispersion at varying proportions (0–2 wt.%) for comparison with unfunctionalized lignin. The films containing functionalized lignin demonstrated superior thermal stability compared to those with unfunctionalized lignin due to improved bonding. The optimal lignin loading was determined to be 0.5 wt.%, as higher concentrations resulted in agglomeration and reduced material properties. At this loading, functionalized lignin led to a 9% increase in ultimate tensile strength compared to pure WPU (from 8.05 MPa to 8.77 MPa), while unfunctionalized lignin yielded only a 0.3% improvement. Furthermore, films with functionalized lignin exhibited a lower Young’s modulus and higher elongation at break, indicating enhanced flexibility and improved deformation resistance. This research demonstrates the potential of lignin as a reinforcement filler to enhance mechanical properties and thermal stability in WPUs; however, further investigations are necessary to address issues related to water resistance, drying times, and formulation complexity.

**Figure 16:**
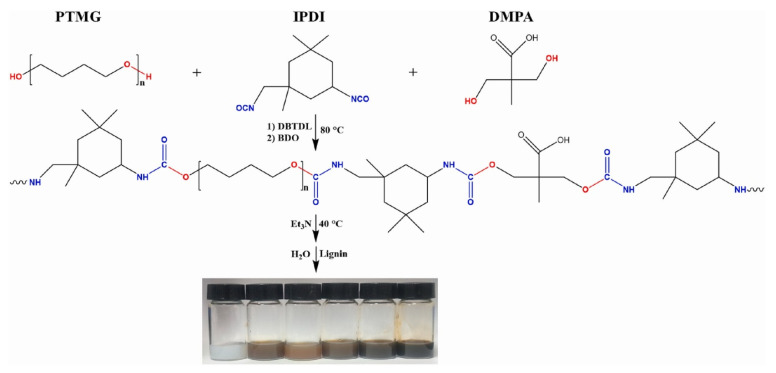
Preparation procedure of various lignin-based WPU emulsions [73]. Under a Creative Commons Attribution (CC BY) license (https://creativecommons.org/licenses/by/4.0/ accessed on 27 April 2025).

**Figure 17:**
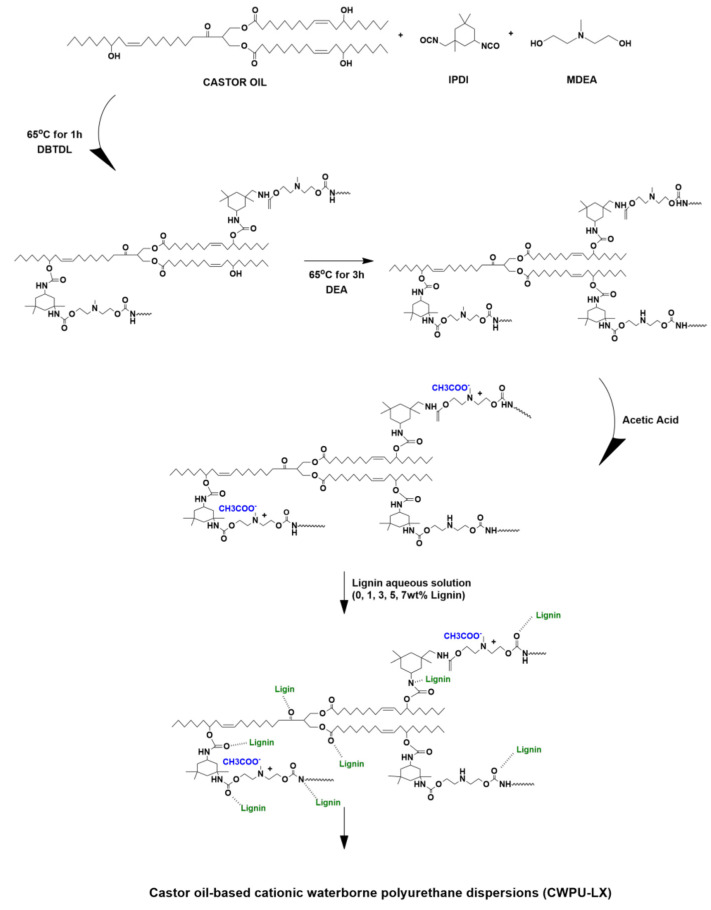
Schematic illustration of the preparation mechanism for castor oil-based cationic waterborne polyurethane incorporating lignin [74]. Under a Creative Commons Attribution (CC BY) license (https://creativecommons.org/licenses/by/4.0/ accessed on 27 April 2025).

In another study by Fan and co-workers, WPU emulsions were developed using lignin nanoparticles (LNPs) derived from enzymatic hydrolysis lignin (EHL) [73]. The nanoscale dispersion of LNPs enhances interfacial bonding with the polyurethane matrix, contributing to better mechanical reinforcement and UV resistance without sacrificing flexibility—demonstrating the utility of nano-lignin as a multifunctional additive. A series of WPU composites (Figure 16) was formulated by varying the LNP content from 0 to 5 wt.%. Notably, the incorporation of 5 wt.% LNPs led to significant enhancements in the overall performance of the composites, particularly in terms of durability and UV resistance when compared to neat WPU. Kim et al. introduced an innovative approach to synthesizing WPU by incorporating lignin into a castor oil-based cationic waterborne polyurethane system [74]. This strategy remarkably reduced the solvent required for polymer synthesis by 50%. Lignin was incorporated in varying amounts (0–7 wt.%) to produce WPU composites (Figure 17). The results demonstrated that lignin improved mechanical properties, thermal stability, solvent resistance, UV resistance, and antibacterial activity. The composite containing 7 wt.% lignin exhibited a significantly higher tensile strength (5.57 MPa) than pure WPU (1.10 MPa). This data demonstrates that lignin acts not only as a passive filler but as an active reinforcing phase, contributing to load-bearing via π–π stacking and hydrogen bonding with the castor oil-based polyurethane segments. Furthermore, this composite displayed remarkable ethanol resistance and achieved 97.27% antibacterial activity against *Escherichia coli* (*E. coli*), compared to only 1.58% for the pure WPU.

## 3. Lignin as a Precursor in Thermosets and Crosslinked Polymer Networks

### 3.1. Epoxy Resins

**Scheme 2:**
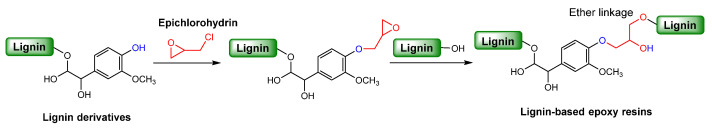
Synthesis pathways of lignin-based epoxy thermosets.

Epoxy resins are reactive pre-polymers that form crosslinked thermosets through curing reactions with various co-reactants, and they are widely used in applications such as coatings, adhesives, electrical insulation, and reinforced composites. Among them, the most commonly used epoxy resin reactant is diglycidyl ether of bisphenol A (DGEBA), which is synthesized from fossil-derived bisphenol A (BPA) [80,81]. However, the extensive use of BPA has raised significant environmental and health concerns, prompting the search for sustainable alternatives. Lignin is a promising substitute for BPA in the development of bio-based epoxy resins. Functionalization of lignin through glycidylation, typically via reaction with epichlorohydrin, introduces epoxy groups that enable the formation of crosslinked thermoset networks with ether linkages upon curing (Scheme 2). This section will discuss the use of lignin as a renewable feedstock to enhance the performance of epoxy resins while reducing dependence on petroleum-based polymers. Lignin types, modification methods, applications, and key properties for lignin-derived resins are summarized in Table 2.

**Figure 18:**
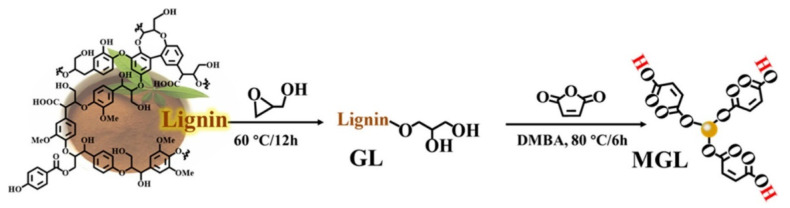
Lignin modification to synthesize glycidylated lignin (GL) and multicarboxyl lignin (MGL) [82]. Used with permission, copyright 2023 American Chemical Society.

**Figure 19:**
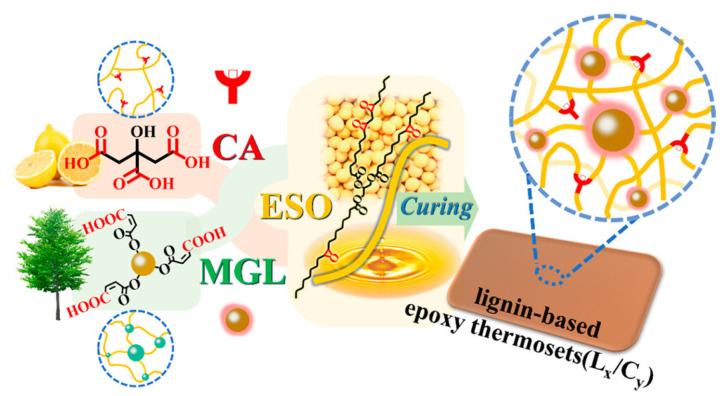
Preparation procedure for lignin-based epoxy thermosets (L_x_/C_y_) [82]. Used with permission, copyright 2023 American Chemical Society.

Zhou et al. developed fully bio-based epoxy thermosets using modified lignin (MGL) as a rigid crosslinker, combined with citric acid (CA) and epoxidized soybean oil (ESO) [82]. To enhance solubility, reactivity, and compatibility, lignin was chemically modified through glycidylation with glycidol to produce glycidylated lignin (GL), followed by maleation with maleic anhydride to introduce carboxyl groups, yielding MGL (Figure 18). In here, the glycidylation introduces epoxide groups that enhance compatibility and reactivity with the epoxidized soybean oil, promoting denser crosslinking. Maleation introduces carboxyl groups, which further increase hydrogen bonding and crosslinking potential with citric acid, thereby enhancing mechanical strength and rigidity. The thermosets were prepared by varying the MGL-COOH to CA-COOH ratios (L_10_/C_0_, L_8_/C_2_, L_5_/C_5_, L_0_/C_10_) while maintaining a fixed epoxy-to-carboxyl molar ratio of 1:0.8 (Figure 19). Dynamic mechanical analysis (DMA) revealed an increase in glass transition temperature (*T*_g_) from 17.1 °C (L_0_/C_10_) to 96.5 °C (L_10_/C_0_) with higher MGL content, indicating greater rigidity and crosslink density. Thermogravimetric analysis showed similar degradation temperatures at 5% weight loss (*T*_5%_) of 322–328 °C in nitrogen and 308–312 °C in air across all lignin-modified compositions. Mechanical testing demonstrated a rise in tensile strength from 0.6 MPa (L_0_/C_10_) to 33.2 MPa (L_8_/C_2_) and an increase in tensile modulus from 2.0 MPa to 480.5 MPa with higher MGL content. The highest elongation at break (55.1%) was observed for L_5_/C_5_, while L_10_/C_0_ exhibited the lowest (9.3%). These thermosets displayed superior tensile strength compared to previously reported lignin-based systems and showed degradability in alkaline conditions due to ester bond hydrolysis. This result illustrates how glycidylation followed by maleation significantly increases the crosslinking efficiency and mechanical rigidity of lignin-epoxy networks, while preserving green degradability pathways, thereby balancing performance and sustainability.

**Figure 20:**
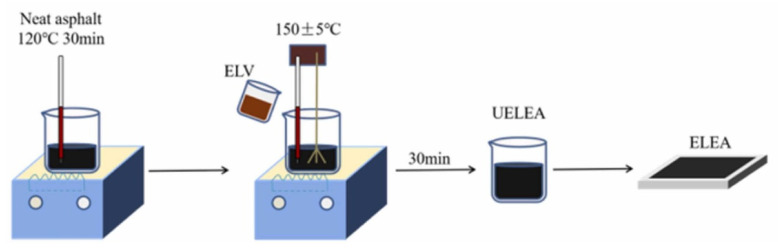
Illustration for the synthesis of lignin-based epoxy asphalt systems [83]. Used with permission, copyright 2023 Elsevier.

Conventional epoxy asphalt is a thermosetting material that is difficult to recycle [96]. Song et al. developed a lignin-based vitrimer system (ELV) to replace conventional epoxy asphalt, incorporating dynamic covalent ester-exchange bonds for recyclability [83]. Enzymatic lignin (EL) was reacted with epichlorohydrin (ECH) under alkaline conditions to obtain lignin epoxy resin (LER). EL was esterified with maleic anhydride (MA) to obtain carboxylated lignin (EL-COOH). The lignin-based vitrimer system (ELV) was prepared by the Zn^2+^ catalyzed reaction of LER and EL-COOH in the presence of polyethylene glycol 400 (PEG 400). ELV (10–30 wt.%) was then introduced into base asphalt to produce different lignin-based epoxy asphalt systems (ELEA, Figure 20). ELEA exhibited improved high-temperature stability, with a higher softening point as ELV content increased. The glass transition temperature (*T*_g_) remained stable (8–10 °C), ensuring good low-temperature performance. ELEA-20 (20 wt.% of ELV) showed comparable thermal stability to base asphalt (initial decomposition at 287 °C) and higher maximum decomposition temperature (470 °C vs. 454 °C). ELEA-20 met tensile strength (>1.5 MPa) and elongation at break (>200%) requirements for steel bridge deck pavement. Increasing ELV content enhanced tensile strength but reduced ductility. The dynamic covalent bonds in ELEA enable high-temperature restructuring, offering potential for recovery and recycling.

**Figure 21:**
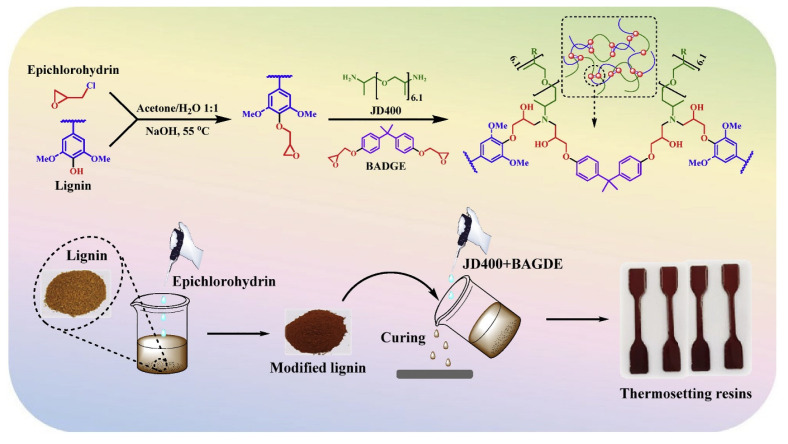
Schematic illustration of epoxidation using modified lignin [84]. Used with permission, copyright 2023 Elsevier.

Zou and coworkers developed low-carbon, bio-based epoxy thermosetting materials using industrial alkali lignin [84]. Their approach highlights the advantage of fractionation for controlling molecular weight distribution, which directly impacts processability, epoxy functionalization efficiency, and mechanical consistency in the final resin network. To achieve better uniformity and lower molecular weight, the lignin was fractionated with acetone (F_ASL) and subsequently epoxidized with epichlorohydrin, introducing epoxy groups onto the lignin backbone (F_ASLM). This modification enhanced lignin’s reactivity, enabling partial substitution of petroleum-based bisphenol A diglycidyl ether (BADGE) in epoxy resin formulations. The thermosetting resin was prepared by blending F_ASLM (5–10 wt.%) and BADGE in varying proportions, with polyetheramine (JD400) as the crosslinking agent (Figure 21). Thermal stability analysis revealed a significant improvement, with the degradation temperature (*T*_5%_) increasing from 200–230 °C for unmodified lignin to 270–290 °C for epoxidized lignin. The cured resins containing 5% and 10% F_ASLM demonstrated enhanced thermal properties, with *T*_5%_ reaching 315 °C and 289 °C, respectively, compared to pure BADGE. Mechanical properties also improved with lignin incorporation. The 5% F_ASLM resin exhibited a tensile strength of 4.6 MPa and an elongation at the break of 315.5%, outperforming pure BADGE (4.19 MPa, 161.16%). At 10% lignin content, tensile strength increased to 5.42 MPa, with elongation at break reaching 255.32%. However, at lignin concentrations above 10%, agglomeration issues were observed, and higher lignin content (above 20%) necessitated elevated curing temperatures, limiting its substitution potential. While epoxidation improved lignin’s compatibility with BADGE, further optimization is needed to enhance interface compatibility and maximize lignin incorporation without compromising material properties.

**Figure 22:**
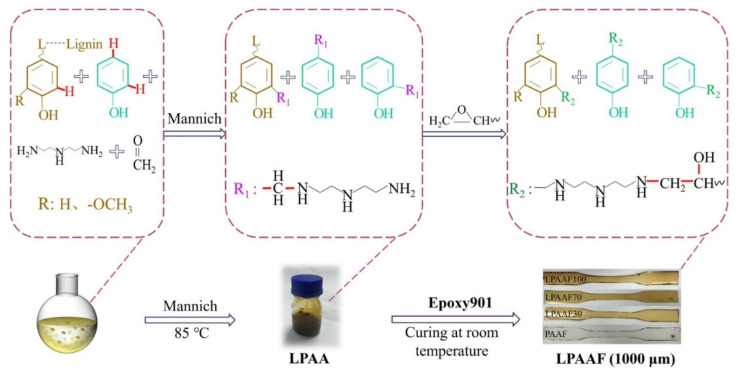
Preparation of lignin-based phenolic amine (LPAA) and epoxy resin (LPAAF) [85]. Used with permission, copyright 2023 Elsevier.

Li and coworkers utilized black liquor lignin, a byproduct of the pulping industry, to develop lignin-based epoxy composite films [85]. Their strategy exemplifies a cost-effective valorization route, converting underutilized industrial byproducts into functional curing agents. The resulting composites reveal how aromatic rigidity and functional group density of modified lignin can be tuned to reinforce resin network architecture. The black lignin was modified via a Mannich reaction with phenol, formaldehyde, and diethylenetriamine to produce lignin-based phenolic amine (LPAA), which served as a curing agent for bisphenol A-based epoxy resin (E901). Phenolation introduces additional phenolic hydroxyl groups onto the lignin backbone, increasing both hydrogen-bonding capacity and reactive sites for subsequent Mannich-type amine functionalization. This increases the crosslinking density and intermolecular interactions within the epoxy network. Furthermore, the incorporation of phenol increases the aromatic content of lignin, resulting in a stiffer and more thermally stable network. LPAA was synthesized with varying mass ratios of black liquor lignin to phenol: 30:70, 70:30, and 100:0. The resulting LPAA and E901 were then combined in a 1:0.1 mass ratio to form the composite film (LPAAF, Figure 22). Thermal analysis showed an improvement in the degradation temperature at 5% weight loss (*T*_5%_) compared to the control sample without lignin, with increasing black liquor lignin content enhancing thermal stability. The tensile strength of LPAAF increased with lignin content, reaching a maximum of 30 MPa for LPAAF70 (70 wt.% black liquor lignin in LPAA). However, elongation at break decreased due to the rigid three-dimensional lignin network. Inorganic chemicals from black liquor lignin were well dispersed in the films, and future studies are required to assess the long-term stability and durability of the composites.

**Figure 23:**
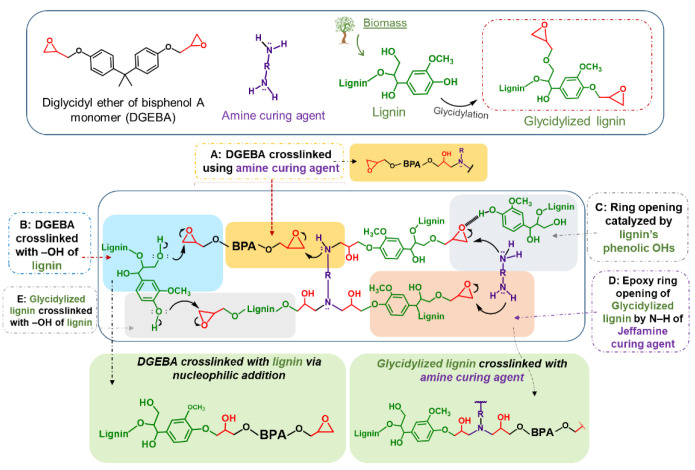
Proposed mechanism for crosslinking of lignin/DGEBA/diamine to prepare epoxy resins [86]. Used with permission, copyright 2023 Elsevier.

Pappa et al. explored the integration of kraft lignin (KL) into DGEBA-based epoxy resin systems to enhance sustainability and performance. Their work illustrates the importance of particle size and chemical compatibility. Nano-lignin incorporation preserved transparency and improved mechanical integrity, emphasizing that lignin processing form—micro vs. nano—has direct performance implications. KL was employed in various forms: as a direct additive, a partial replacement for the amine curing agent, and in a chemically modified form via glycosylation (GKL, Figure 23) [86]. Additionally, KL was used at both micro- and nanoscales, with nano-lignin (NLH) yielding transparent composites with notable improvements in thermal and mechanical properties. Glycidylated lignin enabled high substitution levels of bio-based epoxy content (up to 38 wt.%) while maintaining or enhancing composite performance. At 3 wt.% KL loading, tensile strength increased by 25% without compromising strain at break. Optimal improvements in tensile stress (3.1%), strain (35.2%), and stiffness (28.9%) were observed at 3 wt.% KL, while higher loadings (up to 9 wt.%) showed a trade-off with reduced strain but retained strength. Ball-milled KL (BMKL) composites showed moderate property enhancements even at 6 wt.% loading. Notably, NLH-based composites sustained or improved mechanical properties up to 30 wt.% loading, showing better stress, strain, and stiffness compared to the pristine polymer. GKL allowed substitution of up to 21 wt.% DGEBA (16.6 wt.% GKL content) with significant improvements in all mechanical metrics. These results highlight KL’s versatility and effectiveness in enhancing epoxy systems, particularly when chemically modified or nanoscaled, making it a strong candidate for greener, high-performance thermoset composites.

**Figure 24:**
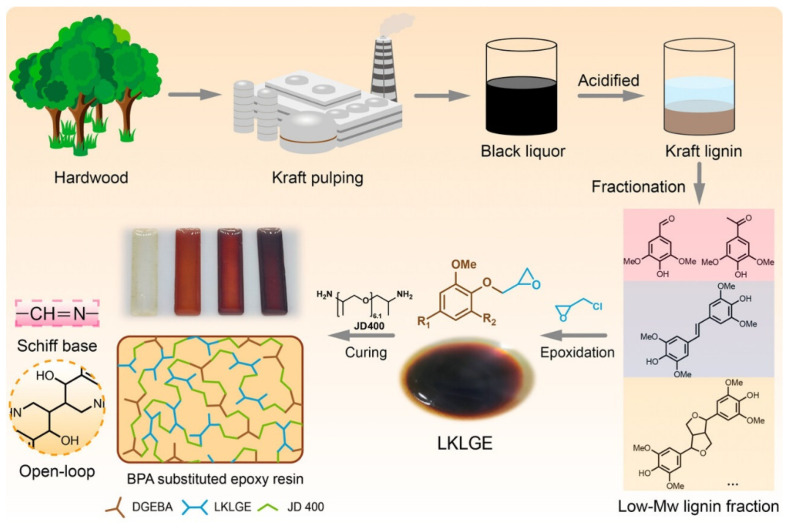
Preparation procedure of lignin-based epoxy resin using low-molecular-weight lignin glycidyl ether (LKLGE) [87]. Used with permission, copyright 2023 American Chemical Society.

**Figure 25:**
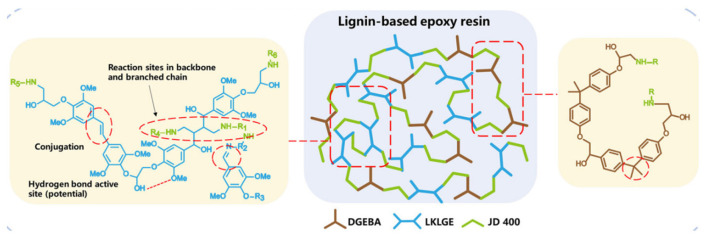
Toughening mechanisms in lignin-based epoxy resins and their impact on mechanical properties [87]. Used with permission, copyright 2023 American Chemical Society.

Xue and coworkers developed a lignin-based substitute for bisphenol A (BPA) for producing epoxy resin [87]. Kraft lignin was fractionated to produce low-molecular-weight (low-Mw) lignin, mainly consisting of syringaresinol (0–60 wt.%), to synthesize epoxy resins (Figure 24). The prepared epoxy resins with a 20% substitute amount exhibited the optimum properties. Thermal stability increased by 48%, and mechanical properties were significantly enhanced, with elongation improved by 239% and tensile stress by 144% compared to the BPA-based epoxy resin. The improved performance is attributed to the unique structural features of the lignin components: furan ring-opening of syringaresinol forming a flexible 3D crosslinked network, retention of conjugated C=C bonds in stilbene enhancing stress transfer, and Schiff base formation from aldoketones contributing to chain extension and flexibility (Figure 25).

**Figure 26:**
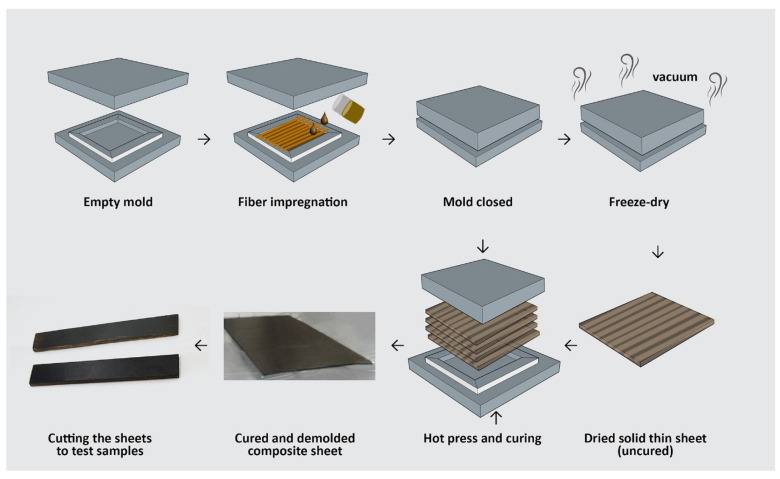
Schematic representation of the fabrication process for unidirectional, fully bio-based lignin epoxy/bamboo composites [88]. Under a Creative Commons Attribution (CC BY-NC-ND 4.0) license (https://creativecommons.org/licenses/by-nc-nd/4.0/).

In another study, Bagheri and colleagues developed a bio-based epoxy resin to fully replace BPA [88]. In this approach, kraft lignin was epoxidized using a bio-derived epichlorohydrin (ECH) sourced from glycerol. The resulting resin was then used to impregnate unidirectional bamboo fibers, forming composite sheets for the fabrication of a water-based epoxy resin (Figure 26). During the reaction, epoxidation of kraft lignin introduces reactive epoxide rings onto the lignin structure, enhancing its ability to form covalent bonds during curing, which improves the network formation with curing agents and leads to better load transfer within the composite. The aromatic-rich structure of lignin promotes interfacial compatibility with bamboo fibers due to π–π and hydrogen-bonding interactions. When comparing the mechanical properties, the intial flexural strength of the lignin-based composite was lower than that of commercial epoxy, and the addition of 25 wt.% bamboo fibers significantly improved the flexural strength due to the strong interfacial bonding and natural compatibility between lignin and the bamboo fibers. Furthermore, the incorporation of fibers led to a notable increase in the flexural modulus, reinforcing the potential of this system to replace petroleum-based polymers.

**Figure 27:**
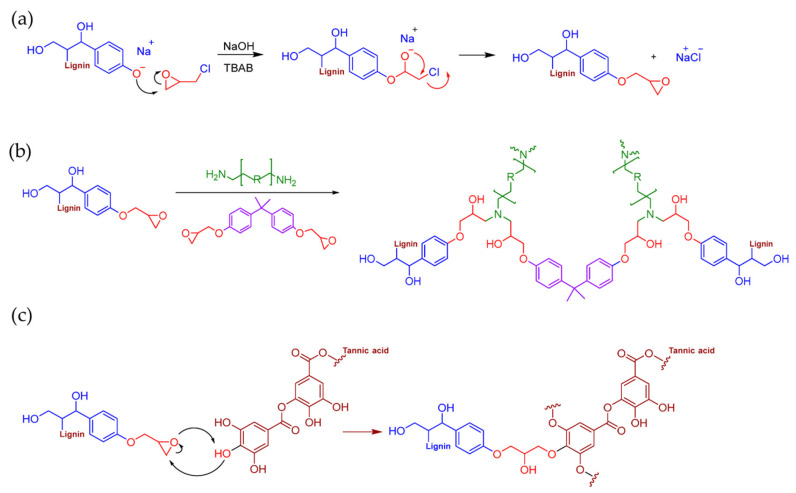
Proposed mechanism for (**a**) lignin epoxidation, (**b**) crosslinking of DGEBA, epoxy lignin, and amine hardener, and (**c**) crosslinking of epoxy lignin with tannic acid during curing [89]. Under a Creative Commons Attribution (CC BY) license (https://creativecommons.org/licenses/by/4.0/ accessed on 27 April 2025).

Gavrilović-Grmuša et al. developed a bio-based epoxy adhesive for wood applications by incorporating lignin and tannic acid as reactive components [89]. Specifically, kraft lignin was chemically modified through epoxidation to partially substitute diglycidyl ether of bisphenol A (DGEBA), a conventional petroleum-based epoxy monomer (Figure 27). The modified resin formulations included lignin in concentrations ranging from 5 to 15 wt.%. Notably, the lower lignin content (5 wt.%) yielded the highest tensile shear strength (10.87 MPa), comparable to that of the unmodified resin. However, as the proportion of lignin increased, a gradual decline in adhesive strength was observed. This reduction was attributed to excess lignin’s limited dispersion and reactivity within the epoxy matrix. The structural similarity and chemical compatibility between lignin and tannic acid enhanced the mixture’s homogeneity, thereby reducing lignin agglomeration and improving its interaction with the epoxy network.

**Figure 28:**
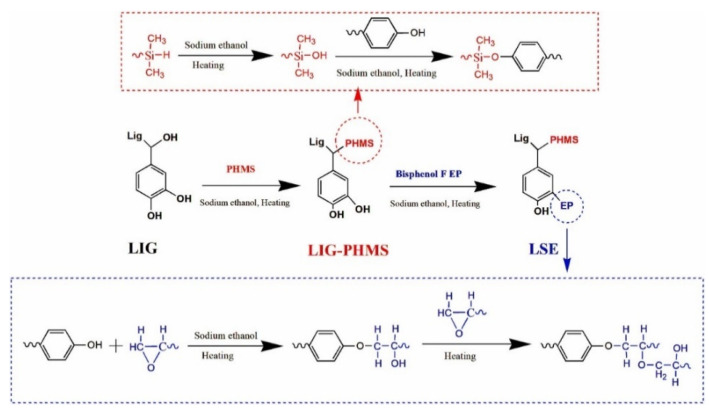
Synthesis of lignin-based silicone-modified epoxy resin [90]. Used with permission, copyright 2024 Elsevier.

Li et al. developed a lignin-based silicone-modified epoxy resin to improve the mechanical performance of epoxy resins, which are widely used in electronic packaging. The synthesis involved two main steps (Figure 28): first, flexible silicone chains were grafted onto lignin (0–9 wt.%) through a condensation reaction between the Si–H groups of polyhydromethylsiloxane (PHMS) and the hydroxyl groups of lignin. In the second step, the modified lignin was incorporated into the epoxy matrix via additional condensation between the lignin hydroxyl groups and the epoxy resin [90]. Optimal mechanical performance was achieved at 3 wt.% lignin loading, where impact strength increased by 16.25%, tensile strength by 25.81%, bending modulus by 16.21%, and bending strength by 42.43% relative to neat epoxy resin. Moreover, elongation at break improved progressively with increasing lignin content, with a 37.86% enhancement observed at 3 wt.% lignin addition. Although higher lignin content reduced glass transition temperature and crosslink density, the modified resins still exhibited a desirable combination of toughness and strength.

### 3.2. Phenol-Formaldehyde Resins

**Scheme 3:**
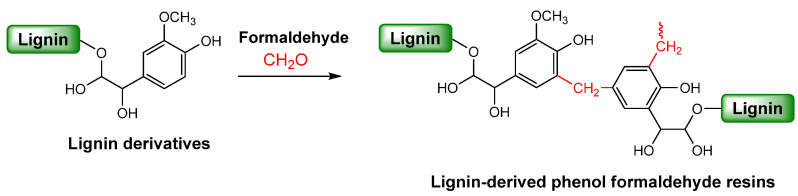
Synthesis of lignin-modified phenol-formaldehyde resins.

Phenol-formaldehyde (PF) resins are widely utilized in wood adhesives, molding compounds, and insulation materials due to their excellent mechanical strength, water resistance, thermal stability, and flame retardancy [97,98]. Traditionally, these resins are synthesized from petroleum-derived phenol and formaldehyde. However, the use of these chemicals raises significant environmental and health concerns due to the toxicity of both phenol and formaldehyde. In response, lignin as a low-cost, renewable aromatic polymer has garnered significant attention as a sustainable alternative. Lignin’s complex structure contains reactive phenolic and aliphatic hydroxyl groups, aldehydes, and carboxylic functionalities, which can participate in PF resin synthesis. Notably, the phenolic hydroxyl groups in lignin can undergo condensation reactions with formaldehyde, forming methylene bridges (–CH_2_–) between aromatic units, thereby mimicking the structure of conventional PF networks (Scheme 3). This reactivity makes lignin a promising substitute for phenol in the development of eco-friendly phenolic resins. This section will highlight the role of lignin in various lignin-derived PF resin formulations, emphasizing its potential to reduce environmental impact while maintaining or enhancing resin performance.

Rodrigues et al. investigated the development of lignin-based phenol-formaldehyde (LPF) resins using fractionated kraft lignin (KL) with lower molar mass fractions [91]. This example demonstrates how lignin fractionation is not merely a purification strategy, but a functional design tool that enhances solubility and reactivity, ultimately improving adhesive bond strength and resin homogeneity. The lignin was extracted from Eucalyptus urograndis wood using ethyl acetate, resulting in both soluble and insoluble fractions. The study explored the effect of substituting 25% and 50% of phenol with fractionated lignin to produce eco-friendly LPF resins. The fractionation process notably increased the total hydroxyl content and solubility of lignin, which are critical parameters for enhancing resin performance. Thermal analysis demonstrated that the inclusion of lignin improved the thermal stability and increased the glass transition temperature (*T*_g_) of the resins. Among the various formulations, resins containing 25% soluble lignin fractions, cured at 150 °C, exhibited adhesive properties comparable to pure phenol-formaldehyde (PF) resins without lignin substitution. Furthermore, the adhesive bond strength of the soluble fraction LPF resins was significantly higher than that of the insoluble fraction LPF resins and non-fractionated KL-based resins. This superior performance highlights the beneficial role of lignin fractionation in optimizing the properties of bio-based resins. Overall, the study successfully demonstrated that a small percentage of soluble lignin fractions can effectively replace phenol in commercial PF resins, contributing to the synthesis of environmentally friendly adhesive systems.

**Figure 29:**
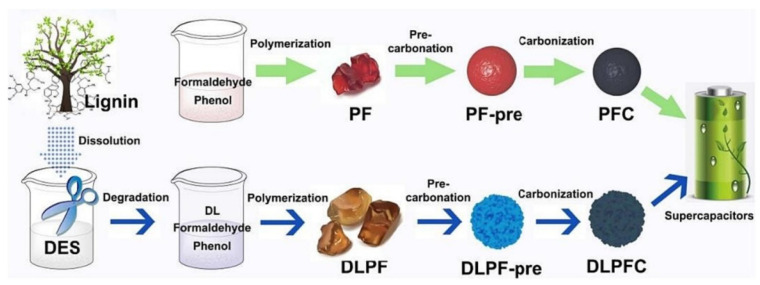
Schematic representation of lignin-based phenolic resin (DLPFC) preparation process and phenolic resin carbon (PFC) [92]. Used with permission, copyright 2023 Elsevier.

Li and coworkers deployed alkaline lignin with a deep eutectic solvent (DES) composed of *p*-toluenesulfonic acid and choline to prepare phenolic resin and porous carbon [92]. The depolymerized lignin was co-polymerized with phenol and formaldehyde to produce a lignin-based phenolic resin (DLPFC, Figure 29). A pure phenolic resin carbon (PFC) was also prepared similarly without lignin for comparison. The electrochemical performances of the prepared materials were obtained as supercapacitor electrodes. After the incorporation of lignin, DLPFC materials showed higher specific surface area, higher electrical conductivity, and better graphitization compared to pure resins. As a supercapacitor electrode, DLPFC exhibited higher specific capacitance (245.8 F/g at 0.25 A/g) than PFC (159.7 F/g) in a three-electrode system and had excellent cycling stability, retaining 97.6% capacitance after 10,000 cycles. In a two-electrode system, the DLPFC achieved an energy density of 3.9 Wh/kg at a power density of 125 W/kg, comparable to commercially available batteries.

Galsino and coworkers used kraft lignin as a substitute for phenol in synthesizing phenol-formaldehyde (PF) resins [93]. Lignin was modified through a phenolation process to increase the reactivity of lignin and substituted phenol with varying proportions of phenolated kraft lignin (10–50 wt.%) to produce lignin–phenol-formaldehyde (LPF) resins. The LPF resins with phenol substitution above 20% (LPF30 and LPF20) showed superior shear strength compared to the control PF resin, and the highest shear strength was observed for the LPF30 resin (3.41 MPa). In TGA, the addition of kraft lignin reduced the thermal stability of the resins, resulting in lower decomposition temperatures and smaller amounts of carbonaceous residues. The curing of lignin-based resins occurred at lower temperatures with higher enthalpies than the PF resin. Overall, the study demonstrates the potential of using kraft lignin as a sustainable substitute for phenol (up to 30% substitution) in phenol-formaldehyde resins for structural composite applications, highlighting the need for further optimization and investigation.

**Figure 30:**
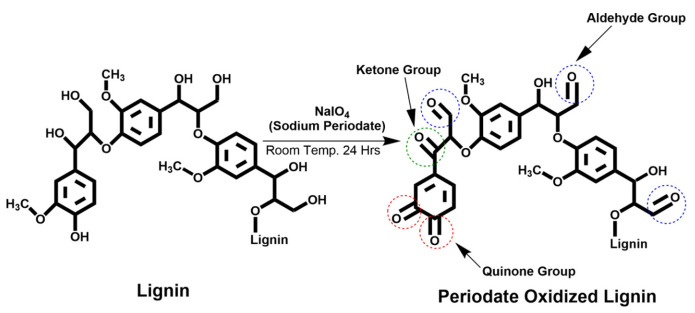
Schematic representation of the periodate oxidation of softwood lignin, targeting aromatic and aliphatic hydroxyl groups [94]. Used with permission, copyright 2023 American Chemical Society.

Bansode and coworkers explored the use of lignin as a substitute for phenol and formaldehyde in the preparation of phenol-formaldehyde (PF) wood adhesive resins [94]. Kraft lignin, derived from pine trees, was chemically modified using sodium periodate oxidation to introduce aldehyde functionalities (Figure 30). The oxidized lignin was then employed to partially or fully replace formaldehyde in synthesizing bio-based novolac PF (BNPF) resins. In the study, unmodified lignin was used to replace phenol, while oxidized lignin served as a formaldehyde replacement to produce BNPF resins. The tensile shear strengths of the resulting BNPFs were compared to those of commercially available novolac PF resins. Results showed that lignin-based PF resins exhibited superior dry adhesion strength compared to commercial PF resins. However, the wet adhesion strength of lignin-based resins was lower, likely due to the hydrophilic nature of lignin. Additionally, the curing temperature of the BNPF resins was reduced when formaldehyde was replaced with oxidized lignin. This reduction in curing temperature is attributed to the higher reactivity of periodate-oxidized lignin compared to formaldehyde when reacting with phenol or lignin.

**Figure 31:**
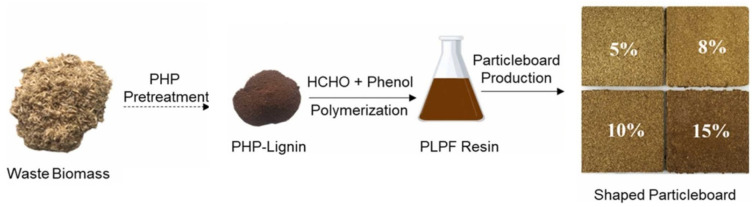
Synthesis of phosphoric acid and hydrogen peroxide pretreated lignin–phenol-formaldehyde resins (PLPF) and particleboards [95]. Used with permission, copyright 2024 Elsevier.

Zhao et al. developed lignin–phenol-formaldehyde (LPF) resins using unmodified lignin derived from phosphoric acid and hydrogen peroxide (PHP) pretreated biomass waste and evaluated their performance as wood adhesives [95]. The PHP-lignin-based LPF (PLPF, Figure 31) resins were compared with those synthesized from conventional alkali lignin (ALPF). Particleboards incorporating 5–15 wt.% LPF resin exhibited good dimensional stability and solvent resistance. However, the presence of resin adhesives hindered enzymatic hydrolysis, reducing cellulose-to-glucose conversion to 18.3%. An alkali pretreatment step significantly improved glucose conversion up to 57.8%, offering a promising detoxification strategy for recycling lignocellulosic composites. In adhesive performance, the bond strength of lignin-derived resins met the requirements for first-grade plywood (≥0.7 MPa), although the ultimate bonding strength of PLPF (2.16 MPa) and ALPF (1.59 MPa) was lower than that of pure PF resin (3.09 MPa). This reduction was attributed to the lower reactivity of lignin due to fewer reactive sites, higher molecular weight, and steric hindrance. Notably, PLPF outperformed ALPF, likely due to the higher hydroxyl content of PHP-lignin enhancing its reactivity with formaldehyde.

## 4. Polylactic Acid (PLA) Composites 

**Scheme 4:**
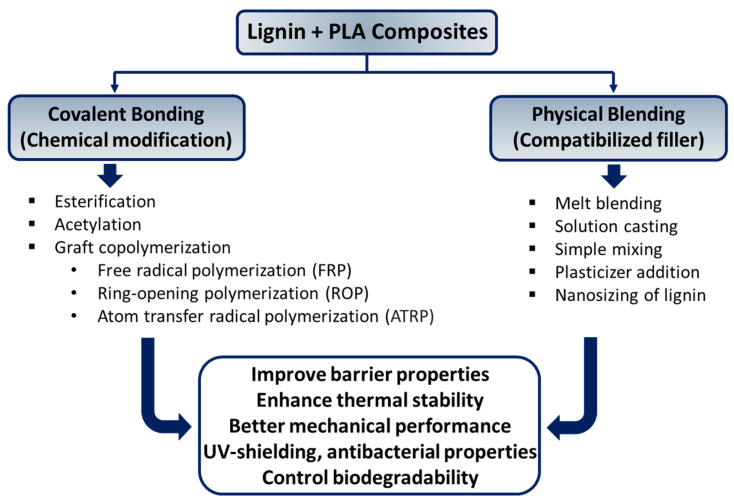
Strategies for incorporating lignin into polylactic acid (PLA) systems.

Poly(lactic acid) (PLA) is a versatile aliphatic linear thermoplastic biodegradable polymer derived from fully renewable sources such as wheat, corn, rice, and sweet potatoes. It offers numerous advantages, including renewability, sustainability, biocompatibility, and compostability, making it an attractive candidate for eco-friendly materials. PLA production involves low energy consumption and generates minimal greenhouse gas emissions, further enhancing its appeal for various applications, including 3D printing [99,100]. However, PLA also exhibits several drawbacks, such as poor gas and water barrier properties, limited toughness, low glass transition temperature, hydrophilicity, and challenges with dispersion when blended with other components [101]. These limitations hinder its widespread use in commercial applications. PLA is often blended with natural additives like lignin and fibers to address these issues, to enhance its thermal stability, water barrier performance, crystallization behavior, mechanical strength, antimicrobial properties, and degradability (Scheme 4). This section explores recent advancements in the development of biodegradable composites, focusing specifically on incorporating lignin into PLA to improve its performance characteristics (Table 3).

Shi and coworkers developed wholly bio-based composites by grafting lignin with lauryl methacrylate (LMA), a renewable plant-based fatty acid monomer, through free-radical polymerization to produce lignin graft copolymers (LG-*g*-PLMA) [102]. These copolymers (0–20 wt.% of LG-*g*-PLMA) were subsequently blended with polylactic acid (PLA) to create the composites. The long hydrophobic side chain of LMA was designed to improve the hydrophobicity, flexibility, and compatibility of lignin with PLA, while lignin was anticipated to contribute UV barrier properties to the composites. The incorporation of LG-*g*-PLMA significantly enhanced the crystallinity of PLA, increasing from 6.42% to 17.46%. When the LG-*g*-PLMA loading was below 9%, the thermal stability of the composites showed a slight improvement compared to pure PLA. However, the glass transition temperature (*T*_g_) of the composites decreased with increasing LG-*g*-PLMA content. At a 5% LG-*g*-PLMA loading, the composites demonstrated a 42% increase in elongation at break and a 36% increase in tensile toughness compared to pure PLA. While the tensile strength of the composites declined from 55.6 MPa (pure PLA) to 26.5 MPa at 20% LG-*g*-PLMA loading, the composites exhibited a strong UV barrier effect, nearly completely blocking UV light at low LG-*g*-PLMA concentrations. The successful grafting of LMA onto the lignin via free-radical polymerization enhanced both the hydrophobicity and thermal stability of lignin. This modification also improved its compatibility with PLA and strengthened interfacial adhesion. As a result, the composites exhibited improved mechanical properties even at low LG-g-PLMA loading.

**Figure 32:**
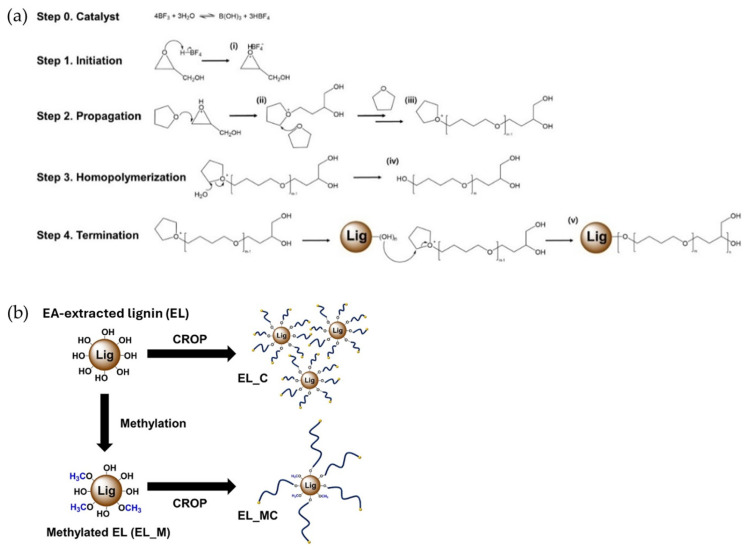
(**a**) The proposed mechanism of cationic ring-opening polymerization (CROP) reaction and (**b**) illustration for the preparation of EL_C and EL_MC [103]. Used with permission, copyright 2023 Elsevier.

Kim et al. addressed the inherent brittleness and poor UV resistance of PLA by incorporating chemically modified lignin, leveraging cationic ring-opening polymerization (CROP) techniques to enhance material performance [103]. The modification involved ethyl acetate-extracted lignin (EL) subjected to CROP using tetrahydrofuran (THF) and glycidol (GLY) in the presence of a boron trifluoride (BF_3_) catalyst, resulting in EL_C and EL_MC (methylated EL) variants (Figure 32). The methylation of EL was specifically designed to regulate the hydroxyl content, optimizing compatibility with the PLA matrix. The CROP reaction was carried out to promote homopolymerization under starvation of the initiator (oxirane), leading to the formation of long polyether chains grafted onto the lignin core. The introduction of these polyether chains aimed to improve the compatibility and interfacial adhesion between the hydrophobic lignin and the non-polar PLA matrix. To fabricate lignin/PLA films, EL, EL_C, and EL_MC were individually dissolved in chloroform along with PLA, followed by solvent casting to produce films designated as EL_F (EL/PLA), EL_CF (EL_C/PLA), and EL_MCF (EL_MC/PLA). The glass transition temperatures (*T*_g_) of the films followed a descending trend from EL_MCF to EL_CF, EL_F, and PLA, with the reduced *T*_g_ of the lignin-polyether (LPE)/PLA films indicating a plasticizing effect that enhanced the flexibility of the PLA matrix. Thermal stability (*T*_max_, maximum weight loss temperatures ) was highest for EL_MCF (395.86 °C), followed by EL_F (365.84 °C) and EL_CF (357.2 °C). EL_MCF showed superior mechanical properties with the highest elongation at break (297.7%) and toughness (39.92 MJ/m^3^) compared to EL_CF (242.07% and 28.19 MJ/m^3^) and neat PLA (72.58% and 18.41 MJ/m^3^). The tensile strength hierarchy was PLA (30.88 MPa) > EL_CF > EL_MCF > EL_F, with EL_F demonstrating poor mechanical properties. Additionally, the lignin/PLA films demonstrated remarkable UV-blocking capabilities. The EL_MCF film achieved outstanding UV protection, blocking 99.52% of UVA and 88.95% of UVB rays without significantly compromising transparency. These enhancements in mechanical performance and UV resistance underscore the potential of chemically modified lignin to expand the applicability of PLA in industries requiring durable and UV-stable materials.

**Figure 33:**
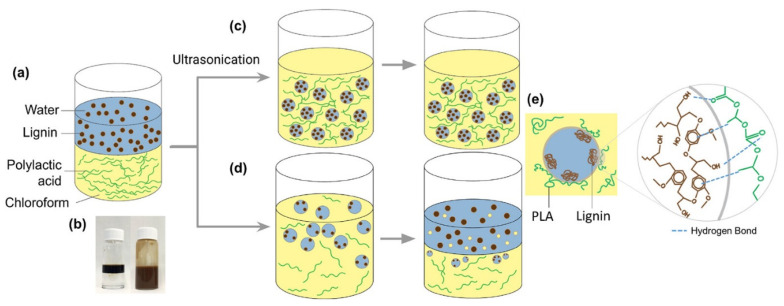
Unstable water-in-oil emulsion to stable dispersion using lignin and PLA. (**a**) Schematic of separate oil and water phases with their constituents before emulsification. (**b**) Mixture appearance before and immediately after sonication. (**c**) Stable W/O emulsion with aqueous lignin droplets suspended in a continuous PLA organic phase. (**d**) Unstable W/O emulsion showing droplet coalescence and phase separation due to insufficient lignin or PLA. (**e**) Close-up of a droplet interface with adsorbed lignin and PLA molecules, highlighting proposed intermolecular interactions [104]. Used with permission, copyright 2023 American Chemical Society.

Yu et al. introduced lignin’s surface activity to stabilize reverse (water-in-oil) emulsions through interfacial interactions with polylactide (PLA) in the organic phase [104]. Unmodified alkali lignin was used to stabilize reverse (water-in-oil) emulsions through interfacial interactions with PLA in an organic, continuous phase (chloroform). Lignin was dispersed in the 1 M NaOH aqueous phase (up to 30 wt.%), while PLA was dissolved in chloroform (up to 10 wt.%). The aqueous lignin and PLA solutions were mixed in various ratios to form emulsions using ultrasonication (Figure 33). The emulsions were cast onto glass plates or coated onto hydrophilic substrates (glass or paper) to create composite films. The lignin–PLA interactions at the oil–water interface were critical for achieving long-term emulsion stability. Uniform composite films with lignin loadings as high as 95% (with PLA being the balance) were obtained by emulsion casting and coating onto hydrophilic substrates. The lignin–PLA films demonstrated enhanced UV-blocking properties, especially in the UV-A region, compared to a commercial sunscreen, while maintaining transparency. The presence of PLA in the continuous matrix of the film imparted water resistance to the coated hydrophilic substrates.

**Figure 34:**
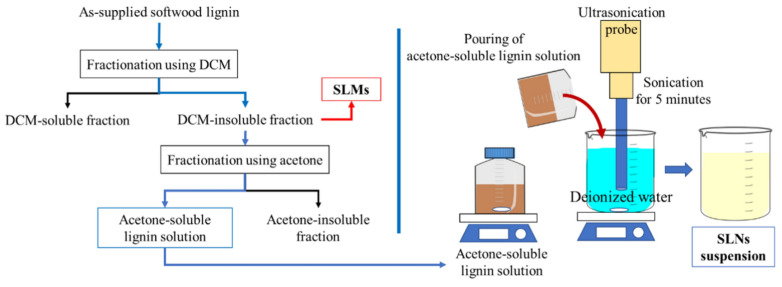
Preparation procedure for lignin nanoparticles (SLNs) and microparticles (SLMs) [104]. Used with permission, copyright 2023 Springer Nature.

Banpean et al. investigated the impact of lignin nanoparticles (SLNs) and microparticles (SLMs) on the crystallization behavior of poly(L-lactic acid) (PLLA) [105]. Softwood kraft lignin was fractionated with dichloromethane (DCM) to produce DCM-insoluble lignin for SLMs, while SLNs were obtained by further fractionating SLMs with acetone, followed by ultrasonication and centrifugation (Figure 34). Composite films with 1 wt.% of either SLNs or SLMs were prepared via solution casting. Both additives enhanced PLLA’s crystallization rate, crystallinity, and nucleation density, with SLNs performing better due to their smaller size. The PLLA/SLN composite required less energy for nuclei formation and had lower folding surface energy than neat PLLA and PLLA/SLM composites. The glass transition temperature (*T*_g_) of PLLA remained unaffected by the addition of SLMs and SLNs.

**Figure 35:**
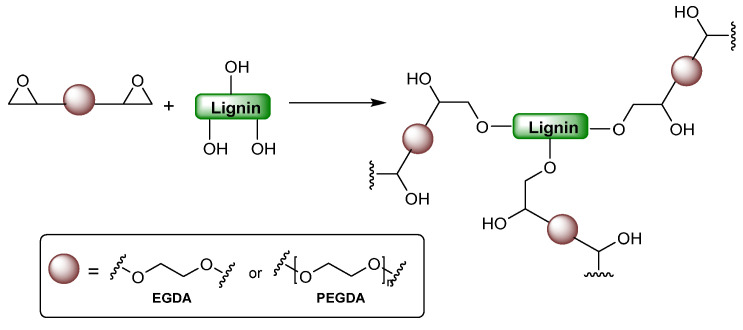
Chemical illustration of the reactivity of ethylene glycol diglycidyl ether (EGDE) and poly(ethylene glycol) diglycidyl ether (PEGDE) with lignin.

Shakoor Shar et al. used low-viscosity epoxy resins, ethylene glycol diglycidyl ether (EGDE), and poly(ethylene glycol) diglycidyl ether (PEGDE) as compatibilizers to enhance lignin dispersion in the PLA matrix (Figure 35) [106]. These epoxies form chemical linkages or hydrogen bonds with PLA and lignin, improving compatibility and interfacial adhesion. PLA/lignin bio-composites were prepared with 1, 3, and 5 phr lignin loadings. Lignin addition increased PLA’s onset degradation temperature by up to 15 °C and improved crystallinity. EGDE and PEGDE reduced the glass transition, crystallization, and melting temperatures, indicating their compatibilizing and plasticizing effects. Oxygen barrier properties were significantly enhanced, with PLA/EGDE/5LG and PLA/PEGDE/5LG showing up to 58.3% and 57.3% enhancement, respectively. Tensile strength observed only a modest increase (~15%), and compatibilizers did not significantly boost tensile strength. The composites demonstrated enhanced thermal stability, crystallinity, and oxygen barrier properties, showing promise for green packaging, though biodegradability was not assessed. This enhancement is attributed to a reduction in micro- and nanoscale porosity and the formation of a more tortuous diffusion path due to homogeneous dispersion of lignin domains within the polymer matrix. These effects collectively suppress oxygen permeability by limiting molecular transport, consistent with barrier improvements reported in lignin–polyester and lignin–PLA systems [115].

**Figure 36:**
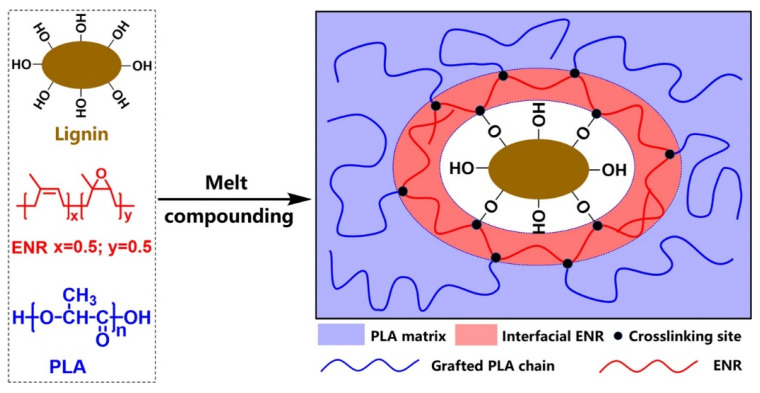
Mechanisms of interfacial reactions in preparing epoxidized natural rubber (ENR) compatibilized PLA/lignin composites [107]. Used with permission, copyright 2023 Elsevier.

Poor mechanical properties limit the applications of PLA/lignin composites due to poor compatibility and low interfacial adhesion. Ou and coworkers addressed this by using epoxidized natural rubber (ENR) as a reactive compatibilizer, enabling the formation of interfacial crosslinking copolymers through reactions between ENR, PLA, and lignin during melt processing (Figure 36) [107]. The addition of ENR reduced interfacial gaps, blurred phase boundaries, and enhanced the elastic response, demonstrating improved compatibility. PLA/lignin/ENR composites were produced by melt compounding at 170 °C with a fixed PLA-to-lignin ratio (80:20) by changing the ENR content (1–10 wt.%). Increasing ENR content raised the glass transition temperature (*T*_g_) of the composites due to reduced free volume and chain mobility. Mechanical testing showed that tensile strength and elongation at break for PLA/lignin (80/20) without ENR were 45.31 MPa and 2.9%, respectively. Tensile strength with 1 wt.% ENR increased by 15% to 52.03 MPa, and elongation at break improved by 77% to 4.6%. While higher ENR content (10 wt.%) further enhanced elongation at break to 20.1%, it reduced tensile strength to 38.37 MPa. Overall, ENR effectively enhanced the mechanical performance of PLA/lignin composites.

**Figure 37:**
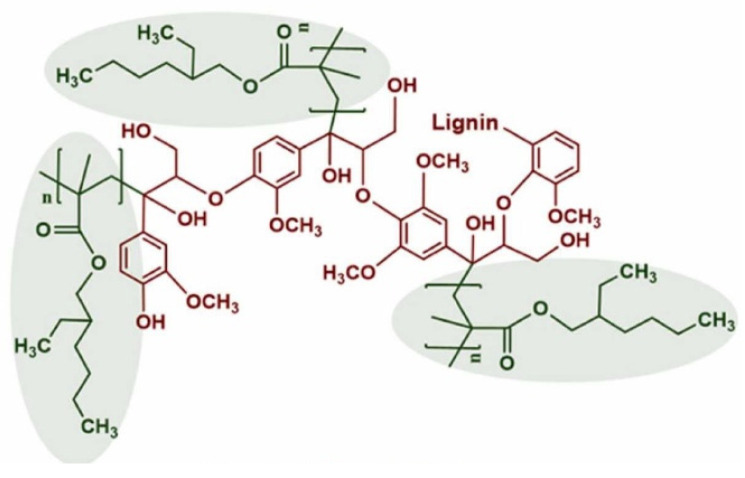
The predicted structure of e-Lignin by grafting 2-ethylhexyl acrylate (EHA) onto lignin [108]. Used with permission, copyright 2023 Elsevier.

**Figure 38:**
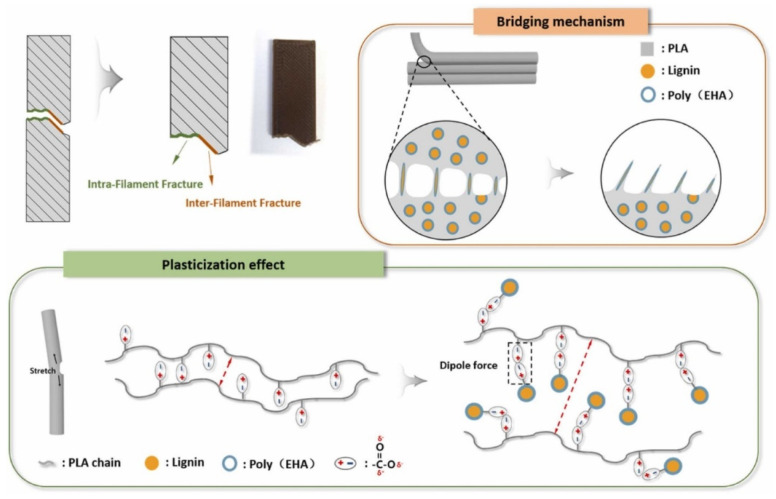
Proposed mechanism for the toughening effect in 3D-printed PLA/e-Lignin composites [108]. Used with permission, copyright 2023 Elsevier.

Ding and coworkers developed PLA/lignin biocomposites for 3D printing by chemically modifying the lignin through grafting 2-ethylhexyl acrylate (EHA) to produce modified lignin (e-Lignin, Figure 37) [108]. The grafting improved interfacial interactions between lignin and PLA, enhancing dispersion and mechanical properties. The low melt viscosity of e-Lignin facilitated thermo-rheological relaxation, promoting PLA molecular chain mobility and making composites suitable for fused deposition modeling (FDM) 3D printing. Different PLA/e-Lignin specimens were prepared, adding 0–15 wt.% of e-Lignin to assess the properties. The addition of e-Lignin showed lower glass transition temperatures (*T*_g_) and improved thermal stability, with higher decomposition temperatures (*T*_5%_) and maximum weight loss temperatures (*T*_max_). Although the storage modulus (*E*′) was lower than pure PLA below 50 °C, indicating reduced stiffness, higher tan *δ* peaks suggested enhanced molecular chain mobility. While tensile strength and modulus decreased, the elongation at break and toughness significantly improved. The 10 wt.% e-Lignin composites showed the highest toughness (3.84 MJ/m^3^) and impact energy (6.36 KJ/m^2^) compared to pure PLA (1.16 MJ/m^3^, 2.12 KJ/m^2^, respectively) in 3D-printed specimens. The enhanced toughness and impact energy were due to the synergistic effects of plasticization and the bridging effect of e-Lignin (Figure 38). The composites maintained suitable melt viscosity for 3D printing, with improved interlayer adhesion due to better interfacial diffusion. However, the long-term performance and aging behavior of the 3D-printed composites need to be assessed.

Makri et al. developed PLA composites incorporating lignin using two distinct techniques: conventional melt-mixing and in situ ring-opening polymerization (ROP) with reactive processing [109]. Composites containing 0.5 wt.% lignin or nano-lignin were investigated, with the in situ ROP method demonstrating a reduction in reaction time as catalyst content increased. Thermal analysis revealed that even at low lignin concentrations, the reactive processing method significantly influenced the thermal transitions of PLA. In the comparison of mechanical properties, the addition of 0.5 wt.% lignin or nano-lignin to neat PLA reduced the stress at break and elastic modulus, while elongation at break remained unaffected. Compared to conventional melt-mixing, where poor lignin dispersion and aggregation resulted in weakened properties, the in situ ROP method achieved superior mechanical performance due to enhanced lignin dispersion and improved interfacial bonding, maintaining or enhancing the material’s mechanical properties.

**Figure 39:**
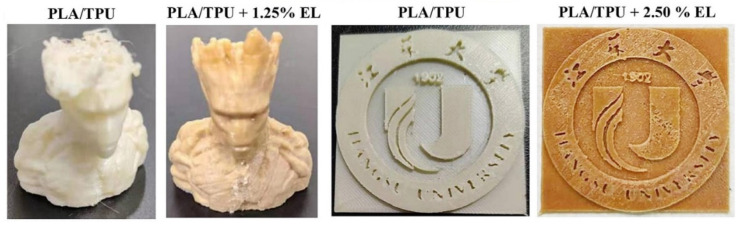
Representative images of routine FDM 3D-printed objects using a PLA/TPU blend alone and a PLA/TPU blend incorporating bacterial enzymatically modified lignin (EL) [110]. Used with permission, copyright 2023 Elsevier.

Murillo-Morales et al. investigated the use of enzymatically modified lignin (EL) as a nucleating agent to enhance the mechanical properties and biodegradability of polylactic acid/thermoplastic polyurethane (PLA/TPU) blends for 3D printing applications (Figure 39) [110]. Alkali lignin (AL), extracted from *Bacillus ligniniphilus*, was partially degraded using a laccase enzyme to produce EL. PLA/TPU blends with varying ratios (90:10, 85:15, and 80:20) were prepared, incorporating AL and EL in different concentrations (1.25–5.00 wt.% based on PLA weight). The lignin-based thermoplastic composites were extruded into filaments and processed using fused deposition modeling (FDM) for 3D printing. Notably, EL addition increased the elasticity modulus and elongation at break compared to AL filaments. The highest elasticity modulus (2.177 GPa) was observed with a low EL content (1.25 wt.%), representing a 239% increase compared to the control (0.9121 GPa). Additionally, the presence of EL led to a more moderate decrease in the molar mass than AL filaments, maintaining viscosity while enhancing elasticity. Overall, the incorporation of EL in PLA/TPU blends significantly improved the mechanical properties of the 3D-printed composites, providing tunable performance, a wood-like color, and enhanced biodegradability.

**Figure 40:**
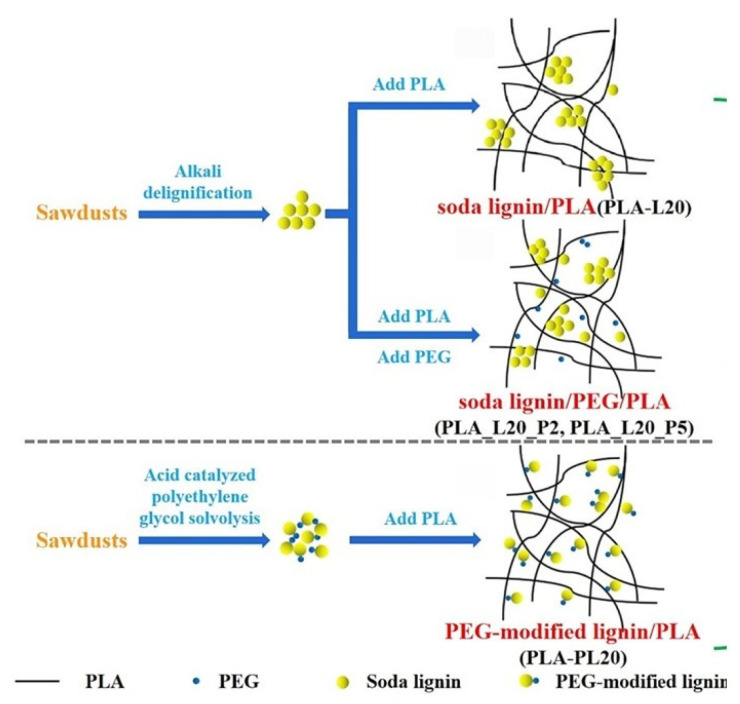
Different methods of polyethylene glycol (PEG) incorporation for the preparation of lignin/PLA composites [111]. Used with permission, copyright 2024 Elsevier.

**Figure 41:**
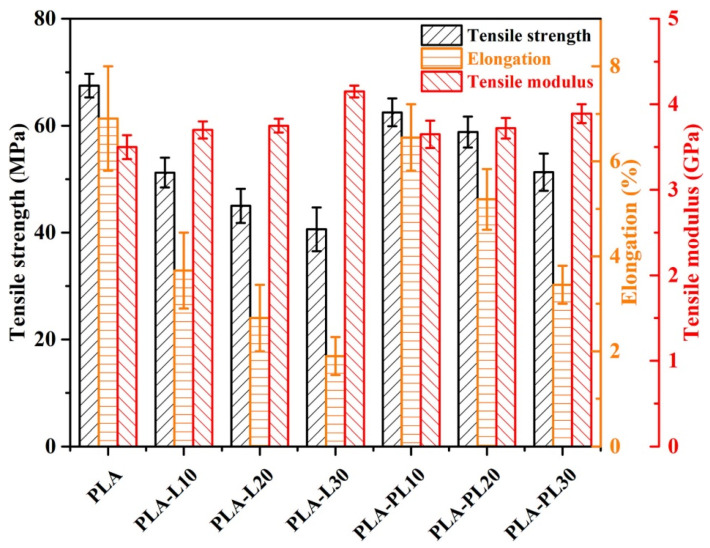
Mechanical properties of neat PLA, PLA composites with unmodified soda lignin (PLA-L), and PLA composites with PEG-modified lignin (PLA-PL) [111]. Used with permission, copyright 2024 Elsevier.

Ju et al. proposed a strategy to enhance the mechanical and thermal properties of polylactic acid (PLA) biocomposites by incorporating polyethylene glycol (PEG)-modified lignin as a functional filler [111]. Soda lignin and PEG-modified lignin were prepared from beech wood via steam blasting pretreatment, followed by two processes: alkali delignification and acid-catalyzed PEG solvolysis (Figure 40). The incorporation of PEG-modified lignin improved the heat resistance of the PLA matrix, enhancing its applicability in thermally demanding environments. Compared to pure PLA, the incorporation of unmodified soda lignin (PLA-L composites) showed a progressive reduction in tensile strength and elongation at break with increasing lignin content (Figure 41). This decline in mechanical properties is attributed to lignin’s tendency to disrupt the continuity of the PLA matrix, inhibiting the formation of a uniform, long-range polymer phase. However, the tensile modulus increased, with PLA-L30 exhibiting an 18.6% higher modulus than neat PLA. In contrast, composites containing PEG-modified lignin (PLA-PL) demonstrated significantly improved mechanical properties. PLA-PL10 composites exhibited a 22% higher tensile strength than PLA-L10, demonstrating the compatibilizing effect of PEG chains. Furthermore, PLA-PL30 showed a 26.4% increase in tensile strength and a 78.9% increase in elongation at break compared to pure PLA, indicating that up to 30 wt.% of PEG-modified lignin can enhance both strength and flexibility, improving the mechanical performance.

**Figure 42:**
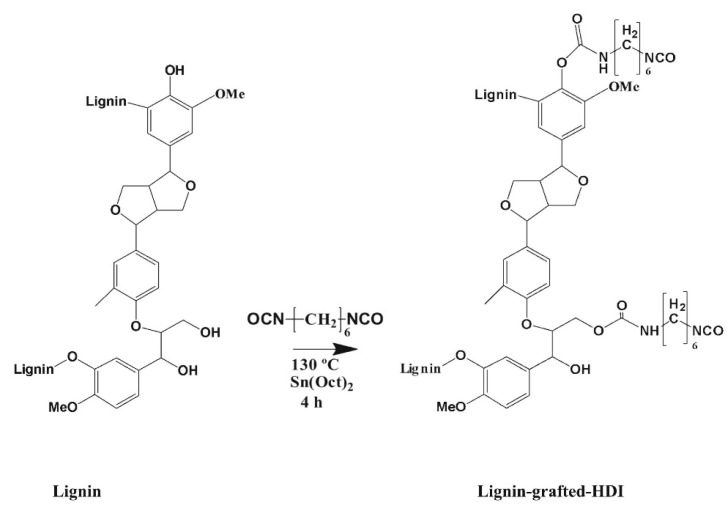
Schematic representation of the preparation method for lignin-grafted polyurethane (L-*g*-HDI) via the reaction of lignin molecules with hexamethylene diisocyanate (HDI) [112]. Used with permission, copyright 2024 Elsevier.

Beniwal and co-workers investigated the improvements in mechanical strength, thermal resistance, and oxygen barrier performance of polylactic acid (PLA) composites through the incorporation of lignin functionalized with hexamethylene diisocyanate (HDI) to form lignin-grafted polyurethane (L-*g*-HDI) structures (Figure 42) [112]. The lignin was chemically modified via a heterogeneous reaction in dimethyl sulfoxide, introducing urethane linkages through HDI-improved compatibility with the PLA by enhancing hydrogen bonding and intermolecular interactions between the lignin and polymer matrix. When blended with PLA, the crosslinked lignin significantly enhanced the mechanical properties of the resulting composites, particularly in terms of tensile strength and elongation at break, due to the interactions. Optimal mechanical performance was observed at 14 wt.% L-*g*-HDI loading, with a remarkable 231% increase in tensile strength, elongation at break reaching 37.8%, and a sixfold increase in Young’s modulus compared to neat PLA. However, further increases in filler content (16 wt.%) led to diminished mechanical performance due to filler agglomeration and disruption of the polymer phase continuity. In addition to mechanical enhancements, the incorporation of L-*g*-HDI resulted in increased crystallinity, improved thermal stability, and enhanced barrier and water-resistance properties.

**Figure 43:**
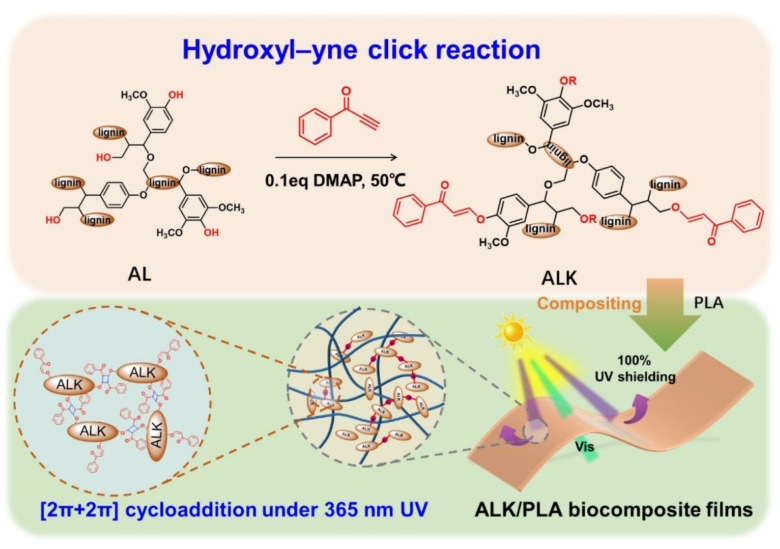
Schematic representation of the preparation of lignin derivatives via the hydroxyl-yne click reaction, enabling the development of UV-shielding and anti-aging alkali lignin/poly(lactic acid) (ALK/PLA) biocomposite films [113]. Used with permission, copyright 2024 Elsevier.

Li et al. developed a sustainable and efficient approach to modify lignin for enhanced performance in poly(lactic acid) (PLA) biocomposites. A mild hydroxyl-yne click reaction was employed to simultaneously functionalize both phenolic and aliphatic hydroxyl groups in alkali lignin (ALK) under low-temperature conditions without generating byproducts (Figure 43) [113]. In here, the hydroxyl-yne click reaction covalently attaches vinyl ether groups to both phenolic and aliphatic hydroxyls in lignin, increasing crosslink density and enhancing compatibility with the PLA matrix. This functionalization restricts polymer chain mobility, thereby improving mechanical strength and modulus. Additionally, the introduced vinyl ether groups contribute to strong UV absorption due to their conjugated unsaturated bonds, indicating excellent UV shielding and stability. Incorporation of 3 wt.% of the modified ALK into PLA resulted in notable improvements in the mechanical properties of the resulting biocomposite films. Specifically, the yield strength, breaking strength, and Young’s modulus increased by 114.2%, 30.7%, and 250.2%, respectively. Furthermore, the introduction of vinyl ether linkages through the click reaction reported excellent UV shielding (100% absorption) and anti-UV aging properties. Even at a low loading of 1 wt.% ALK, the biocomposites exhibited high visible light transmittance (84.3%) alongside remarkable UV stability. Moreover, after 3 h of UV irradiation, the composite maintained superior mechanical performance, with yield strength and Young’s modulus increasing by 209% and 86%, respectively.

**Figure 44:**
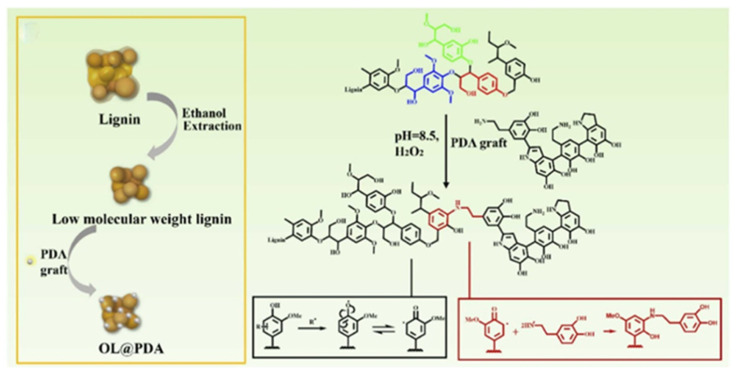
Schematic illustration of the preparation of dopamine-modified lignin (OL@PDA) via the grafting of polydopamine onto low-molecular-weight lignin (OL) [114]. Used with permission, copyright 2024 Elsevier.

**Figure 45:**
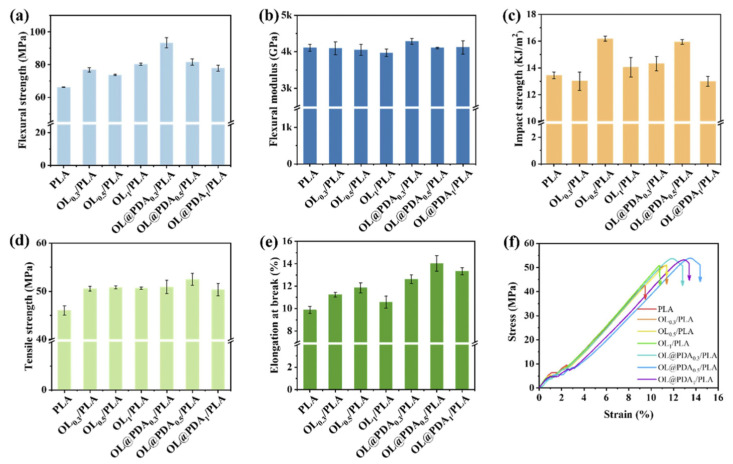
Comparison of mechanical properties between pure PLA and OL@PDA/PLA composites: (**a**) flexural strength, (**b**) flexural modulus, (**c**) impact strength, (**d**) tensile strength, (**e**) elongation at break, and (**f**) tensile stress–strain curves [114]. Used with permission, copyright 2024 Elsevier.

Cao et al. developed a novel bio-based modifier, OL@PDA, to address key limitations of polylactic acid (PLA), such as brittleness, low thermal stability, and vulnerability to hydrolysis. OL@PDA is synthesized by grafting low-molecular-weight lignin (OL) with polydopamine (PDA), combining the structural benefits of lignin’s aromatic rings with the adhesive and compatibility-enhancing properties of PDA (Figure 44) [114]. Incorporating OL@PDA into PLA led to significant performance enhancements. Specifically, the OL@PDA0.5/PLA composite (containing 0.5 wt.% of PDA) exhibited a 14% increase in tensile strength and a 42% increase in elongation at break, along with notable improvements in flexural and impact strength (Figure 45). These enhancements were attributed to improved interfacial compatibility and uniform nanofiller dispersion, which facilitated stress dissipation and triggered a transition from brittle to ductile fracture behavior. Thermal analysis revealed elevated decomposition temperatures, indicating enhanced thermal stability due to stronger interactions between OL@PDA and the PLA matrix. Additionally, OL@PDA significantly reduced UV transmittance from 65.8% (pure PLA) to less than 5%, providing effective UV shielding. While the presence of hydrophilic groups in PDA slightly lowered the contact angle, the overall hydrophobicity remained suitable for industrial use. These findings highlight OL@PDA as an effective and environmentally friendly approach to enhancing the multifunctionality of PLA-based materials.

## 5. Other Strategies for Lignin Incorporation into Materials

### 5.1. Polymers with Covalently Attached Lignin

**Scheme 5:**
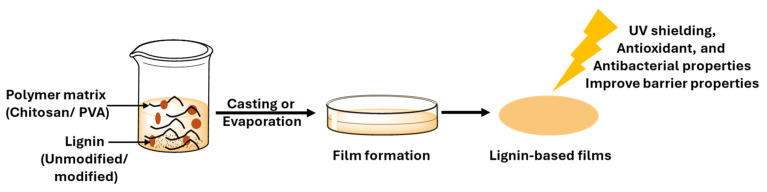
Formation of lignin-based films and coatings via blending or chemical crosslinking.

Non-biodegradable materials significantly contribute to long-term environmental pollution and waste accumulation. Currently, packaging materials account for nearly half of the total municipal solid waste. Therefore, identifying biodegradable alternatives for plastic films is crucial. One promising approach is the incorporation of unmodified or chemically crosslinked (e.g., via aldehyde or epoxy linkers) lignin into biodegradable plastics through blending or chemical crosslinking. Films can be formed via casting or solvent evaporation. In these systems, lignin not only enhances the mechanical strength and gas barrier properties of food packaging films but also imparts valuable functionalities such as antioxidant, antibacterial, and UV-blocking capabilities (Scheme 5) [116,117,118,119].

**Figure 46:**
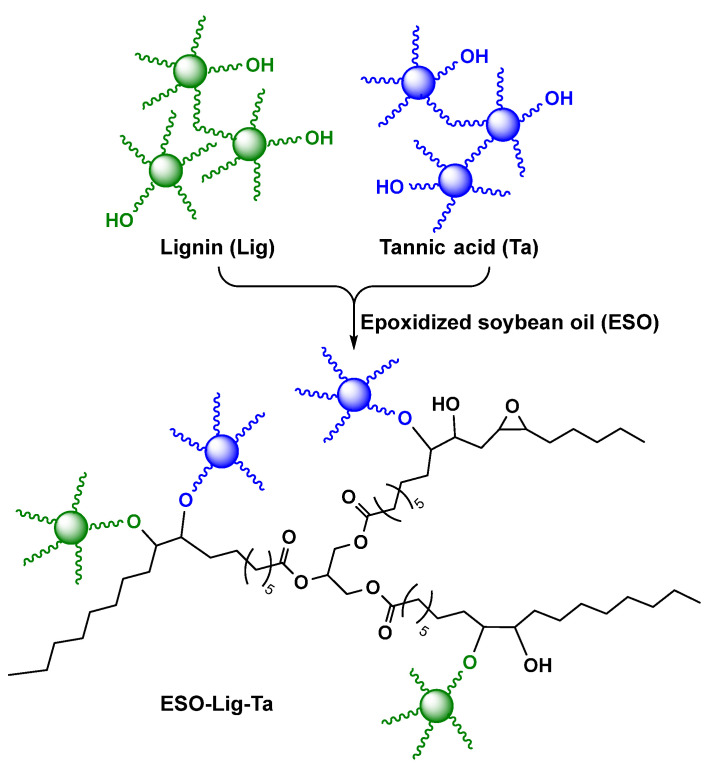
Schematic representation of the preparation of ESO-Lig-Ta by modifying lignin with tannic acid and epoxidized soybean oil.

Olonisakin and coworkers prepared a biodegradable polymer using lignin and tannic acid as fillers to modify poly(butylene adipate-*co*-terephthalate) (PBAT) [120]. Initially, lignin and tannic acid (Ta) were microencapsulated and mixed with epoxidized soybean oil (ESO) to form ESO-Lig-Ta (Figure 46), and the different amounts of ESO-Lig-Ta (0–3 wt.%) were then incorporated into the PBAT matrix to form PBAT-ESO-Lig-Ta composite film. Microencapsulation prevents premature aggregation of lignin and tannic acid, ensuring their uniform distribution within aqueous or polymeric matrices. It also enables controlled release functionality, improves interfacial adhesion, and protects phenolic hydroxyl groups from oxidative degradation—benefits that are particularly relevant in biomedical and barrier applications where component stability and homogeneity are critical. The modification involved the reaction between the phenolic groups of lignin and tannic acid with the epoxy rings of ESO, forming ether linkages in the ESO-Lig-Ta network, and a stable composite was formed by reacting ESO with the end groups of PBAT chains. ESO-Lig-Ta fillers improved the tensile strength, thermal stability, barrier properties, and biodegradability of the PBAT films. The loading of 3 wt.% ESO-Lig-Ta enhanced the UV-blocking ability by completely blocking UV-B radiation and reduced water vapor and oxygen transmission rates compared to neat PBAT, making them suitable for food packaging applications. The presence of lignin and tannic acid accelerated the biodegradation process through the hydrolysis of ester bonds to improve the sustainability of the film. Tensile strength values increased for 1 wt.% (7.27 MPa) and 2 wt.% ESO-Lig-Ta (6.55 MPa) compared to neat PBAT (3.13 MPa), and elongation at break decreased with increasing ESO-Lig-Ta loading compared to neat PBAT. For real-world applications of this film, further research may need to assess its long-term durability under varying packaging environments, such as changes in humidity, temperature, and mechanical stresses during handling and transportation.

**Figure 47:**
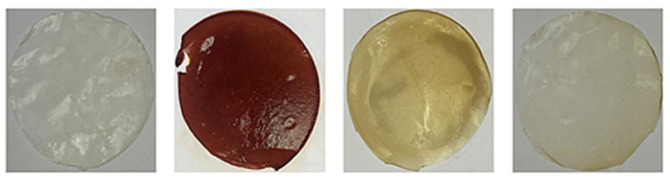
Visual appearance of keratin film, alkali lignin film, decolorized/ modified lignin by laccase, and decolorized/modified lignin by dye-decolorizing peroxidase (left to right) [121]. Under a Creative Commons Attribution (CC BY) license (https://creativecommons.org/licenses/by/4.0/ accessed on 27 April 2025).

Waqas Ali Shah and co-workers examined the preparation and properties of biodegradable films produced by grafting alkali lignin (AL) and enzymatically modified alkali lignin (EMAL) onto keratin using laccase and dye-decolorizing peroxidase enzymes [121]. Keratine and lignin components were dissolved separately, mixed with glycerol, cast into films, and dried before further analysis. The visual appearance of films is represented in Figure 47. The incorporation of EMAL improved the tensile strength, elongation at break, and thermal stability, and acted as an antimicrobial and antioxidant in the films. The films with EMAL modification showed greater tensile strength (14.8 ± 1.8 MPa) and elongation at break (23.7 ± 0.3%). The films exhibited good antibacterial properties against *Staphylococcus aureus* and *E. coli* and high antioxidant activity. Overall, the study introduced an approach for lignin valorization to produce sustainable biomaterial films with improved properties.

**Figure 48:**
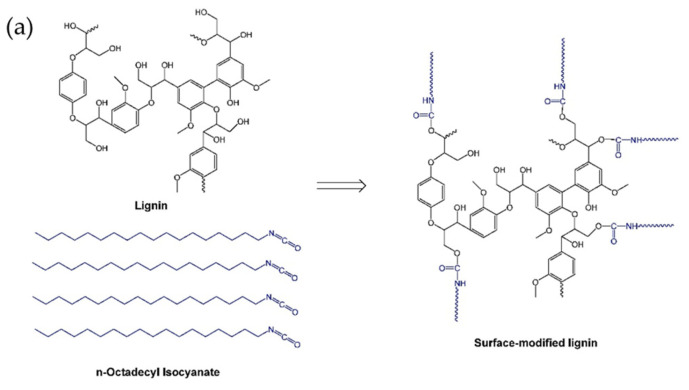

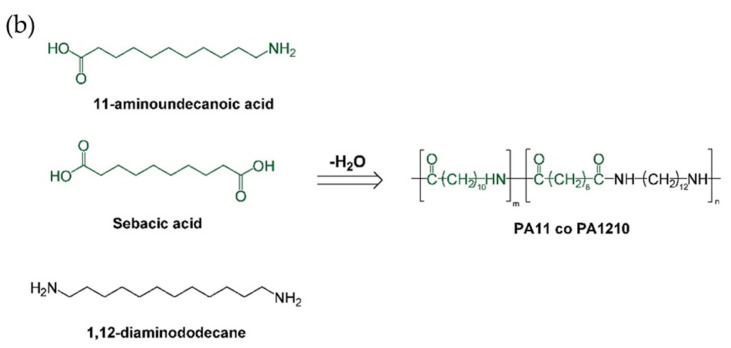
(**a**) Schematic representation of the surface-modified lignin preparation and (**b**) copolymerization of castor oil-based and petroleum-based monomers to form the copolyimide matrix [123]. Under a Creative Commons Attribution (CC BY) license (https://creativecommons.org/licenses/by/4.0/ accessed on 27 April 2025).

The production and refining of petroleum-derived polymers contribute to environmental degradation, including high carbon emissions and the depletion of non-renewable resources. Bio-based fillers, derived from renewable resources, are essential to reduce the reliance on petroleum-based raw materials [122]. Baniasadi and coworkers successfully developed sustainable, high-performance bio-composites using alkali lignin particles [123]. These lignin particles were surface-modified by grafting *n*-octadecyl isocyanate molecules onto the lignin hydroxyl groups via a urethane reaction (Figure 48a). The copolyamide matrix (PA11coPA1210) was synthesized by copolymerizing castor oil-based monomers (11-aminoundecanoic acid and sebacic acid) with a petroleum-based monomer (1,12-diaminododecane) (Figure 48b). Following the surface modification of lignin particles, these were incorporated (10–50 wt.% of modified lignin) into the copolyimide matrix to develop a low-melting-point bio-based film. The results showed a significant increase in storage modulus with higher lignin content, indicating improved component compatibility, mechanical properties, and bio-content through tailored lignin surface chemistry and copolymer composition. Future work would be needed to optimize lignin modification processes by exploring alternative bio-based monomers and non-toxic compounds to enhance the performance and sustainability of these composites.

**Figure 49:**
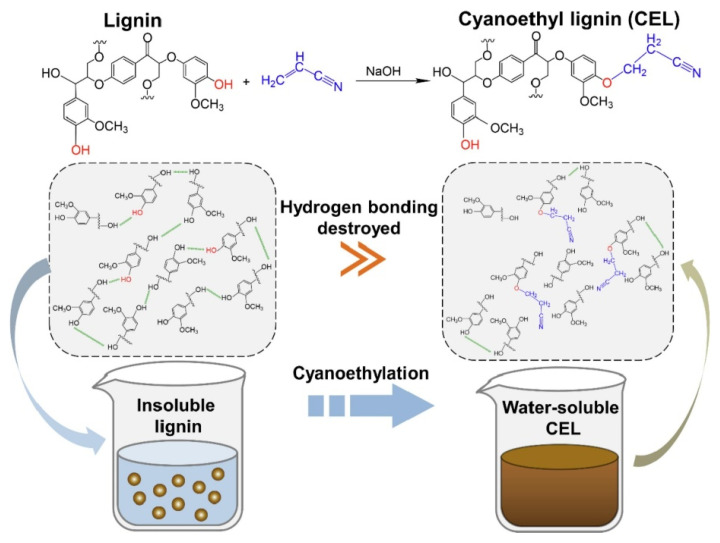
Reactions in the formation of cyanoethyl lignin (CEL) and possible mechanisms of water solubility of CEL [124]. Used with permission, copyright 2023 Elsevier.

Tian and their research group modified lignin through a cyanoethylation reaction to make it water-soluble. Cyanoethylation is an etherification reaction where phenolic hydroxyl groups on lignin are substituted with cyanoethyl groups, improving water solubility due to disruption of hydrogen bonding between lignin fragments (Figure 49) [124]. This was completed under mild conditions using acrylonitrile. The cyanoethylated lignin (CEL) was then used to modify poly (vinyl alcohol) (PVA) as the polymer matrix. CEL acted as both a biomimetic bonding adhesive and a functional additive. It was coated onto cellulose nano-whiskers (CNWs) to form a multi-functional building block with a mussel-inspired layered structure. This CEL@CNW building block was blended with an aqueous PVA solution to create a liquid mulching film suspension. CEL improved the thermal stability of the PVA composite films by decreasing the maximum thermal decomposition rate compared to pure PVA or PVA/CNW films, and the tensile strength of the PVA/CEL@CNW films decreased as the percentage of CEL@CNW increased. Specifically, the tensile strength decreased from 33.6 MPa for pure PVA to 25.2 MPa for PVA/10% CEL@CNW. Overall, CEL modification improved the compatibility of lignin (typically insoluble) with the PVA matrix, including various performance properties like thermal stability, mechanical strength, and barrier abilities due to aromatics and phenolic functionalities in lignin.

**Figure 50:**
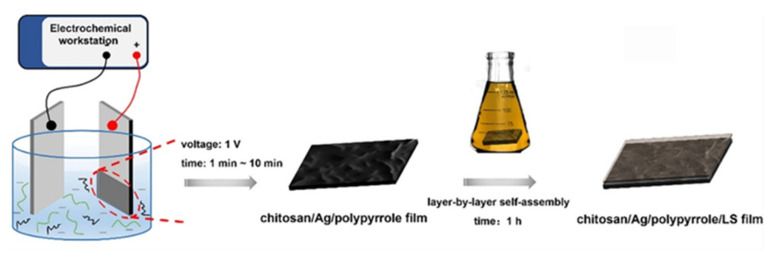
The synthetic pathway of silver nanoparticle (AgNP) doped chitosan/sodium lignin sulfonate/polypyrrole (p_1_i_1_) films [125]. Used with permission, copyright 2023 Elsevier.

Xu and coworkers designed a silver nanoparticle (AgNP) doped chitosan/sodium lignin sulfonate/polypyrrole (p_1_i_1_) film with antibacterial and endotoxin adsorption properties. The films were fabricated using a one-step electrodeposition method to produce a chitosan/Ag/polypyrrole film, followed by electrostatic layer-by-layer self-assembly of lignin sulfonate (Figure 50) [125]. Lignin sulfonate (LS), a negatively charged polyelectrolyte, was introduced through electrostatic interactions with the positively charged chitosan. The self-assembly of LS increased the film’s negative zeta potential and hydrophilicity. The adsorption experiments found that the electrostatic interactions between chitosan and endotoxins played a significant role in removal, with efficiencies up to 85–87% within 1 h. Overall, the film with LS showed the inhibition of *E. coli* and *S. aureus* bacteria and rapid adsorption of endotoxins released from decomposed bacteria, indicating that the addition of LS improved antibacterial effects.

**Figure 51:**
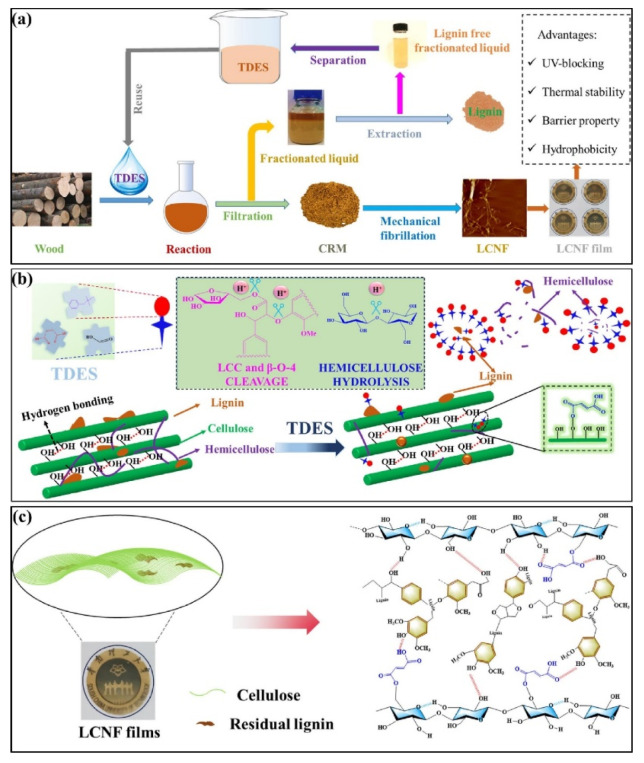
(**a**) Schematic illustration of TDES pretreatment process, (**b**) pretreatment mechanisms of TDES on radiata pine, and (**c**) the structural linkages between lignin and cellulose in the LCNF [126]. Used with permission, copyright 2023 Elsevier.

This study investigated the use of a ternary deep eutectic solvent (TDES) based on benzyl trimethylammonium chloride, formic acid, and maleic acid for the pretreatment of radiata pine biomass (Figure 51) [126]. The TDES removed 87.80% of lignin and 89.15% of hemicellulose while preserving most cellulose. The pretreated material was then mechanically fibrillated to produce lignin-containing cellulose nanofibrils (LCNFs). The LCNF films showed good mechanical properties, thermal stability, and barrier properties against oil and air, indicating potential applications as packaging materials. The LCNF-130 (treated at 130 °C) film achieved the highest tensile strength at 52.0 MPa and exhibited superior ductility with an elongation at break of 8.4%, attributed to its lower lignin content. LCNF films with reduced lignin content also demonstrated increased elastic modulus, suggesting improved stiffness and resistance to deformation. Overall, the TDES pretreatment conditions improved the tensile strength, elongation at break, and elastic modulus of the LCNF films.

**Figure 52:**
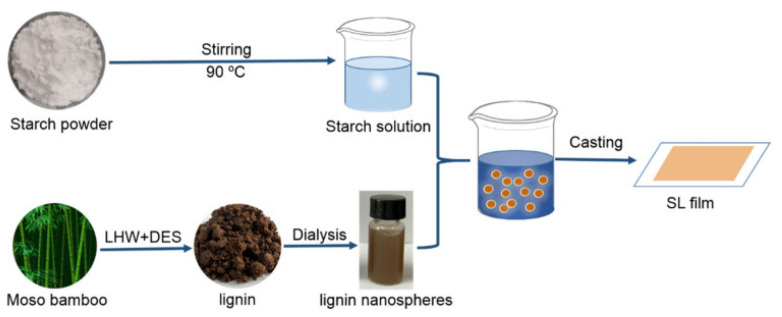
The preparation pathway of the starch/LNPs composite film [127]. Under a Creative Commons Attribution (CC BY) license (https://creativecommons.org/licenses/by/4.0/ accessed on 27 April 2025).

**Figure 53:**
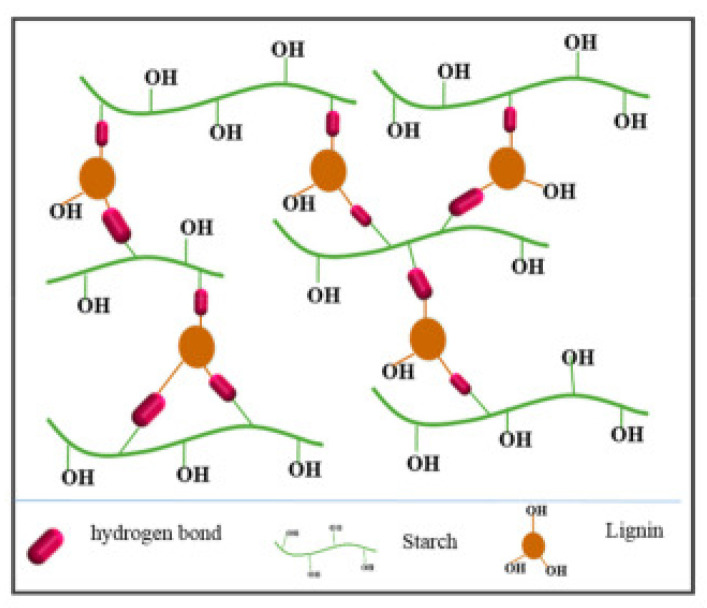
The schematic illustration of the interaction between LNPs and starch matrix [127]. Under a Creative Commons Attribution (CC BY) license (https://creativecommons.org/licenses/by/4.0/ accessed on 27 April 2025).

Sustainable composite biofilms, which incorporate lignin nanoparticles (LNPs) into a starch matrix, were developed by Sun and coworkers to enhance the mechanical, thermal, optical, and antioxidant properties of films used in food packaging. LNPs were produced from Moso bamboo lignin using a two-step pretreatment and self-assembly process, and starch/LNP composite films were made using solution casting by adding different concentrations (0–5 wt.%) of LNPs (Figure 52) [127]. The modification after adding LNPs significantly improved the composite film’s tensile strength (48.9 MPa) and elastic modulus (2288.8 MPa) compared to the pure starch film. Figure 53 illustrates the distribution of LNPs in the starch matrix, forming hydrogen bonds with good compatibility and physical reinforcement with starch to improve properties. Additionally, the composite films exhibited excellent UV shielding. They significantly delayed the oxidative deterioration of soybean oil during storage by lowering its peroxide, saponification, and acid values, indicating their potential application in food packaging.

**Figure 54:**
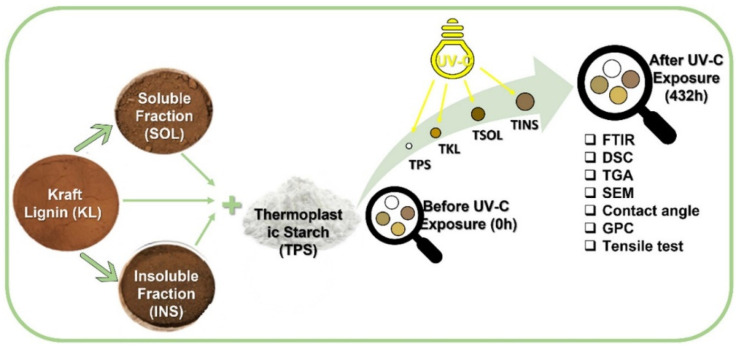
Preparation and characterization of KL-modified TSP films [128]. Used with permission, copyright 2023 Elsevier.

In their study, Rodrigues et al. prepared thermoplastic starch (TPS) films with additions of different fractions of kraft lignin (KL) to enhance their properties after exposure to ultraviolet C (UV-C) light [128]. Using ethyl acetate as a solvent, KL from *Eucalyptus* wood was fractionated into insoluble (INS) and soluble (SOL). Films were prepared by mixing the lignin fractions (4 wt.% KL, SOL, INS) into a TPS matrix. The films were exposed to UV-C (254 nm) light for 432 h and analyzed before and after exposure. Figure 54 represents the preparation and characterization of KL-modified TSP films. SOL-modified TSP films exhibited the highest photoprotection and stabilizing effect as a UV blocker in the TPS matrix due to higher phenolic content. The maximum force of SOL-modified TSP (14.37 N) was improved compared to pure TSP (5.58 N) after exposure to UV, indicating the enhancement of crosslinking between hydroxy functionalities in lignin. Overall, SOL-modified TSP films showed minimal structural changes, better thermal stability, higher crystallinity, and improved mechanical properties after UV exposure compared to other films.

Pham and co-workers created a chitosan film incorporating lignin, obtained as a byproduct from the black liquor generated during the cellulose isolation process from rice straw without further modification. The different concentrations of lignin (0–8 g/L) were combined with a 2% (*w*/*v*) solution of chitosan (a biopolymer derived from shrimp shells) to form chitosan/lignin composite films via a simple solution casting method [129]. The addition of lignin to the chitosan films improved the UV-blocking ability (100% UV light blocking even at low lignin concentrations), antioxidant activity (DPPH radical scavenging activity increased from 56% for pure chitosan to over 91% with lignin), and antimicrobial activity against *E. coli* and *C. albicans*, and reduced water vapor permeability compared to pure chitosan films. This enhancement is attributed to the intrinsic UV-absorbing capacity of lignin’s conjugated aromatic structures, which strongly absorb in the 280–340 nm range. Additionally, lignin’s phenolic hydroxyl groups act as free radical scavengers, disrupting UV-induced oxidative chain reactions and thereby stabilizing the chitosan matrix against photodegradation. The films with 2 g/L lignin concentration showed the highest tensile strength of 16.637 N/mm^2^ compared to 6.578 N/mm^2^ for the pure chitosan film. Conversely, a lower lignin content (1 g/L of lignin) exhibited a higher elongation at break than pure chitosan, which decreased with increasing lignin loading. Furthermore, high lignin incorporation (8 g/L lignin) showed the highest Young’s modulus of 676.578 N/mm^2^ compared to pure chitosan film (13.986 N/mm^2^), indicating that lignin acts as a rigid filler, stiffening and reinforcing the chitosan matrix. Overall, low-to-moderate lignin loadings enhanced tensile strength and elongation, while high lignin concentrations increased stiffness but compromised the tensile properties of the chitosan films due to possible agglomeration effects.

**Figure 55:**
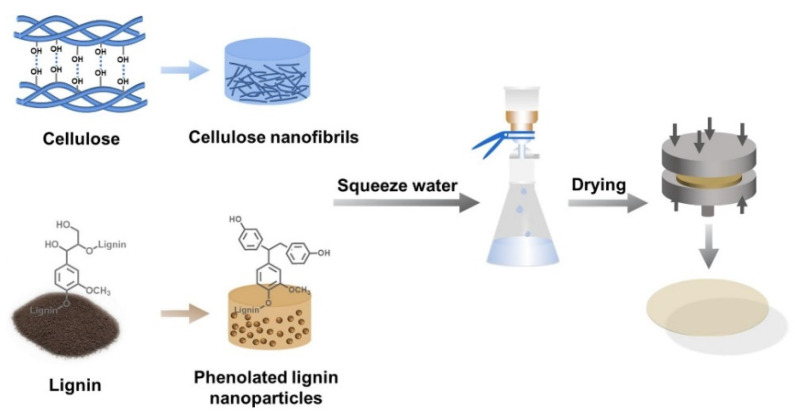
The schematic diagram for preparing LNP/CNF composite film [130]. Used with permission, copyright 2023 Elsevier.

**Figure 56:**
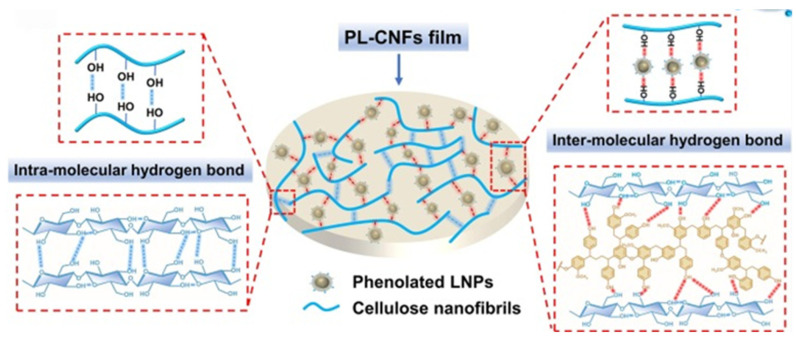
The proposed strengthening effects of phenolated LNP (P-LNPs/CNF) films exhibiting the hydrogen bonds [130]. Used with permission, copyright 2023 Elsevier.

Ou and researchers modified lignin through phenolation and employed the resultant lignin nanoparticles (LNPs) to reinforce both cellulose nanofibril (CNF) composite films as well as polyvinyl alcohol (PVA) films. The kraft lignin (KL) was reacted with phenol under acidic conditions to increase phenolic hydroxyl group content and reduce lignin’s molecular weight. The anti-solvent precipitation method was used to convert phenolated lignin to LNPs, and different amounts of LNPs (0–30 wt.%) were mixed with CNFs to prepare LNP/CNF films (Figure 55) [130]. LNP/PVA films were prepared by mixing 5 wt.% LNPs and 5 wt.% of PVA. The strengthening effect of introducing phenolated LNPs (P-LNPs) into CNF films is shown in Figure 56, indicating intermolecular hydrogen bonds. The formation of more hydrogen-bonding interactions between LNPs and hydroxyl groups in CNFs or PVA exhibited a high dispersity of LNPs, and significantly improved tensile strength (~190 MPa), toughness (~15 MJ/m^3^), and UV-shielding properties (~99%) of LNP-inserted films compared to the neat CNF/PVA films or composites with unmodified lignin.

In another study, Gao et al. developed a lignin-modified film through the amination of lignin. Lignin was aminated via the Mannich reaction to produce aminated lignin (aL) and complexed with Fe(III) by impregnating it in a Fe(NO_3_)_3_ solution to form the aL/Fe(III) complex [131]. Then, the complex was blended with polyvinyl alcohol (PVA) in different ratios (0:10, 1:9, 1:4, 3:7, 2:3, and 1:1) in an aqueous solution, and the mixture was cast into a film named (aL/Fe(III)/PVA). The tensile strength of the aL/Fe(III)/PVA (3:7) film was increased to 3.6 MPa compared to pure PVA (3.3 MPa). Elongation at break and fracture resistance also increased compared to PVA film by 107.8% and 21.9%, respectively. During the reaction, the introduced amino groups on lignin enhance hydrogen bonding and interfacial interactions with the hydroxyl-rich PVA matrix, while Fe(III) chelation forms coordination crosslinks that improve structural integrity. This dual crosslinking network results in enhanced tensile strength and elongation. Overall, the synergistic effects between the lignin, PVA, and the iron chelation enhanced the performance of the final material, including mechanical, UV-blocking, hydrophobicity, and slow-release properties compared to pure PVA.

**Figure 57:**
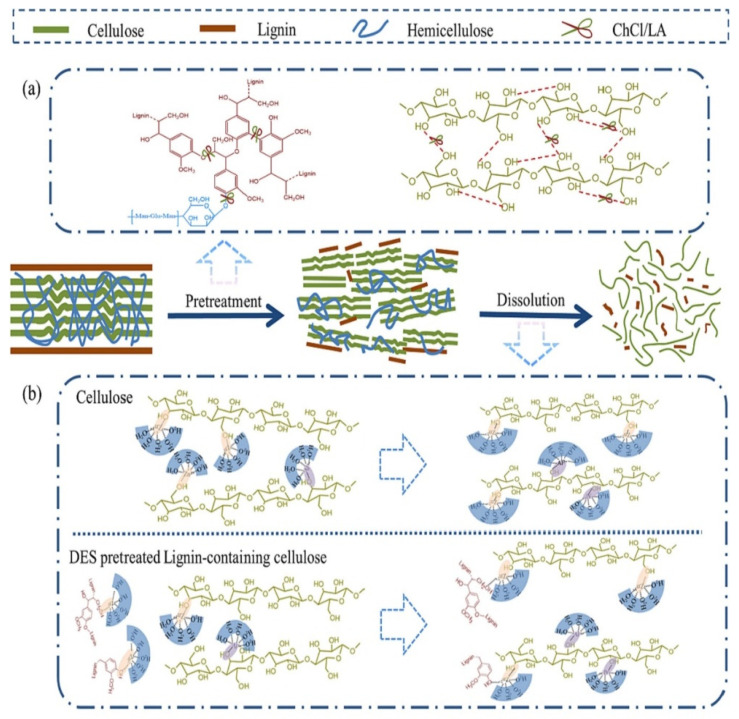
Descriptive diagram for DES pretreatment on lignin and cellulose (**a**), dissolution process of cellulose and (**b**) lignin-containing cellulose after DES pretreatment in AlCl_3_/ZnCl_2_·3H_2_O solutions [132]. Used with permission, copyright 2023 Elsevier.

Jiang H. and coworkers reported sustainable packaging material using biodegradable lignocellulose. As an initial step, deep eutectic solvent (DES) pretreatment (a mixture of choline chloride and lactic acid) was employed at a designated temperature and time to improve the solubility of lignin-containing cellulose (CL), varying lignin contents (14.1–21.2 wt.%) [132]. The modification facilitated the dissolution in the AlCl_3_/ZnCl_2_ aqueous system, reducing the water demand for the dissolution process (Figure 57). The dissolved CL samples were regenerated as lignocellulose films (FCLs) by casting and solvent removal. Due to their lignin content, the FCL films exhibited excellent UV-blocking, hydrophobic, mechanical strength, and natural degradation properties. The primary thermal decomposition for all samples occurred between 250 and 350 °C, and CL films with 14.1–15.6% lignin contents showed higher tensile strengths.

**Figure 58:**
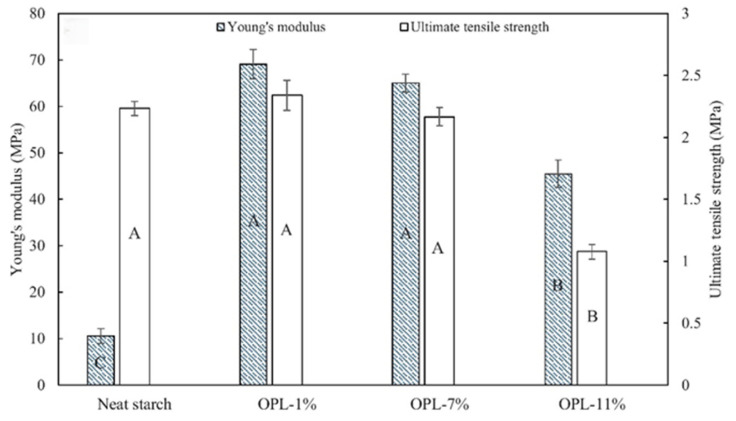
Mechanical characterization data of neat starch and the lignin-modified bio-based films [133]. Different letters (A, B and C) indicate significant differences at *p* < 0.05. Used with permission, copyright 2024 Elsevier.

In another study, Zolfaghari et al. developed an eco-friendly biopolymeric film derived from potato crop residues as a sustainable alternative to conventional plastic food packaging [133]. Lignin was extracted using the organosolv method, achieving a high recovery yield of 73.5 wt.% under optimized conditions. The extracted lignin was incorporated into a starch matrix to form biocomposite films, and their physicochemical properties were compared with those of neat starch films. Notably, the addition of 1 wt.% lignin increased thermal stability by 25% and antioxidant properties by 78%, while reducing oxygen permeability by 87%. Furthermore, films containing 3 wt.% lignin exhibited the lowest water vapor transmission rate, indicating superior barrier performance and increased resistance to moisture penetration. Mechanical characterization (Figure 58) revealed that the incorporation of 1 wt.% lignin significantly improved Young’s modulus and maintained comparable ultimate tensile strength relative to the neat starch films. These enhancements were attributed to strong intermolecular interactions between the lignin and starch hydroxyl groups. However, increasing lignin content beyond 1 wt.% resulted in a decline in tensile properties, likely due to the inherent brittleness of lignin disrupting the polymer network. Overall, films containing 1 wt.% lignin demonstrated the most optimized performance, showing considerable potential for use in sustainable food packaging.

**Figure 59:**
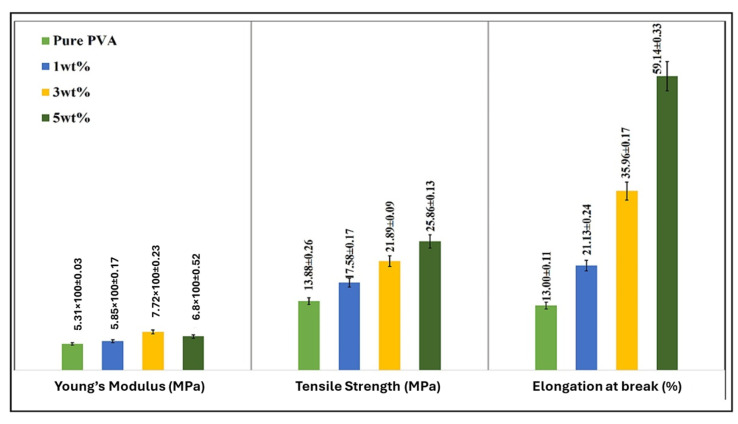
Mechanical characterization data of the prepared lignin-modified films [134]. Used with permission, copyright 2023 Elsevier.

Warale and co-workers fabricated novel poly(vinyl alcohol) (PVA) films by incorporating silver-embedded clay and organosolv lignin via a solution casting approach [134]. A series of composite films was prepared by varying the weight percentage (0–5 wt.%) of lignin-functionalized silver nanoparticles with halloysite nanotubes (Lig/AgNP-HNTs). With increasing Lig/AgNP-HNTs content, the films exhibited enhanced thermal and mechanical stability compared to neat PVA. As illustrated in Figure 59, the film containing 5 wt.% Lig/AgNP-HNTs exhibited the highest tensile strength (28.56 MPa) and elongation at break (59%), while the film with 3 wt.% addition showed a higher Young’s modulus. Furthermore, compatibility tests suggested that these lignin-modified films hold promise as potential wound-healing materials, pending further validation through pre-clinical and clinical evaluations.

**Figure 60:**
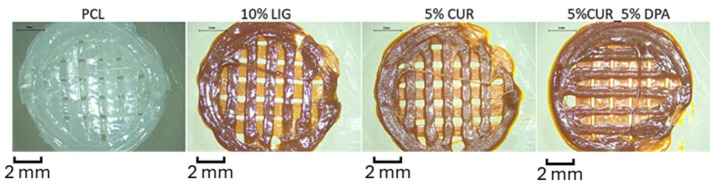
Light microscopic images of the 3D-printed wound dressings containing different proportions (*w*/*w*) of all the compounds (Scale bar is 2 mm) [135]. Under a Creative Commons Attribution (CC BY) license (https://creativecommons.org/licenses/by/4.0/ accessed on 27 April 2025).

Domínguez-Robles and researchers developed wound dressing (medical) materials with antioxidant and antimicrobial properties by incorporating curcumin (CUR) and lignin (LIG) into poly(caprolactone) (PCL), a biocompatible polymer (Figure 60) [135]. *D*-panthenol (DPA) was also included in the formulation of composite materials due to its skin regenerative ability, and materials were manufactured using semi-solid extrusion (SSE) 3D printing without solvents. These 3D-printed dressings sustained DPA and CUR release for 4 and 35 days, respectively. The presence of LIG and CUR provided antioxidant properties to the 3D-printed dressings, as confirmed by the DPPH (2,2-diphenyl-1-picrylhydrozyl) assay. The 3D-printed materials showed a marked resistance to the adherence of *Staphylococcus aureus*, with substantial reductions of up to 89.9% and 98.9% after 4 h and 24 h of incubation, respectively. The combination of CUR and DPA exhibited the most significant values for cell viability and proliferation in in vitro studies. In vivo, wound-healing studies in Wistar rats showed that the dressings containing CUR and DPA exhibited marked improvement at any stage of the treatment process.

**Figure 61:**
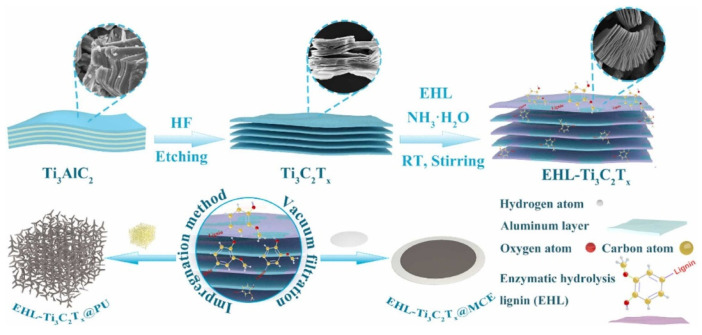
Preparation processes of EHL-Ti_3_C_2_T_x_, EHL-Ti_3_C_2_T_x_ modified PU sponge, and MCE membrane [136]. Used with permission, copyright 2023 Elsevier.

**Figure 62:**
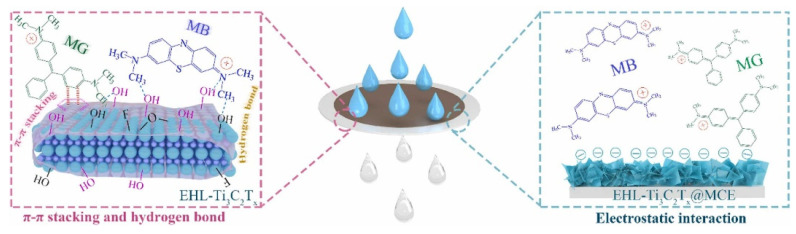
The different mechanisms involved in dye separation using EHL-Ti_3_C_2_T_x_ and EHL-Ti_3_C_2_T_x_@MCE membrane [136]. Used with permission, copyright 2023 Elsevier.

Surface bionics and interfacial chemistry advancements have led to the development of specially designed wettability materials, enabling efficient oil–water separation. Superhydrophobic and superoleophilic materials, when combined with lignin, demonstrate exceptional capabilities in removing oil, dyes, and other organic impurities from water [136,137,138,139,140,141]. Recently, titanium carbide-based separation materials (Ti_3_C_2_T_x_) have been utilized in wastewater treatment. However, their limited functionality restricts their effectiveness in treating complex oily wastewater. Modifying the surface is one of the best solutions to overcome this concern. Wang et al. functionalized the surface of Ti_3_C_2_T_x_ with enzymatic hydrolysis lignin (EHL), which increased its hydrophilicity and stability through secondary bonds formed by its oxygen-containing groups on the surface of the Ti_3_C_2_T_x_ MXene [136]. Figure 61 exhibits the preparation pathway of EHL-functionalized Ti_3_C_2_T_x_ (EHL-Ti_3_C_2_T_x_). Films were applied to the surface of the PU sponge and mixed cellulose ester (MCE) membrane to prepare the EHL-Ti_3_C_2_T_x_@PU sponge and EHL-Ti_3_C_2_T_x_@MCE membrane, respectively. According to the results, the EHL-Ti_3_C_2_T_x_@PU sponge efficiently separated various oil–water mixtures (>99.5%) with a permeation flux of up to 321 Lm^−2^ h^−1^ in the case of a cyclohexane/water mixture and showed excellent chemical stability in corrosive environments. Moreover, the EHL-Ti_3_C_2_T_x_@MCE membrane selectively removed cationic dyes, achieving rejection rates of 99.1% for malachite green and 98.1% for methylene blue, with ~72% permeation flux compared to pure water. Overall, the super-hydrophilic micro-scale pore structure in functionalized PU significantly improved oil–water separation efficiency. The enhanced interactions and considerable interlayer distance in functionalized MCE membranes yielded highly effective dye removal (Figure 62). Further research would be needed to overcome the potential fouling, challenges in scalability, selective removal of specific contaminants, and uncertain long-term stability in real-world wastewater treatment.

In another study, Huang et al. developed a magnetic, superhydrophobic coating using lignin [137]. Hydrothermally treated lignin was synthesized by combining with Fe_3_O_4_ through a combination of solvent displacement and hydrothermal processing. These particles served as the primary building blocks, while nanocellulose crystals (CNCs) and titanium dioxide (TiO_2_) acted as reinforcing agents. Polydimethylsiloxane (PDMS) was used as an adhesive matrix. The coating was applied on a PU sponge using a simple one-step spray or dip-coating method. The resulting material exhibited remarkable durability, enduring up to 87 abrasion cycles due to the synergistic strengthening effect of lignin, CNC, and TiO_2_. In addition to its excellent UV resistance, the coating exhibited remarkable oil absorption capabilities. When applied to a PU sponge, it achieved an absorption capacity ranging from 17.17 to 24.78 g/g for various oils. Moreover, the magnetic properties of the material allowed the sponge to be easily retrieved using a magnet.

**Figure 63:**
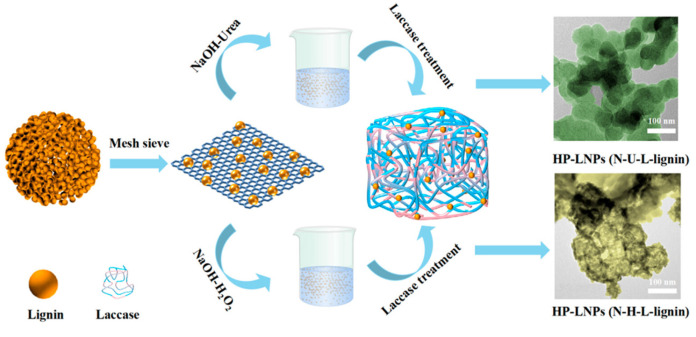
Schematic illustration for the preparation of HP-LNPs [142]. Used with permission, copyright 2023 American Chemical Society.

Yu and coworkers developed highly phenolic lignin nanoparticles (HP-LNPs) that exhibit excellent antioxidant properties and UV resistance. A green and economical strategy was used to modify industrial lignin, and a mild and efficient enzymatic treatment with laccase was employed to prepare the HP-LNPs (Figure 63) [142]. The alkaline treatments with urea and hydrogen peroxide (H_2_O_2_) were used to enhance the antioxidant properties and reduce the visible light absorption of the LNPs. Modification of lignin enhanced its desirable properties, and the HP-LNPs exhibited excellent antioxidant properties, with DPPH and PTIO radical scavenging rates of 76.12% and 51.50%, respectively. The whiteness value of the HP-LNPs increased from 43.50 to 56.39, making them suitable as natural anti-UV filters in sunscreens and demonstrating to directly extend the shelf life of fruits like bananas by about 7 days, showing their potential as a food preservative. The prepared HP-LNPs have good potential for industrial applications in bio-based adhesives, sunscreens, and biomedicine due to their favorable antioxidant and UV-blocking properties.

Lignin has been increasingly explored as a renewable component in adhesive formulations due to its aromatic structure, which enhances bonding strength and provides a sustainable alternative to petroleum-based resins. Zhan and coworkers synthesized a high-performance, sustainable lignin-based composite adhesive by leveraging the benefits of a deep eutectic solvent (DES) to modify the lignin structure and enable its effective reaction with furfural as a replacement for phenol and formaldehyde. DES (Choline chloride/urea with K_2_CO_3_) and alkaline lignin were reacted in a 2:1 optimum mass ratio for modification of lignin, and furfural was added for the preparation of lignin–furfural adhesive (DLF) [143]. After that, the adhesive, obtained after a 6-h reaction, was applied to a paulownia plate for the adhesion performance test. The adhesive strength of the modified composite achieved 4.54 MPa at room temperature, while hot-pressed at 0.13 MPa, and at −30 °C, the strength increased by 16.74% compared to room temperature. Tensile strength properties were studied using balsa wood, and DLF–balsa wood composites showed significantly higher strength (3.61 MPa) than plain balsa wood (1.73 MPa). In addition to enhanced mechanical properties, the lignin-based composite adhesive exhibited considerably improved thermal and flame-retardant properties compared to traditional petroleum-based adhesives and coatings.

**Figure 64:**
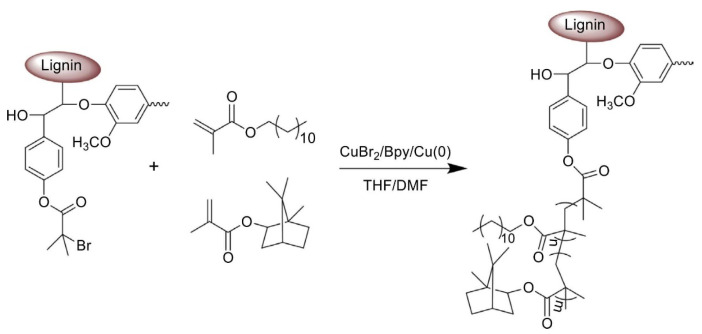
Synthesis of OL-*g*-P(IBOMA-*co*-LMA) copolymers [144]. Used with permission, copyright 2023 Elsevier.

Wu and coworkers developed a lignin-based copolymer as a promising sustainable alternative to petroleum-based thermoplastic elastomers and adhesives [144]. Organosolv lignin was modified to synthesize a lignin macromolecular initiator (OL-Br) by esterification of lignin hydroxyl groups with 2-bromoisobutyryl bromide (BiBB). Afterward, two biomass-derived monomers, lauryl methacrylate (LMA) from plant oil and isobornyl methacrylate (IBOMA) from pine sap, were grafted onto the lignin macroinitiator via ARGET ATRP (activators regenerated by electron transfer for atom transfer radical polymerization) to form lignin-*graft*-poly(isobornyl methacrylate-*co*-lauryl methacrylate) copolymers (OL-*g*-P(IBOMA-*co*-LMA), Figure 64). Different mass ratios of lignin and IBOMA were used to prepare various elastomers. The thermal stability and the glass transition temperature (*T*_g_) of the copolymers were increased with the addition of lignin. Moreover, the molar ratio of IBOMA to LMA in the copolymers also influenced their thermal and mechanical properties. Increasing the IBOMA content led to higher glass transition temperatures and improved tensile properties. The OL-*g*-P(IBOMA-*co*-LMA) copolymers exhibited adhesive properties on various substrates, such as glass, metal, plastic, and paper, making them potential all-biomass adhesives with UV absorption abilities.

**Figure 65:**
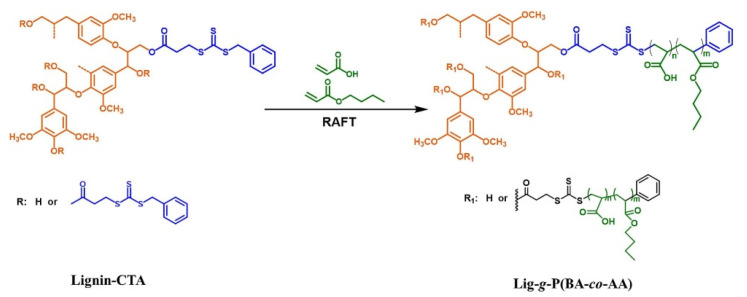
Preparation of Lignin-*graft*-poly(*n*-butyl acrylate-*co*-acrylic acid) copolymer [145]. Used with permission, copyright 2023 Elsevier.

In another study, Ou and co-workers developed lignin-graft copolymers [Lignin-*graft*-poly(*n*-butyl acrylate-*co*-acrylic acid)]. via reversible addition–fragmentation chain transfer (RAFT) polymerization (Figure 65) [145]. These lignin-based materials exhibited good thermal stability and tunable glass transition temperatures (*T*_g_). The mechanical properties, including tensile strength, elongation at break, Young’s modulus, and adhesive properties, can be readily adjusted by varying the *n*-butyl acrylate (BA)/acrylic acid (AA) monomer ratio and lignin content. A higher BA content resulted in lower *T*_g_ due to increased chain mobility, while grafted copolymers generally showed higher *T*_g_ values than their linear counterparts owing to restricted chain flexibility. At a lignin content of 7.7 wt.%, the copolymer demonstrated exceptional stretchability with low tensile stress, whereas 11.8 wt.% lignin incorporation yielded the highest tensile strength (14.0 MPa) with moderate elongation (510%), emphasizing the reinforcing role of lignin. Additionally, the 7.7 wt.% lignin-containing sample exhibited the highest adhesion strength (8.7 MPa), surpassing that of neat samples due to the physical crosslinking effect of lignin. Strong adhesion was also observed on various substrates, particularly wood, aluminum, and copper, highlighting the material’s potential for broad adhesive applications.

### 5.2. Lignin as Filler or Compatibilizer in Blends and Composites

**Figure 66:**
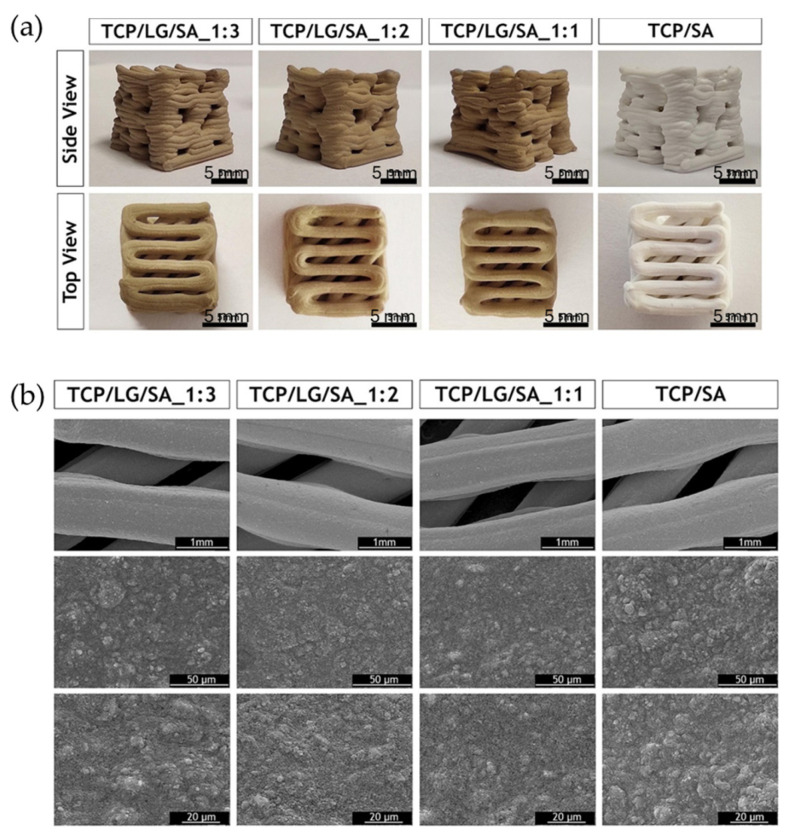
(**a**) Representative macroscopic images of the different scaffolds (side and top views) and (**b**) SEM images showing the morphology and surface topography of the 3D scaffolds produced at different magnifications [148]. Used with permission, copyright 2023 Elsevier.

Additive manufacturing, also known as 3D printing, has emerged as a state-of-the-art technique for fabricating complex, customizable structures across a wide range of materials, including polymers, ceramics, and metals. More recently, the 3D printing of lignin-based composites has garnered significant interest due to lignin’s abundance, renewability, and potential to replace fossil-derived polymers while enabling tailored mechanical and functional properties [146,147]. A novel 3D scaffold was introduced by Silva-Barroso et al. to overcome challenges such as poor mechanical strength, limited bioactivity, and inadequate cellular integration in bone tissue regeneration [148]. In the study, sodium alginate (SA) and lignin (LG) were mixed in different ratios (25–50 wt.% of LG) to form the polymer matrix and reacted with tricalcium phosphate (TCP) to prepare TCP/LG/SA scaffolds through the layer-by-layer deposition method. The macroscopic images (Figure 66a) showed a color change in the 3D scaffolds from white to light brown with lignin addition, and SEM images (Figure 66b) revealed an irregular, rough surface, providing more anchorage points that enhance bioactivity, protein adsorption, and cell adhesion. The addition of 33.3 wt.% lignin improved the mechanical resistance of the scaffolds, resulting in a 15% increase in compressive strength compared to TCP/SA scaffolds without lignin. Moreover, TCP/LG/SA composites improved the wettability of the 3D-printed TCP/SA composite scaffolds while maintaining good biocompatibility and osteogenic potential, supporting the use of lignin in developing 3D scaffolds for bone tissue regeneration applications. However, further in vivo evaluation of TCP/LG/SA scaffolds is essential to validate their potential for clinical applications.

Norouzi and co-workers developed electrospun scaffolds using lignin-modified PLGA [poly(L-lactide-*ε*-caprolactone)] and PLGA/Fibrin blends, both of which are biocompatible and biodegradable copolymers [149]. Plant-derived alkali lignin and animal-derived fibrin were used to prepare various scaffold formulations. The scaffold formulations included a 10% solution of pure PLGA, along with 10% polyblend solutions combining PLGA with either fibrin or lignin in ratios of 9:1, 8:2, and 7:3. Additionally, 10% polyblend solutions incorporating PLGA, fibrin, and lignin were prepared in ratios of 7:2:1, 6:2:2, and 5:2:3. Pure PLGA scaffolds exhibited the highest tensile strength (117.56 MPa) while the addition of lignin, fibrin, or fibrin/lignin to PLGA decreased the tensile strength of the scaffolds. PLGA/10% lignin scaffolds showed the second-highest tensile strength (70.52 MPa), and increasing lignin content in these scaffolds reduced their tensile strength from 70.52 MPa to 52.70 MPa and their elongation at break from 87.08% to 20.83%. Similarly, increasing lignin in PLGA/Fibrin/Lignin scaffolds decreased their tensile strength from 64.46 MPa to 27.38 MPa, Young’s modulus from 8.85 MPa to 5.68 MPa, and elongation at break from 28.29% to 20.83%, indicating their potential as appropriate candidates for engineering various tissues. In vitro biological responses of the human Adipose-derived Stem Cells to the scaffolds were studied. However, more comprehensive studies and a full understanding of the long-term performance are required for the targeted applications.

**Figure 67:**
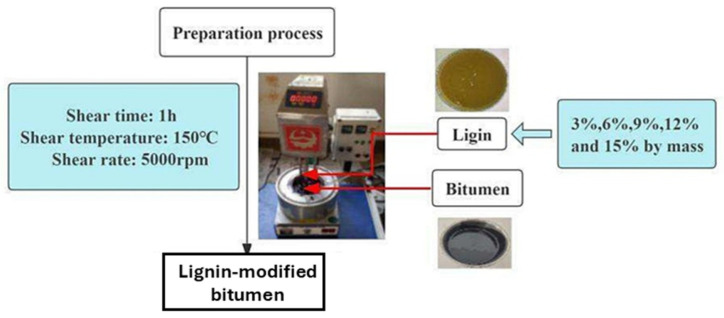
Experimental plan of the study [150]. Used with permission, copyright 2023 Elsevier.

Bitumen (asphalt), a binder in pavement materials, was modified by Su et al., adding lignin to improve its aging resistance and thermal-oxidative stability [150]. Lignin-modified bitumen samples were prepared by adding different percentages of lignin (0–15% by mass) to the base bitumen. The modification was performed through physical blending, where lignin was added to the molten bitumen and mixed using a high-speed shear mixer (Figure 67). Dynamic shear rheology (DSR) tests were conducted to find complex shear modulus, phase angle, rutting factor, and complex modulus aging index (CAI) at different temperatures (58 °C, 64 °C, and 70 °C) before and after short-term aging. The complex shear modulus and rutting factor values of lignin-modified bitumen before and after aging were higher than those of unmodified bitumen, indicating improved elastic recovery, deformation, and rutting resistance. The CAI values of lignin-modified bitumen were lower than those of pure bitumen at all temperatures, suggesting better resistance to short-term thermal-oxidative aging. TGA data revealed that the initial decomposition temperature of lignin-modified bitumen was lower due to the decomposition of lignin earlier during the thermal-oxidative aging process. Lignin acts as a “sacrificial agent” helping slow down bitumen aging. However, further studies on low-temperature cracking resistance and moisture susceptibility are required for lignin-modified bitumen before its application as a road construction material.

**Figure 68:**
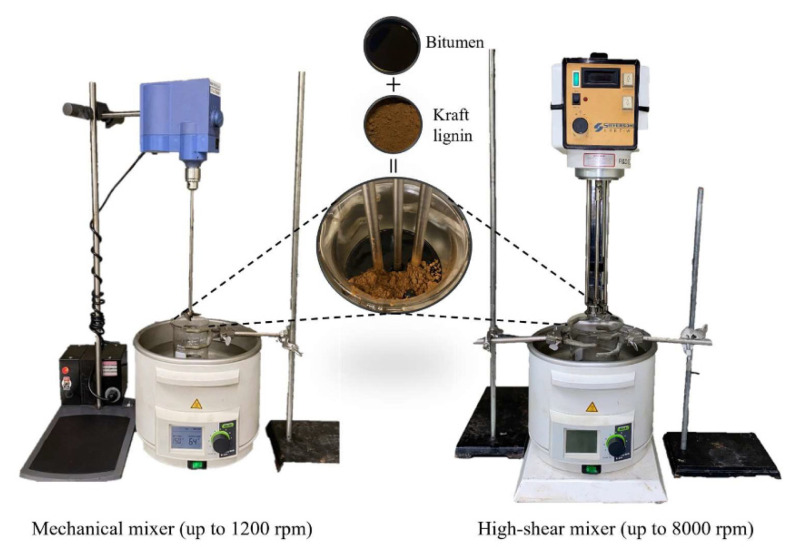
Blending kraft lignin and bitumen using a mechanical mixer (MM) and high-shear mixer (HSM) in the oil bath [151]. Under a Creative Commons Attribution (CC BY) license (https://creativecommons.org/licenses/by/4.0/ accessed on 27 April 2025).

**Figure 69:**
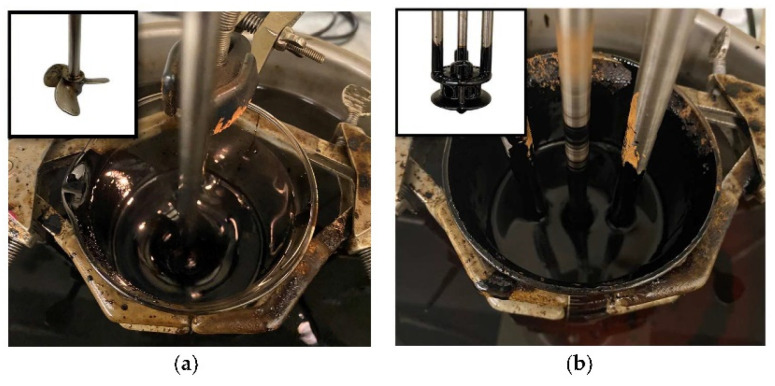
Comparison between (**a**) the mechanical mixer (MM) and (**b**) the high-shear mixer (HSM) during the blending process [151]. Under a Creative Commons Attribution (CC BY) license (https://creativecommons.org/licenses/by/4.0/ accessed on 27 April 2025).

Rezazad Gohari et al. investigated the use of kraft lignin as a modifier for bitumen [151]. Kraft lignin, derived from softwood (0–20 wt.% of lignin), was added to a PG 58S-28 bitumen using two blending protocols (Figure 68). The properties of the lignin-modified bitumen, produced with a mechanical mixer (at 1000 rpm) and a high-shear mixer (at 5000 rpm), were then evaluated. When processing pure bitumen, the mechanical mixer method resulted in a higher final viscosity than the high-shear mixer due to the loss of volatile compounds (Figure 69). DSR analysis at 58 °C, 64 °C, and 70 °C showed that the mechanical mixer method yielded higher complex shear modulus and rutting factor values, which further increased with lignin addition. TGA data indicated improved thermal stability with lignin incorporation, while the mechanical mixer method exhibited higher thermal decomposition than the high-shear mixer. Lignin content and processing conditions had a minimal impact on *T*_g_, but *T*_g_ remained lower than that of virgin bitumen. Overall, the study highlights the influence of lignin loading and processing conditions on the lignin-modified bitumen.

**Figure 70:**
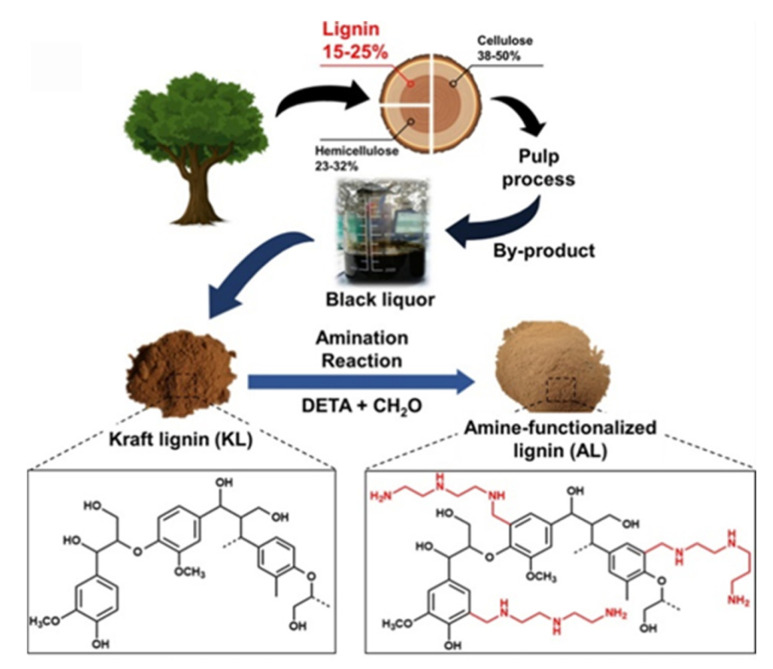
Schematic illustration of the process used to fabricate AL via the amination reaction [152]. Used with permission, copyright 2023 American Chemical Society.

Antioxidants such as *N*-(1,3-dimethylbutyl)-*N*′-phenyl-p-phenylenediamine (6PPD) are combined with rubber to produce automotive tires [152]. 6PPD is a toxic compound that has the critical drawback of being utilized as an antioxidant. As a solution, Chung et al. introduced amine-functionalized lignin (AL) as an eco-friendly antioxidant to replace 6PPD [153]. Kraft lignin (KL) was chemically modified via amination with diethylenetriamine (DETA) and formaldehyde, grafting primary and secondary amine groups to enhance the radical scavenging ability of lignin (Figure 70). The AL particles (0–4 phr) were incorporated into natural/butadiene rubber (NB) using an internal mixer, followed by sulfur crosslinking and compression molding. Compared to 6PPD and KL, AL-modified rubber exhibited superior thermal and ozone aging resistance, indicating improved antioxidant properties. AL also participated in the vulcanization reaction, promoting sulfur activation, increasing the curing rate, and enhancing crosslink density. Notably, NB/AL (2 phr) showed a 28% higher crosslink density than NB/6PPD. In mechanical testing, AL-modified rubber outperformed 6PPD and KL, with a higher 50% modulus value, suggesting improved stiffness and elasticity. After thermal aging at 120 °C for 24 h, NB/AL (2 phr) retained more tensile strength (36.2% decrease) and elongation (46.9% decrease) than 6PPD and KL-modified rubber, likely due to a balance of crosslinking hardening and chain scission softening. Overall, amine-functionalized lignin improved both the antioxidant and mechanical properties of rubber, demonstrating its potential as a sustainable alternative to 6PPD. However, further environmental testing and long-term performance evaluation are necessary for broader industrial applications.

**Figure 71:**
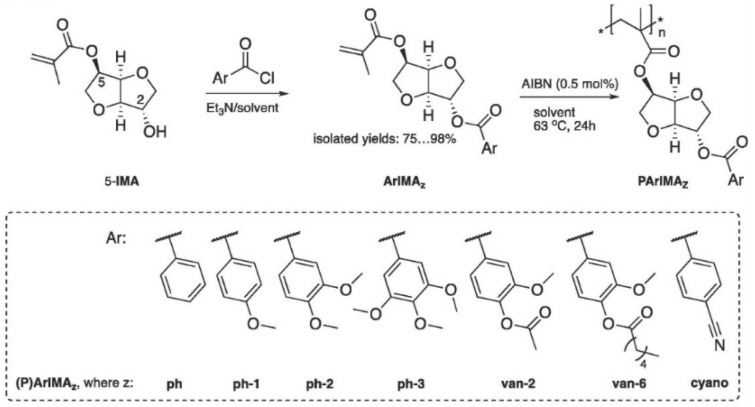
Synthesis and polymerization of isosorbide methacrylates substituted with different lignin derivatives with phenyl groups (IMA: isosorbide methacrylates, Ar: the aromatic side group in lignin) [154]. Under a Creative Commons Attribution (CC BY) license (https://creativecommons.org/licenses/by/4.0/ accessed on 27 April 2025).

Laanesoo et al. developed a novel approach to bio-based high-performance polymers by combining isosorbide, a rigid bicyclic diol derived from glucose, with lignin-derived phenyl aromatic groups such as vanillic, syringic, and benzoic acids [154]. Methacrylate monomers were synthesized via esterification of isosorbide with these lignin derivatives and subsequently polymerized through free radical polymerization to produce aromatic polymethacrylates (PArIMAs, Figure 71). The resulting polymers exhibited high glass transition temperatures (*T*_g_) ranging from 80 to 168 °C and demonstrated thermal stability up to 268 °C, attributed to the rigidity of the isosorbide core and the restricted chain mobility from the aromatic side groups. Dynamic rheology studies confirmed the materials’ excellent shear stability, with constant complex modulus and viscosity maintained over time and at elevated temperatures up to 200 °C. These findings indicate that the lignin-derived polymethacrylates are thermally stable, resistant to degradation, and melt-processable, making them promising candidates for sustainable high-performance plastic applications.

**Figure 72:**
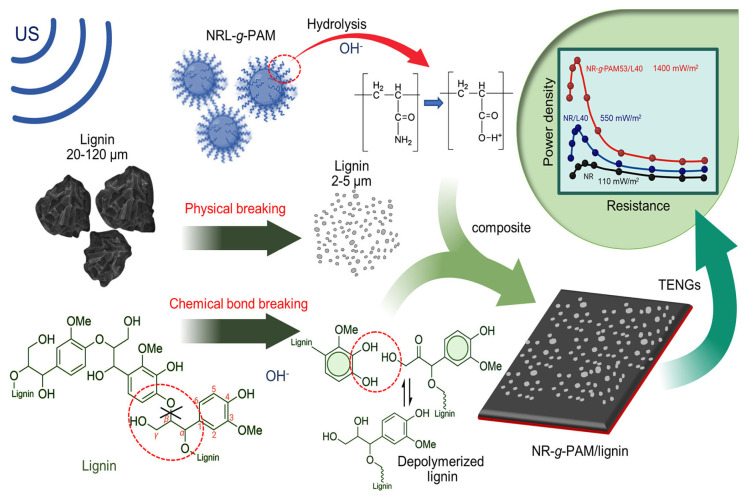
Schematic demonstration of the ultrasound-induced transformation of the lignin to a triboelectric-active material for NR-based TENGs [156]. Used with permission, copyright 2023 American Chemical Society.

The environmental impact of fossil-based energy has intensified the demand for clean and sustainable alternatives. Triboelectric nanogenerators (TENGs) offer a promising solution by harnessing sustainable energy sources for electricity generation [155]. Nanthagal et al. demonstrated the use of lignin as a triboelectric active material in natural rubber (NR)-based TENGs [156]. Lignin was depolymerized using ultrasound irradiation in a basic solution, producing micrometer-sized particles with increased polar functional groups to enhance lignin’s performance (Figure 72). These particles were incorporated into NR and NR grafted with polyacrylamide (NR-*g*-PAM) composites, where PAM acted as a stabilizer, improving dispersion and interfacial adhesion. The controlled coprecipitation of NR latex and lignin dispersion facilitated uniform integration. The incorporation of lignin significantly enhanced TENG power output, with NR/lignin and NR-*g*-PAM/lignin achieving 500% and 1300% improvements, respectively, compared to NR TENGs. However, TGA analysis showed no notable improvement in thermal stability. The mechanical properties of the composites played a crucial role in TENG performance. Under the glassy state, NR/lignin exhibited a lower storage modulus (*E*′), indicating that lignin behaved as a diluent due to agglomeration and low interfacial area. In contrast, NR-*g*-PAM/lignin showed a significant increase in *E*′, demonstrating a reinforcing effect that enhanced charge transfer efficiency and mechanical durability. Future work could explore surface modifications or crosslinking strategies to improve lignin dispersion further and enhance thermal stability, broadening its potential applications in next-generation TENGs.

Silica is commonly used as a reinforcing filler in polymers to enhance mechanical properties. However, its tendency to agglomerate and disperse poorly within the rubber matrix presents challenges. To address this, Chowdhury and coworkers partially replaced silica with lignin in a solution-grade styrene-butadiene rubber (SSBR) compound, optimizing the formulation for tire tread applications [157]. The Taguchi method was used to optimize the compound, considering factors such as SSBR vinyl content, silanization temperature, silica–lignin ratio, and silane coupling agent (SCA) dosage. Lignin incorporation improved the thermal stability of the rubber due to its high molecular weight aromatic fractions, leading to elevated decomposition temperatures. However, when silica replacement exceeded 20%, re-agglomeration of filler particles occurred, resulting in inferior dispersion. The reinforcement index (RI), calculated as the ratio of modulus at 300% elongation to 100% elongation, was used to assess filler effectiveness. The optimized compound, formulated with 58% vinyl SSBR, 160° C silanization temperature, a 40:10 silica–lignin ratio, and 8–10% SCA dosage, exhibited a higher RI, indicating better reinforcement and improved tensile properties compared to other compositions. Despite these promising results, long-term aging and durability studies are needed to fully establish lignin as a viable replacement for synthetic fillers in tire formulations.

**Figure 73:**
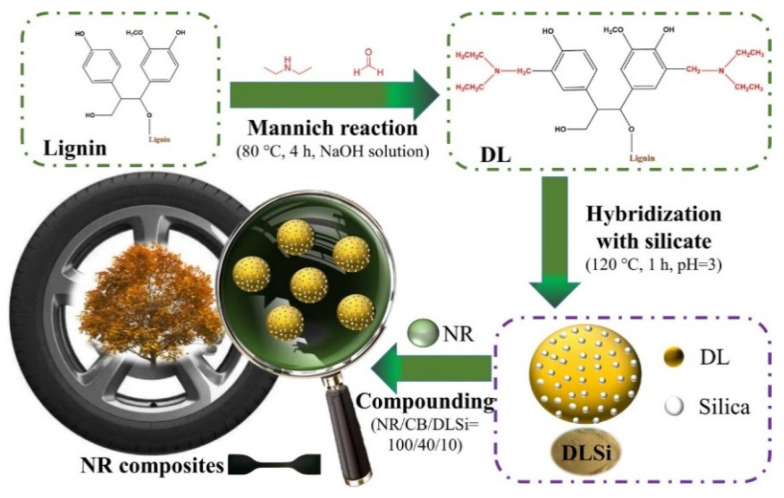
The schematic diagram for lignin modification and composite preparation [158]. Used with permission, copyright 2023 Elsevier.

**Figure 74:**
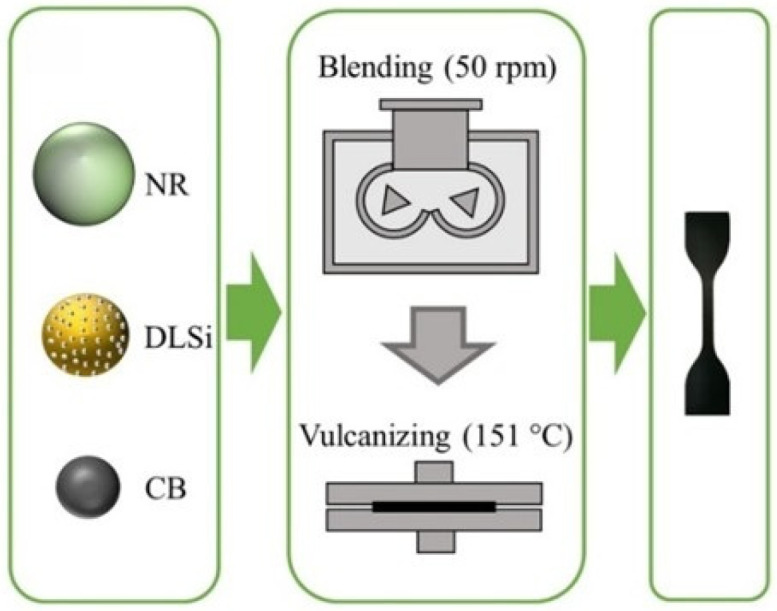
Preparation method of NR-DLSi composites by inserting modified lignin into natural rubber [158]. Used with permission, copyright 2023 Elsevier.

Qiu et al. developed lignin/SiO_2_ nano-hybrid fillers to reinforce natural rubber (NR) composites. Lignin was modified via the Mannich reaction with diethylamine and formaldehyde, producing diethylamine-grafted lignin (DL), which was then hybridized with SiO_2_ to form DLSi composites (Figure 73) [158]. The grafted diethylamine (18.55 wt.%) enabled lignin to ionize under acidic conditions, promoting strong interactions with negatively charged SiO_2_ and facilitating the formation of nano-hybrid particles. The DLSi1 hybrid (DL/SiO_2_ = 1:2) exhibited enhanced hydrophobicity, increasing the water contact angle from 64.9° to 91.1°. In NR composites, 20 wt.% of the total filler was replaced by DLSi1, and vulcanization was carried out at 151 °C (Figure 74). NR composites incorporating DLSi1 and carbon black (CB, 40 phr) demonstrated a storage modulus comparable to NR-CB composites in dynamic mechanical analysis (DMA) tests, indicating strong rubber–filler interactions. Additionally, the lower tan_δmax_ in NR-DLSi1 suggested that the hybrid filler restricted the viscoelastic relaxation of NR. Mechanical testing showed that NR-DLSi1 had a tensile strength (23.7 MPa), 100% modulus (1.8 MPa), and elongation at break (565%) comparable to NR-CB (24.2 MPa, 1.8 MPa, and 536%, respectively), demonstrating that DLSi1 could replace 20% of CB without compromising performance. NR-DLSi1 also outperformed NR composites modified with SiO_2_ alone, highlighting the reinforcing effect of the hybrid filler due to its nano-size, uniform dispersion, and strong interfacial interactions within the NR matrix.

Campos et al. investigated the use of kraft lignin as a reinforcing filler in carboxylated nitrile rubber (XNBR) without any chemical or physical modification [159]. Using a mixture design of experiments (MDOE), they examined the influence of carboxyl group content (1–7%) and lignin loading (0–40 phr) on rubber–lignin interactions. The addition of zinc oxide facilitated ionic bonding with XNBR’s carboxyl groups, improving compatibility with lignin compared to nonpolar rubbers. The results showed a higher carboxyl content (7%), improved lignin dispersion, and enhanced rubber–lignin interactions. The storage modulus (*E*′) increased with lignin incorporation, demonstrating its reinforcing effect, particularly in XNBR with 7% carboxyl content. The glass transition temperature (*T*_g_) also shifted to higher temperatures with increasing carboxyl and lignin content. Shore A hardness, tear strength, and tensile properties improved significantly, with the X7L40 composite (7% carboxyl, 40 phr lignin) exhibiting the highest enhancements. Notably, stress at 100% strain in XNBR7 (7% carboxyl) rose from 1.7 to 6.3 MPa with lignin addition, whereas in XNBR1 (1% carboxyl), it increased from 1.2 to 1.9 MPa. These results highlight kraft lignin’s potential as a reinforcing filler in nitrile rubber, enhancing mechanical properties and overall performance. The foregoing examples are summarized in Table 4.

## 6. Conclusions

Lignin has emerged as a structurally diverse and functionally rich biopolymer that offers significant promise as a sustainable and performance-enhancing component in a wide range of polymeric systems. This review has surveyed the state-of-the-art strategies for lignin incorporation into thermosets, polyurethanes, PLA composites, and film and coating applications, with attention to both chemical and physical mechanisms of integration. In doing so, it becomes apparent that the specific chemical form of lignin, ranging from raw kraft lignin to fractionated and chemically modified derivatives, critically determines the processability, reactivity, and final properties of lignin-containing polymers.

Recent developments have demonstrated reactive modifications to enhance lignin’s compatibility and performance in epoxy and polyurethane systems by increasing available functional groups and reducing molecular heterogeneity. Likewise, physical strategies including nanoparticle dispersion or compatibilizer-assisted blending show great utility for film and composite applications, particularly where barrier, thermal, or UV properties are of concern. These approaches reflect a growing understanding of the importance of controlling lignin’s dispersion, interfacial adhesion, and chemical compatibility within diverse polymer matrices. However, despite these advances, a number of core challenges persist. The following key insights and future directions summarize the current state and critical advancements needed to realize lignin’s full potential:

**Versatility and challenges of lignin integration:**
  Lignin’s structural heterogeneity, particularly its wide molecular weight distribution and variable functional composition, continues to hinder reproducibility and scalability in polymer applications. Moreover, inconsistencies in lignin quality across extraction methods (e.g., kraft, organosolv, soda, hydrolysis) introduce variability in downstream performance, limiting industrial uptake.
**Impact of lignin form and modification strategy:**
  The chemical form of lignin (raw vs. modified) significantly influences its reactivity and compatibility with polymer matrices. Reactive modifications such as phenolation, oxyalkylation, and glycidylation have proven effective in enhancing functional group availability and reducing heterogeneity for improved performance in epoxy and polyurethane systems.
**Chemical vs. physical integration challenges:**
  There is a lack of clear distinction between covalent bonding and physical blending in many studies, with insufficient analytical validation of the proposed integration mechanisms. This ambiguity complicates efforts to develop reliable structure–property relationships and challenges the classification of lignin-based systems as true copolymers, hybrid networks, or filled composites. Future studies should prioritize quantitative methods, such as 2D NMR, MALDI-TOF, FTIR, kinetic analysis, and chromatographic fractionation, to identify and distinguish covalent grafting from surface interactions or entanglement. In the context of PLA composites, this distinction is especially relevant, given that both esterification and filler-type approaches are widely reported but not always experimentally validated.
**Importance of life-cycle assessments (LCAs) and techno-economic analyses (TEAs) implementation:**
  Beyond performance improvements, comprehensive life-cycle assessment (LCA) and techno-economic analysis (TEA) are essential for the real-world adoption of lignin-based materials [160,161]. TEA can help to optimize the production costs by evaluating the feasibility and scalability of lignin valorization routes including feedstock sourcing, process energy input, and purification requirements. Concurrently, LCA provides a quantitative evaluation of environmental impacts, including greenhouse gas emissions, water use, and toxicity. Incorporating TEA and LCA early in the development process will support scalable and sustainable innovations, ultimately accelerating the transition of lignin composites from laboratory research to commercial applications. However, few studies have systematically compared modified lignin-based products with conventional petrochemical analogs using these frameworks. Life-cycle assessments and techno-economic analyses are critical yet underused tools that can guide realistic implementation of lignin-based polymers. These tools support environmentally and economically sound material design and should be integrated into future development workflows.
**Sustainability and scalability considerations:**
  The use of greener solvents and catalysts during lignin modification, the recovery and reuse of unreacted lignin fractions, and the minimization of purification steps are all areas where process intensification can dramatically improve material sustainability profiles. Scalable modification strategies, particularly those avoiding halogenation, heavy metal catalysis, or multistep derivatization, will be key to industrial uptake.
**Emerging functional applications:**
  Beyond conventional thermosets and elastomers, lignin offers unique opportunities for next-generation applications. Its intrinsic UV-absorbing, flame-retardant, and antioxidant properties make it attractive for biomedical, packaging, and aerospace uses. The development of dynamic, reprocessable, and self-healing networks incorporating lignin, such as Diels–Alder-crosslinked PUs or vitrimeric lignin epoxies, represents a promising direction for coupling recyclability with mechanical robustness. Similarly, the intersection of lignin with nanotechnology, such as lignin-based nanoparticles or nanofibers, offers a route to high-performance hybrid materials with hierarchical structures and multifunctionality.
**Computational tools and high-throughput methods:**
  Computational modeling and machine learning approaches remain largely untapped in the lignin polymer space. These tools can assist in decoding complex relationships between lignin structure and polymer performance, optimize blend formulations, and predict processing behavior under varied conditions. Coupled with automated synthesis and high-throughput screening, such strategies can accelerate the design of lignin-based materials tailored for target applications.

Ultimately, the future of lignin in polymer science will be shaped by the field’s ability to reconcile its natural complexity with the precision demanded by engineered materials. This will require not only interdisciplinary collaborations among chemists, material scientists, and chemical engineers, but also engagement with stakeholders in forestry, agriculture, and biorefining to secure consistent, high-quality lignin streams. By embracing lignin’s potential rather than avoiding its variability, the field stands poised to unlock new generations of sustainable, high-performance polymers that redefine the materials landscape for a bio-based economy.

## Figures and Tables

**Table 1:** Summary of the lignin-based polyurethanes.

Lignin Type	Lignin Treatment/Modification Method	NCO Source	Application	Optimal Lignin wt.%	Reported Key Properties	Ref.
Pine alkali lignin	Purification	HDI	Coating	6.1%	Hardness: 260.1 MPa Elastic modulus: 4.7 GPa Good UV-blocking properties	[61]
Organosolv lignin	Dissolved in propylene carbonate	MDI [i/h ratio = 1]	Rigid and flexible foams	20%	95% shape recovery after cyclic compression	[62]
Industrial alkali lignin	Fractionation	Octamethylene diisocyanate [i/h ratio = 1]	Rigid foams	15%	Tensile strength: 0.9 MPa Elongation at break: >500%	[63]
Lignin from sawdust	Catalytic upstream biorefining	pMDI	Rigid foams	30%	Compressive strength: >0.25 MPa	[64]
Alkali lignin	Hydroxylation	TDI	Elastomers	22.2%	Tensile strength: 25.2 MPa Elongation at break: 500%	[65]
Hydrolyzed lignin	Acid-catalyzed phenolation	pMDI or HDI [i/h ratio = 0.95]	Films and adhesive	50%	Tensile strength: 47.6 MPa	[66]
Kraft lignin	Liquefaction	HDI [i/h ratio = 1]	Rigid foams	60%	Compressive stress: 86.80 kPa Compressive strain: 70% Thermal conductivity: 0.0401 W/mK	[67]
Enzymatic hydrolyzed lignin	Liquefaction	HDI	Rigid foams	NA	>99% oil–water separation Adsorption capacity: 98.2 mg/CuSO_4_ g	[68]
Lignin oligomers from sawdust	Reductive catalytic fractionation and oxypropylation	NA	NIPU films	17%	Tensile strength: 12.5 MPa Young’s modulus: 63 MPa	[69]
Black liquor lignin	Amination	NA	NIPU foam	NA	Higher hydroxyl number in amine-modified lignin	[70]
Enzymatic hydrolyzed lignin	Epoxidation	NA	NIPU film	40.36%	Tensile strength: 20.25 MPa Young’s modulus: 280 MPa Toughness: 5.51 MJ/m^3^	[71]
Kraft lignin	Functionalization with APTES	NA	WPU films	0.5%	Tensile strength: 8.77 MPa Elongation at break: ~25%	[72]
Enzymatic hydrolyzed lignin	Solvent shifting	Isophorone diisocyanate (IPDI)	WPU emulsions	5%	Tensile strength: >25 MPa Young’s modulus: 322 MPa	[73]
Lignosulfonate	NA	IPDI	WPU composites	7%	Tensile strength: 5.57 MPa Antibacterial activity: 97.27% for *E. coli*	[74]

**Table 2:** Summary of thermoset and crosslinked polymer networks synthesized from lignin-based monomers and oligomers.

Lignin Type	Lignin Treatment/Modification Method	Resin Matrix	Application	Optimal Lignin wt.%	Reported Key Properties	Ref.
Enzymatic hydrolyzed lignin	Glycidylation and multi-carboxylation	Epoxied soya bean oil, citric acid	Epoxy thermosets	80%	Ultimate tensile strength: 33.2 ± 2.1 MPa Tensile modulus: 480.5 ± 41.7 MPa	[82]
Enzymatic lignin	Glycidylation and esterification	PEG 400 and base asphalt	Epoxy asphalt as a pavement material	20%	Ultimate tensile strength: 331.61 MPa Tensile strain: 382.4%	[83]
Industrial alkali lignin	Fractionation and glycidylation	Polyetheramine (JD400), diglycidyl ether (BADGE)	Epoxy thermosets	10%	Ultimate tensile strength: 5.42 ± 0.34 MPa Elongation at break: 255.32 ± 3.16%	[84]
Black liquor lignin	Mannich reaction	Bisphenol A-based epoxy resin (E901)	Epoxy composite film	70%	Ultimate tensile strength: 30 MPa	[85]
Kraft lignin (KL)	Ball milling (BMKL) Ultrasonication (NLH) Glycidylation (GKL)	Diglycidyl ether of bisphenol A (DGEBA), and curing agent aliphatic polyoxypropylene α,ω-diamine (Jeffamine D-230)	Epoxy resin	3% KL	Ultimate tensile strength: 89.1 ± 5.7 MPa Strain at break: 6.3 ± 0.4%	[86]
				6% BMKL	Ultimate tensile strength: 71.6 ± 4.3 MPa Strain at break: 5.9 ± 0.4%	
				9% NLH	Ultimate tensile strength: 80.5 ± 6.1 MPa Strain at break: 7.4 ± 0.6%	
				9% GKL	Ultimate tensile strength: 80.8 ± 1.5 MPa Strain at break: 6.8 ± 0.3%	
Kraft lignin	Fractionation and glycidylation	DGEBA, and curing agent polyether amine (D-400)	Epoxy resin	60%	Ultimate tensile strength 14.7 ± 0.2 MPa Elongation at break 194.3 ± 5.7%	[87]
Kraft lignin	Epoxidation	Amine-based NT-1515 curing agent, and Bamboo fibers	Epoxy resin	25% fiber addition	Storage modulus: 2.63 GPa Flexural strength: 116 MPa Flexural modulus: 5.69 GPa	[88]
Kraft lignin	Epoxidation	DGEBA, and tannic acid	Adhesive	5%	Shear strength: 10.57 MPa	[89]
Acidic lignin	Grafting silicon chains and etherification	Bisphenol F epoxy resin (DGEBF) and polyether amine (D230)	Epoxy resin composites (electronic packaging)	3%	Compared to neat EP: impact strength ↑16.25%, tensile ↑25.81%, bending modulus ↑16.21%, and bending strength ↑42.43%	[90]
Kraft lignin	Fractionation using a solvent system	Formaldehyde [p/a ratio = 1.7]	PF resin	25% of the soluble fraction	Bonding strength > 4 MPa	[91]
Alkaline lignin	Treated with the DES system	Phenol and formaldehyde [p/a ratio = 1.2]	Supercapacitor electrodes	30%	Capacitance: 112.4 F/g Energy density: 3.9 Wh/kg Power density: 125 Wk/g	[92]
Kraft lignin	Phenolation	Formaldehyde [p/a ratio = 0.5]	Wood adhesive	30%	Shear strength 3.41 MPa	[93]
Kraft lignin	Periodate Oxidation	Phenol and formaldehyde [p/a ratio = 3.84]	Wood adhesive	49% unmodified lignin	Dry shear strength: >2.75 MPa	[94]
Alkali lignin	Treated with phosphoric acid and hydrogen peroxide (PHP-lignin)	Phenol and formaldehyde [p/a ratio = 0.5]	Wood adhesive	PHP-lignin	Ultimate bonding strength 2.16 MPa	[95]

**Table 3:** Summary of the lignin-based PLA polymers.

Lignin Type	Lignin Treatment Method	Monomer/Polymer Matrix	Application	Optimal Lignin Details	Reported Key Properties	Ref.
Commercial lignin	NA	Lauryl methacrylate	Composite films	5 wt.% Lg-*g*-PLMA	Strain at break: 8.1% Toughness: 245.6 MJ/m^3^	[102]
Kraft lignin	Ethyl acetate extraction and methylation	Glycidol	PLA films	10 wt.%	Elongation at break: 297.7% Toughness: 39.92 MJ/m^3^ Blocked 99.52% of UV-A	[103]
Alkali lignin	NA	PLA granules	PLA films and sunscreen	90 wt.%	Stronger UV shielding, in the UV-A range	[104]
Softwood kraft lignin	Fractionation, followed by ultrasonication (SLNs)	Poly(L-lactic acid) (PLLA)	Films	1 wt.%	Enhanced crystallization rate, crystallinity, and nucleation density with SLNs	[105]
Commercial lignin	Etherification with EGDE or PEGDE	PLA	Packaging material	5 phr with EGDE	O_2_ barrier property: 58.3% Tensile strength: >60 MPa	[106]
Lignin from corncob refinery residues	Alkali pretreatment	Epoxidized natural rubber/PLA	Disposable tableware, packaging materials and toys	20 wt.%	Tensile strength: 45.31 MPa Young’s modulus: 2.36 GPa	[107]
Kraft lignin	Grafting 2-ethylhexyl acrylate (e-Lignin)	PLA	3D printing	2.5 wt.% with e-Lignin	Tensile strength: >60 MPa Young’s modulus: >3 GPa Toughness: >2 MJ/m^3^	[108]
Kraft lignin and kraft nano-lignin	NA	PLA, L-Lactide	Films	0.5 wt.% nano-lignin	Hardness: 265 MPa Elastic modulus: 6098 MPa	[109]
Alkali lignin	Laccase enzymatic degradation (EL)	PLA	3D printing	1.25 wt.% with EL	Tensile strength: >40 MPa Elastic modulus: 2.2 GPa	[110]
Soda lignin from Beech sawdust	Steam-explosion pretreatment and modified with PEG	PLA	3D printing	10 wt.% PEG-modified lignin	Tensile strength: >60 MPa Elongation at break: >6% Elastic modulus: >3.5 GPa	[111]
Commercial lignin	Urethanization of lignin with HDI	PLA	Films	14 wt.%	Tensile strength: 36.5 MPa Elongation at break: 110% Elastic modulus: 5.56 GPa	[112]
Alkali lignin	Etherification	PLA	Composite films	3 wt.%	Tensile strength: >25 MPa Young’s modulus: 1.3 GPa	[113]
Alkali lignin	Fractionation and Dopamine-grafting (OL@DA)	PLA	Composite films	0.5 wt.% of OL@DA	Tensile strength: >50 MPa Elongation at break: 14% Impact strength: ~16 kJ/m^3^	[114]

**Table 4:** Summary of the preparation, applications, and properties of other lignin-based polymers.

Lignin Type/ Source	Lignin Treatment/Modification Method	Polymer Matrix	Application	Optimal Lignin Details	Reported Key Properties	Ref.
Dealkaline lignin	Microencapsulated in epoxidized soybean oil	Poly(butylene adipate-*co*-terephthalate) (PBAT), Tannic acid	Films	1 wt.%	Tensile strength: 7.27 MPa Elongation at break: 439.87%	[120]
Alkali lignin	Enzymatic modification/decolorization	Keratin	Films	50 wt.% of DML	Tensile strength: 14.8 MPa Elongation at break: 23.7%	[121]
Alkali lignin	Grafting *n*-octadecyl isocyanate	Castor oil and 1,12-diaminododecane	Films	20 wt.%	Tensile strength: 35 MPa Elongation at break: 80% Toughness: >600 J/m^3^	[123]
Dealkaline lignin	Cyanoethylation	Poly(vinyl alcohol) (PVA)	Films	1 wt.%	Tensile strength: 31.1 MPa Elongation at break: 218%	[124]
Sodium lignin sulfonate	Electrostatic adsorption	Chitosan	Films	1 wt.%	Endotoxin adsorption efficiency: 87.6%	[125]
Lignin from Radiata Pine	Treated with ternary DES to form lignin-containing cellulose nanofibrils (LCNF)	Cellulase	Films	LCNF treated at 130 °C	Tensile strength: 52.0 MPa Elongation at break: 8.4% Tensile modulus: 4.2 GPa	[126]
Lignin from Moso bamboo	Pre-hydrolysis and treated with DES	Starch	Films	2 wt.%	Tensile strength: 48.9 MPa Elastic modulus: 2288.8 MPa	[127]
Kraft lignin from *Eucalyptus* wood	Fractionation with ethyl acetate	Starch	Films	4 wt.% soluble fraction of lignin	Tensile strength: >14 MPa Elastic modulus: 69.24 MPa	[128]
Black liquor lignin from rice straw	Alkylation	Chitosan	Films	8 wt.%	DPHH scavenging activity: 93.25%	[129]
Kraft lignin	Phenolation	Cellulose and PVA	Films	2 h treated lignin	Tensile strength: >40 MPa Toughness: 55 MJ/m^3^	[130]
Dealkaline lignin	Amination via Mannich reaction	PVA	Films	30 wt.%	Tensile strength: 3.6 MPa Elongation at break: 107.8%	[131]
Lignin from Masson pine	DES pretreatment (choline chloride and lactic acid)	Cellulose	Films	Treated at 140 °C for 4 h	Tensile Stress: >50 MPa 100% absorption of UVB	[132]
Organosolv lignin from potato crop residues	Solvent extraction	Starch	Films	1 wt.%	Tensile strength: >2 MPa Young’s modulus: 69 MPa	[133]
Organosolv lignin	Incorporation of silver-embedded clay	PVA	Films	5 wt.%	Tensile strength: 28.56 MPa Elongation at break: 59%	[134]
Softwood kraft lignin	NA	Poly(caprolactone) (PCL)	3D-printed dressing material	10 wt.%	Bacterial adhesion reduction: 96.6%	[135]
Enzymatic hydrolysis lignin	Functionalization with Ti_3_C_2_T_x_	NA	Coating	33.3 wt.%	Oil–water separation efficiency: >99.5%	[136]
Enzymatic hydrolysis lignin	Hydrothermal treatment with Fe_3_O_4_	Polydimethylsiloxane (PDMS)	Coating	25 wt.%	Oil/organic wastes adsorption capacity: 17.17–24.78 g/g	[137]
Industrial lignin	Enzymatic and alkaline treatment	NA	Adhesives and sunscreens	5 wt.%	DPHH scavenging activity: 76.12% UVA protection	[142]
Alkali lignin	DES pretreatment	Furfural	Adhesives/coatings	33.3 wt.%	Bonding strength: 4.54 MPa	[143]
Organosolv lignin	Esterification	Lauryl methacrylate and isobornyl methacrylate	Elastomers and adhesives	16.5 wt.%	Fracture strength: 6.58 MPa Elongation at break: 337%	[144]
Enzymatic lignin	Purification	*n*-butyl acrylate and acrylic acid	Elastomers and adhesives	11.8 wt.%	Tensile strength: 14.0 MPa Elongation at break: 510%	[145]
Kraft lignin	NA	Sodium alginate and tricalcium phosphate	3D-printed scaffolds	33.3 wt.%	Compressive strength: >20 MPa Modulus: 90 MPa	[148]
Alkali lignin	NA	Poly(L-lactide-ε-caprolcatone) (PLGA)	Scaffolds	10 wt.%	Tensile strength: 64.46 MPa Elongation at break: 28.29%	[149]
Herbal lignin	NA	Bitumen (asphalt)	Pavement binders	15 wt.% after aging at 58 °C	Complex shear modulus: 7432 Pa Rutting factor: >2.2 kPa	[150]
Kraft lignin from softwood	NA	Bitumen	Pavement binders	10 wt.% after aging at 58 °C	Complex shear modulus increased by 51% Rutting factor: 3.22 kPa	[151]
Kraft lignin	Amination	Natural/butadiene rubber	Elastomers/tires	2 phr	Tensile stress: 20 MPa Improved antioxidant and ozone aging resistance	[153]
Lignin derivatives	Esterification	Methacrylate	Coating	NA	Broad *T*_g_ range: 80–160 °C	[154]
Commercial grade kraft lignin	Depolymerization using ultrasound irradiation	Natural rubber and polyacrylamide	Triboelectric nanogenerators	40 wt.%	Power density: 1411 mW/m^2^	[156]
Commercial lignin	NA	Styrene-butadiene rubber	Tire treads	10 wt.%	Tensile strength: 14.7 MPa Elongation at break: 604%	[157]
Soda lignin	Amination	Natural rubber	Fillers in tires	~32 wt.%	Tensile strength: 23.7 MPa Elongation at break: 565%	[158]
Hardwood kraft lignin	NA	Carboxylated nitrile rubber	Fillers in rubbers	40 phr	Shore A hardness: 77 HA Tear strength: 64 kN/m Tensile strength: 16.5 MPa Elongation at break: 434%	[159]

## Data Availability

The original contributions presented in this study are included in the article. Further inquiries can be directed to the corresponding author.

## Abbreviations

The following abbreviations are used in this manuscript:

PUs	Polyurethanes
PLA	Polylactic acid
UV	Ultraviolet
HDIT	Hexamethylene diisocyanate trimer
PDMS	Polydimethylsiloxane
wt.%	Weight percentage
HIT	Hardness
EIT	Elastic modulus
PUFs	Polyurethane foams
PC	Propylene carbonate
MDI	Diphenylmethane-4,4′-diisocyanate
TGA	Thermogravimetric analysis
CUB	Catalytic upstream biorefining
RPFs	Rigid polyurethane foams
SEM	Scanning electron microscopy
DA	Diels–Alder
PCL	Polycaprolactone
*T*_g_	Glass transition temperature
*E*′	Storage modulus
TDI	Toluene diisocyanate
HL	Hydrolysis lignin
PhL	Phenolated lignin
pMDI	Poly[(phenyl isocyanate)-*co*-formaldehyde]
LP	Lignin-based polyols
PEG	Polyethylene glycol
PDA	Polydopamine
PA	Phytic acid
NIPUs	Non-isocyanate polyurethanes
CCs	Cyclic carbonates
5CCs	Five-membered cyclic carbonates
CO_2_	Carbon dioxide
RCF	Reductive catalytic fractionation
DETA	Diethylenetriamine
EDA	Ethylenediamine
FT-IR	Fourier Transform Infrared spectroscopy
GPC	Gel permeation chromatography
6CCs	Six-membered cyclic carbonates
EEHL	Epoxidized enzymatic hydrolysis lignin
LNIPU	Lignin/non-isocyanate polyurethanes
WPU	Waterborne polyurethane
APTES	3-aminopropyltriethoxy silane
LNPs	Lignin nanoparticles
*E. coli*	*Escherichia coli*
i/h ratio	The ratio of isocyanate to hydroxy functionalities
IPDI	Isophorone diisocyanate
DGEBA	Diglycidyl ether of bisphenol A
BPA	Bisphenol A
CA	Citric acid
ESO	Epoxidized soybean oil
GL	Glycidylated lignin
*T*_5%_	Degradation temperatures (5% weight loss)
EL	Enzymatic lignin
ECH	Epichlorohydrin
LER	Lignin epoxy resin
BADGE	Bisphenol A diglycidyl ether
LPAA	Lignin-based phenolic amine
KL	Kraft lignin
NLH	Nano-lignin
GKL	Glycidylated kraft lignin
BMKL	Ball-milled kraft lignin
3D	Three dimensional
PHMS	Polyhydromethylsiloxane
PF	Phenol-formaldehyde
LPF	Lignin-based phenol-formaldehyde
DES	Deep eutectic solvent
PFC	Phenolic resin carbon
PHP	Phosphoric acid and hydrogen peroxide
AL	Alkali lignin
p/a ratio	The ratio of phenol to aldehyde
LMA	Lauryl methacrylate
CROP	Cationic ring-opening polymerization
EL	Ethyl acetate-extracted lignin
THF	Tetrahydrofuran
GLY	Glycidol
BF_3_	Boron trifluoride
*T*_max_	Maximum weight loss temperatures
W/O	Water-in-oil
PLLA	Poly (L-lactic acid)
DCM	Dichloromethane
EGDE	Ethylene glycol diglycidyl ether
PEGDE	Poly (ethylene glycol) diglycidyl ether
ENR	Epoxidized natural rubber
EHA	2-ethylhexyl acrylate
ROP	Ring-opening polymerization
TPU	Thermoplastic polyurethane
ALK	Modified alkali lignin
PBAT	Poly (butylene adipate-*co*-terephthalate)
Ta	Tannic acid
PVA	Poly (vinyl alcohol)
CEL	Cyanoethylated lignin
CNWs	Cellulose nano-whiskers
AgNP	Silver nanoparticle
LS	Lignin sulfonate
TDES	Ternary deep eutectic solvent
LCNF	Lignin-containing cellulose nanofibrils
TPS	Thermoplastic starch
*w*/*v*	Weight per volume
DPPH	2,2-diphenyl-1-picrylhydrozyl
*C. albicans*	*Candida albicans*
CNF	Cellulose nanofibril
aL	Aminated lignin
Fe(NO_3_)_3_	Ferric nitrate
CL	Lignin-containing cellulose
AlCl_3_	Aluminum chloride
ZnCl_2_	Zinc chloride
HNTs	Halloysite nanotubes
CUR	Curcumin
PCL	Poly(caprolactone)
SSE	Semi-solid extrusion
DPA	*D*-panthenol
EHL	Enzymatic hydrolysis lignin
MCE	Mixed cellulose ester
CNC	Nanocellulose crystals
TiO_2_	Titanium dioxide
H_2_O_2_	Hydrogen peroxide
PTIO	2-phenyl-4,4,5,5-tetramethylimidazoline-3-oxide-1-oxyl
K_2_CO_3_	Potassium carbonate
DLF	Lignin–furfural adhesive
IBOMA	Isobornyl methacrylate
ARGET ATRP	Activators regenerated by electron transfer for atom transfer radical polymerization
RAFT	Reversible addition-fragmentation chain transfer polymerization
BA	*n*-butyl acrylate
AA	Acrylic acid
SA	Sodium alginate
TCP	Tricalcium phosphate
PLGA	Poly(L-lactide-ε-caprolactone)
DSR	Dynamic shear rheology
CAI	Complex modulus aging index
MM	Mechanical Mixer
HSM	High-Shear Mixer
6PPD	*N*-(1,3-dimethylbutyl)-*N*′-phenyl-p-phenylenediamine
DETA	Diethylenetriamine
PArIMAs	Aromatic polymethacrylates
TENGs	Triboelectric nanogenerators
NR	Natural rubber
SSBR	Solution-grade styrene-butadiene rubber
SCA	Silane coupling agent
RI	Reinforcement index
DL	Diethylamine-grafted lignin
CB	Carbon black
DMA	Dynamic mechanical analysis
XNBR	Carboxylated nitrile rubber
MDOE	Mixture design of experiments
NA	Not available
